# Supplementary material for: New Therapeutics in Alzheimer’s Disease Longitudinal Cohort study (NTAD): study protocol
Source: BMJ Open. 2022 Dec 14;12(12):e055135. doi: 10.1136/bmjopen-2021-055135 (PMC9756184; doi:10.1136/bmjopen-2021-055135)
Supplement: Author's manuscript [file bmjopen-2021-055135.draft_revisions.pdf]

BMJ Open is committed to open peer review. As part of this commitment we make the peer review history of every article we publish publicly available.

When an article is published we post the peer reviewers' comments and the authors' responses online. We also post the versions of the paper that were used during peer review. These are the versions that the peer review comments apply to.

The versions of the paper that follow are the versions that were submitted during the peer review process. They are not the versions of record or the final published versions. They should not be cited or distributed as the published version of this manuscript.

BMJ Open is an open access journal and the full, final, typeset and author-corrected version of record of the manuscript is available on our site with no access controls, subscription charges or pay-per-view fees (http://bmjopen.bmj.com).

If you have any questions on BMJ Open's open peer review process please email info.bmjopen@bmj.com

BMJ Open

The New Therapeutics in Alzheimer's Disease Longitudinal Cohort study (NTAD): study protocol

Journal:	*BMJ Open*
Manuscript ID	bmjopen-2021-055135
Article Type:	Protocol
Date Submitted by the Author:	02-Jul-2021
Complete List of Authors:	Lanskey, Juliette; University of Cambridge, MRC Cognition and Brain Sciences Unit<br>Kocagoncu, Ece; University of Cambridge, Department of Clinical Neurosciences and Cambridge University Hospitals NHS Foundation Trust, Cambridge Biomedical Campus<br>Quinn, Andrew; University of Oxford, Oxford Centre for Human Brain Activity, Wellcome Centre for Integrative Neuroimaging, Department of Psychiatry<br>Cheng, Yun-Ju; Lilly Corporate Center<br>Karadag, Melek; University of Cambridge, Department of Clinical Neurosciences and Cambridge University Hospitals NHS Foundation Trust, Cambridge Biomedical Campus<br>Pitt, Jemma; University of Oxford, Oxford Centre for Human Brain Activity, Wellcome Centre for Integrative Neuroimaging, Department of Psychiatry<br>Isaac, John; Johnson and Johnson London Innovation Center, Neuroscience External Innovation<br>Lowe, Stephen; Lilly Corporate Center<br>Perkinton, Michael; AstraZeneca, Neuroscience, BioPharmaceuticals R&D<br>Raymont, Vanessa; University of Oxford, Department of Psychiatry<br>Singh, Krish; Cardiff University, Cardiff University Brain Research Imaging Centre, School of Psychology<br>Woolrich, Mark; University of Oxford, Oxford Centre for Human Brain Activity, Wellcome Centre for Integrative Neuroimaging, Department of Psychiatry<br>Nobre, Anna; University of Oxford, Oxford Centre for Human Brain Activity, Wellcome Centre for Integrative Neuroimaging, Department of Psychiatry<br>Henson, Richard; University of Cambridge, MRC Cognition and Brain Sciences Unit; University of Cambridge, Department of Psychiatry<br>Rowe, James; University of Cambridge, Department of Clinical Neurosciences and Cambridge University Hospitals NHS Foundation Trust, Cambridge Biomedical Campus; University of Cambridge, MRC Cognition and Brain Sciences Unit
Keywords:	Protocols & guidelines < HEALTH SERVICES ADMINISTRATION & MANAGEMENT, Dementia < NEUROLOGY, Neurophysiology < NEUROLOGY, NEUROPHYSIOLOGY, NEUROLOGY, RADIOLOGY & IMAGING

The New Therapeutics in Alzheimer's Disease Longitudinal Cohort study (NTAD): study protocol

Juliette H Lanskey[1], Ece Kocagoncu[2], Andrew J Quinn[3], Yun-Ju Cheng[4], Melek Karadag[2], Jemma Pitt[3], John Isaac[5], Stephen Lowe[4], Michael Perkinton[6], Vanessa Raymont[7], Krish D Singh[8], Mark Woolrich[3], Anna C Nobre[3], Richard N Henson[1,9], James B Rowe[1,2] on behalf of the NTAD study group

Affiliations

1. MRC Cognition and Brain Sciences Unit, University of Cambridge, Cambridge, UK
2. Department of Clinical Neurosciences and Cambridge University Hospitals NHS Foundation Trust, Cambridge Biomedical Campus, Cambridge, UK
3. Oxford Centre for Human Brain Activity, Wellcome Centre for Integrative Neuroimaging, Department of Psychiatry, University of Oxford, Oxford, UK
4. Lilly Corporate Center, Indianapolis, USA
5. Neuroscience External Innovation, Johnson & Johnson Innovations, London, UK
6. Neuroscience, BioPharmaceuticals R&D, AstraZeneca, Cambridge, UK
7. Department of Psychiatry, University of Oxford, Oxford, UK
8. Cardiff University Brain Research Imaging Centre, School of Psychology, Cardiff University, Cardiff, UK
9. Department of Psychiatry, University of Cambridge, Cambridge, UK

Correspondence

Juliette Lanskey
MRC Cognition and Brain Sciences Unit,
15 Chaucer Road,
Cambridge, CB2 7EF, UK
Juliette.lanskey@mrc-cbu.cam.ac.uk

Words	3944
Figures	4
Tables	4

For peer review only

ABSTRACT

Introduction

With the pressing need to develop treatments that slow or stop the progression of Alzheimer's disease, new tools are needed to reduce clinical trial duration and validate new targets for human therapeutics. Such tools could be derived from neurophysiological measurements of disease.

Methods and Analysis

The New Therapeutics in Alzheimer's disease study (NTAD) aims to identify a biomarker set from magneto/electro-encephalographic that is sensitive to disease and progression over one year. The study will recruit 100 people with amyloid-positive mild cognitive impairment or early-stage Alzheimer's disease and 30 healthy controls aged between 50 and 85 years. Repeat measurements of the clinical, cognitive and imaging data (magnetoencephalography, electroencephalography and magnetic resonance imaging) of participants with Alzheimer's disease or mild cognitive impairment will be taken at baseline and at one year. To assess reliability of magneto/electro-encephalographic changes, a subset of 30 participants with mild cognitive impairment or early-stage Alzheimer's disease will undergo repeat magneto/electro-encephalographic two weeks after baseline. Clinical and cognitive assessment will be repeated at 2 years. Linear mixed models of baseline and longitudinal change in neurophysiology are the primary analyses of interest, supported by Bayesian inference. Additional outputs will include relative effect sizes for physiological markers, atrophy and cognitive change and the respective numbers needed to treat each arm of simulated clinical trials of a future disease modifying therapy.

Ethics and Data statement

The study has received a favourable opinion from the East of England Cambridge Central Research Ethics Committee (REC reference 18/EE/0042). Results will be disseminated through internal reports, peer-reviewed scientific journals, conference presentations, website publication, submission to regulatory authorities and other publications. Data will be made available via the Dementias Platform UK Data Portal on completion of initial analyses by the NTAD study group.

For peer review only

STRENGTHS AND LIMITATIONS OF THIS STUDY

Strengths:

- NTAD is a longitudinal, multicentre study of magneto/electro-encephalographic measures of Alzheimer's disease progression
- NTAD assesses the test-retest reliability of magneto/electro-encephalographic parameters
- All participants with early-stage Alzheimer's disease or mild cognitive impairment will be amyloid-positive

Limitations:

- Attrition during follow up may limit inferences
- Recruitment is from volunteer panels and clinical services that may not reflect national diversity of people affected by Alzheimer's disease

INTRODUCTION

With 44 million people living worldwide with dementia, treatments to slow or stop disease progression are urgently needed. Alzheimer's disease is the most prevalent dementia but despite rapid advances in therapeutics within preclinical models,[1,2] clinical trials remain expensive, slow and challenging[3–5] with high-profile failures.[6] Significant bottlenecks exist in early-phase trials, where efficacy rates are very low and costs rapidly escalate. To bridge the gap between animal models and the human disease, better tools are needed to quantify pathogenic and pathophysiological mechanisms in patients, *in vivo* providing evidence to pursue or discontinue trials of a candidate treatment. These tools should be safe, scalable and able to support early-phase trials over a short duration and viable budget.

Brain imaging is widely used to diagnose dementia and measure pathology in clinical trials. There are diverse imaging methods with differential sensitivity to brain structure, chemistry, pathology and function. For example, magnetic resonance imaging (MRI) is commonly used to measure structural changes related to Alzheimer's disease,[7] such as entorhinal cortex[8] and hippocampus volumes.[9] Positron emission tomography (PET) can quantify and localise Alzheimer pathology, through the use of ligands that bind to the aggregated tau protein,[10–14] beta-amyloid plaques[15] and neuroinflammation.[16]

The loss of synapses and synaptic plasticity is an early feature of Alzheimer's disease.[17–19] Indeed, measures of synaptic loss may be more sensitive than measures of atrophy to cognitive decline.[20,21] As synaptic changes occur early in Alzheimer's disease,[20] such measures could be sensitive to the earliest precursors of cognitive involvement. This accords with preclinical models in transgenic mice where network physiology and cognition are impaired before tangles or cell death.[22]

In contrast to preclinical models and post-mortem analysis, there are limited options to assess synaptic function in humans, *in vivo*. While PET imaging now offers ligands that indirectly quantify synaptic density, electroencephalography (EEG) and magnetoencephalography (MEG) measure neurophysiological properties that depend on synaptic integrity and function within local and large-scale brain networks. MEG can identify synaptic and local circuit impairments[23,24] and their impact on network dynamics in Alzheimer's Disease,[25–28] frontotemporal dementia,[24,29–32] and Lewy-body disease.[33] MEG and EEG therefore have potential to support and de-risk clinical trials of novel compounds. However, to enable

For peer review only

MEG as a viable biomarker in clinical trials, one must assess longitudinal MEG and test-retest reliability and harmonise MEG protocols across sites.

The New Therapeutics for Alzheimer's Disease (NTAD) study aims to identify viable MEG and EEG biomarkers for clinical trials. The NTAD consortium is a multicentre study established by the Dementias Platform UK and supported by the Medical Research Council, Alzheimer's Research UK and industry partners.

This paper describes the NTAD study and its protocol to acquire longitudinal MEG and EEG data in people with biomarker-positive mild cognitive impairment and early Alzheimer's disease.

Research Aims

The primary objective is to identify a biomarker set from neurophysiological MEG and EEG that is sensitive to the presence and progression of Alzheimer's disease. We aim to harmonise MEG protocols across sites to allow biomarker identification from pooled data. Such a neurophysiological biomarker, or biomarker set, should be related to cognitive function, have high test-retest reliability and be able to track disease progression over the duration of clinical trials. The sensitivity to disease progression should ideally also outperform current widely used biomarkers such as MRI and cognitive tests.

Our secondary objective is to identify a biomarker set that can predict disease progression, explaining individual differences in the future trajectory of disease (i.e. prognostic from baseline) and the variation in cognitive decline over time (i.e. mediating mechanisms).

Analysis

The analysis of MEG parameters will occur in two stages. Stage 1 analyses will identify baseline, disease-sensitive MEG parameters and assess their reliability using test-retest data. Stage 2 analyses will assess the sensitivity of these parameters to longitudinal change.

Stage 1 analyses will assess the sensitivity of the cross-sectional MEG parameters to group effects (using parametric or non-parametric frequentist tests and receiver operating characteristic analyses and their Bayesian analogues), their correlation to baseline cognition (using Pearson or Spearman's rank correlation coefficient analyses) and their test-retest reliability (using intraclass correlation coefficient analyses). Stage 2 longitudinal analyses will assess how these neurophysiological markers change over ~12 months (using linear mixed models of change) and compare their sensitivity (as accuracy and effects sizes) to disease progression with MRI and cognitive measures. Using multiple regression models, we will identify baseline biomarkers that are predictive of disease progression. *Post-hoc* exploratory analyses will examine the relationship between cognitive and imaging biomarkers as a function of: (i) variations in tau haplotype, (ii) polymorphisms of genes related to plasticity (e.g. brain-derived neurotrophic factor), (iii) Alzheimer's disease risk alleles (e.g. apolipoprotein e4), (iv) polygenic risk scores of Alzheimer's disease, and (v) plasma tau levels.

METHODS

Study Design

This is a repeated-measures, observational-design study with 130 participants tested at two sites: Cambridge University and Oxford University. The protocol includes two stages (Figure

For peer review only - http://bmjopen.bmj.com/site/about/guidelines.xhtml

1). The first, cross-sectional stage consists of baseline and test-retest sessions. Baseline sessions are comprised of clinical and neuropsychological assessments, an MRI scan and a MEG combined with EEG (M/EEG) scan. For the test-retest session, a subset of patients returns for a second M/EEG scan approximately two weeks after the first. Stage 2, at 12-months, repeats baseline sessions, followed by a 24-month clinical and neuropsychological reassessment of patients.

Participant recruitment and selection

Participants will be aged between 50 and 85 years, with similar numbers of men and women, with symptomatic mild cognitive impairment or Alzheimer's Disease (n=100) or normal cognition (n=30, Figure 1). Potential participants are identified using local registry data, regional memory clinics and Join Dementia Research. People completing other observational studies may also be invited to screening.

A participant information sheet detailing the study procedures is provided to candidate participants and a study partner (their 'informant'). The informant is someone who regularly sees the participant and is willing to attend and complete the assessments. Candidate participants are invited to a screening appointment where any further questions they may have are answered before they provide written, informed consent. The screening appointment allows further assessment of eligibility according to inclusion and exclusion criteria (Table 1). Eligible people proceed to Stage 1.

For peer review only

Group inclusion criteria	
**Patients**<br>Diagnosis of MCI or AD<br>Positive amyloid status (CSF or PET)<br>MMSE > 18<br>CDR =0.5 – 1<br>50-85 years	**Controls**<br>No neurological diagnosis<br>Known amyloid status (CSF or PET)<br>MMSE > 24<br>CDR =0<br>50-85 years
**General exclusion criteria (patients and controls)**	
Significant neurological disease, other than Alzheimer's disease, that may affect cognition or ability to complete the study	
Presence of any significant psychiatric disorder that could affect participation [68]	
Any clinically important abnormality that could compromise study participation	
A clinically significant illness, medical or surgical procedure, or trauma within 30 days prior to screening or baseline	
Known or suspected systemic infection	
Medications affecting cognition, unless on a stable dose for >30 days prior to baseline	
Rosen Modified Hachinski Ischaemic score ≥ 4	
History of seizure, except febrile seizures or single provoked seizure	
Head trauma resulting in protracted loss of consciousness, or serious infectious disease affecting the brain, within five years of screening and baseline	
Participation in a clinical trial of an investigational medicinal product	
Impairment of vision or hearing that could affect study participation	
Formal education ≤ 7 years	
Lack of mental capacity or other ability to consent	
Inability to read and write fluently in English	
Inability to walk 10 metres independently	
Contraindications to blood sampling	
Contraindications to lumbar puncture (e.g. spinal deformations) and amyloid PET scan	
Contraindication to MRI (including, but not limited to, claustrophobia; pregnancy; MR-incompatible pacemakers and other MR-incompatible, implanted medical devices)	
Metallic implants in the body that affect MEG recordings, as judged by the Investigator	

***Table 1 Group inclusion criteria and general exclusion criteria**. MCI=Mild Cognitive Impairment, AD=Alzheimer's Disease, MMSE=Mini Mental State Examination, CDR=Clinical Dementia Rating*

Sample Size and Power

With power of 80% and α=0.05, a one-tailed test of the cross-sectional NTAD data should detect (i) medium, group-wise effect sizes of >0.5 and (ii) correlations with disease severity of >0.25. With 20% attrition in follow up and α=0.05, within-sample effect size >0.28 achieves power 80% (Figure 2).[34]

STUDY PROCEDURES

Overview of protocol stages

Screening

Potential participants are identified by electronically screening registry data from Join Dementia Research and regional memory clinics. Prospective participants are screened further on-site. After written, informed consent, a doctor administers the clinical interview and Haschinski ischemic scale. The remaining clinical assessments are completed by a member of the research team and include physiology and blood sampling. If amyloid status is unknown, participants proceed to either cerebrospinal fluid examination or amyloid PET imaging according to participant preference and eligibility. For patients, where participants' amyloid status has been confirmed at any timepoint previously, a positive result enables participation, a negative result excludes participation.

Stage 1: Baseline

One hundred people with mild cognitive impairment or Alzheimer's disease and 30 neurologically normal people proceed to baseline assessment. Participants undergo structured neuropsychological assessment, an M/EEG scan and MRI imaging of brain structure and function over two sessions (or three by preference).

Stage 1: Two-week M/EEG retest

We invite 30 people from the patient group (i.e. mild cognitive impairment or Alzheimer's disease) to repeat the M/EEG scan between two and four weeks after the first scan. Invitations are prioritised to people who can most readily attend the additional session (e.g. considering distance) until the target sample size is reached.

Stage 2: Annual follow-up 1

Participants in the patient group repeat the clinical and neuropsychological assessments, M/EEG scan, MRI scan and blood collection at 12 months after baseline.

Stage 2: Annual follow-up 2

Clinical and neuropsychological assessments are repeated at 24 months for participants in the patient group.

The timeline for the study is illustrated in Figure 3.

For peer review only

Blood Samples

Participants are asked to consume only water for two hours prior to blood collection. At baseline and follow-up, blood is drawn in the following order: 2.7ml in Sodium Citrate Tubes (plasma), 5ml in Serum Separator Tubes (serum), 10ml in an EDTA tube (DNA), 10ml in an EDTA tube (plasma and buffy coat) and 2.5ml in each of 2 PAXgene tubes (RNA). Once filled, the EDTA and PAXgene tubes are gently inverted 10 times. From April 2021, 4.9ml of blood is collected for SARS-CoV-2 serology. The samples are processed and frozen according to the guidelines for the constituents of interest in Table 2.

**Plasma**	The sample in the sodium citrate tube is centrifuged at 2000g for 10 minutes at room temperature. The plasma is aliquoted into 1.4 ml matrix tubes and stored at -80°C.
**Serum**	At least 30 minutes after blood collection, the sample in the serum separator tube is centrifuged at 2000g for 10 min at room temperature. The serum is aliquoted into 1.4ml matrix tubes and stored at -80°C.<br><br>Similarly, the sample in the S-monovette tube is centrifuged at 2000g for 10 minutes at room temperature at least 30 minutes after collection. The sample is aliquoted into 2ml Sarstedt tubes.
**DNA**	The first EDTA tube is frozen as soon as possible and stored at -80°C for later DNA extraction and analysis.
**Plasma and buffy coat**	The second EDTA tube is centrifuged immediately at 2500g for 15 minutes at room temperature. The plasma and buffy coat are aliquoted into separate 1.4 ml matrix tubes and stored at -80°C.
**RNA**	The Paxgene tubes are stored at room temperature for 2 hours before being frozen upright at -20°C for 24 hours and then stored at -80°C.

Table 2 Blood sample processing guidelines

Biomarkers for Alzheimer disease pathology

Clinical criteria are insufficient to reliably diagnose the presence of Alzheimer's disease pathology.[35,36] We seek additional biomarker evidence of the presence of Alzheimer's pathology, using either cerebrospinal fluid or PET imaging according to participant preference and eligibility.[37]

Cerebrospinal fluid

Cerebrospinal fluid is obtained by lumbar puncture and collected in polypropylene tubes. Within one hour of collection, the cerebrospinal fluid is centrifuged, separated and the supernatant frozen to −80°C for later batched analysis using a chemiluminescent enzyme immunoassay for total tau, phosphorylated tau and amyloid beta 1-42 levels. Positive amyloid status is indicated by a total tau to amyloid beta 1-42 ratio >1, and amyloid beta 1-42 concentration <450pg/ml.

Amyloid PET

Participants receive a 300 MBq bolus injection of florbetaben and are scanned 80-100 minutes post-injection on a GE Signa PET/MR scanner at the Wolfson Brain Imaging Centre,

only

Cambridge or a GE D710 PET/CT scanner at the Churchill Hospital, Oxford. The centiloid method is used to classify the florbetaben scans as amyloid positive with centiloid >1.19.[38,39]

Clinical Assessments

Clinical assessments are completed at a clinical research centre in one or two sessions.

Clinical Interview

A study clinician interviews the participant and study partner. The clinician follows a structured interview that covers socio-demographic factors, including age and years of education; lifestyle factors; family history of dementia; medical history, including information on significant medical conditions with date of onset; and concomitant medication usage, with details about dosage and duration.

Addenbrooke's Cognitive Examination

The revised Addenbrooke's Cognitive Examination (ACE-R) evaluates orientation, memory, verbal fluency, language and visuo-spatial domains.[40] It is administered and scored according to the ACE-R Administration and Scoring Guide (2006). Alternate versions at each visit reduce practice effects.

Mini-Mental State Examination

The Mini-Mental State Examination is acquired.[41]

Clinical Dementia Rating

The Clinical Dementia Rating quantifies the severity of dementia through a structured interview.[42–44] The interviewer rates participant impairments in 6 categories: memory, orientation, judgment and problem solving, community affairs, home and hobbies and personal care. The global CDR is derived from these ratings.[43]

Haschinski Ischaemic Score

The Rosen modification of Haschinski's Ischaemic Score seeks to differentiate between primary progressive dementias, including Alzheimer's disease and multi-infarct dementia.[45] A study clinician uses information from medical history, physical and neurological examination and medical records to determine the score. Scores below 4 indicate a low likelihood of vascular disease as the cause of dementia.

Self-reported questionnaires

The participant completes the 30-item Geriatric Depression Scale,[46] 40-item Spielberger State-Trait Anxiety Inventory,[47] and 11-item Pittsburgh Sleep Quality Index.[48] The study partner completes the 30-item Amsterdam Instrumental Activity of Daily Living Questionnaire (short version)[49] and Mild Behavioral Impairment Checklist.[50]

Physiological measures

Height, weight, hip-waist ratio, blood pressure, pulse rate and temperature are measured for each participant using a stadiometer, electronic weight scales and stretch-resistant tape.

Neuropsychological Assessment

The neuropsychological test battery closely resembles the Deep and Frequent Phenotyping study[51] and IMI-European Prevention of Alzheimer's Disease,[52,53] including the Pre-

For peer review only

Alzheimer Cognitive Composite.[54] The assessments take place in a private, testing room in one session with breaks as needed (see Table 3).

Order of assessments	Duration (minutes)
1. List learning (RBANS subtest)<br>2. Story memory (RBANS subtest)<br>3. Figure Copy (RBANS subtest)<br>4. Line orientation (RBANS subtest)<br>5. Picture naming (RBANS subtest)<br>6. Semantic fluency (RBANS subtest)<br>7. Digit span forwards (RBANS subtest)<br>8. Digit span backwards<br>9. Coding (RBANS subtest)<br>10. List recall (RBANS subtest)<br>11. List recognition (RBANS subtest)<br>12. Story recall (RBANS subtest)<br>13. Figure recall (RBANS subtest)	≈ 40
Break	≈ 15
14. Free and cued selective reminding test<br>15. Logical memory part 1<br>16. National adult reading test<br>17. Digit symbol substitution<br>18. Trails B<br>19. Logical memory part 2	≈ 45
Break	≈ 15
20. Reaction time task (CANTAB subtest)<br>21. Paired associates learning (CANTAB subtest)<br>22. Rapid visual processing (CANTAB subtest)<br>23. Spatial working memory (CANTAB subtest)<br>24. Four mountains task	≈ 30

Table 3 Order of neuropsychological assessments. *RBANS=Repeatable Battery for the Assessment of Neuropsychological Status, CANTAB = Cambridge Neuropsychological Test Automated Battery*

Repeatable Battery for the Assessment of Neuropsychological Status

Repeatable Battery for the Assessment of Neuropsychological Status (RBANS) measures cognitive decline in 5 domains: immediate memory, visuospatial, language, attention and delayed memory.[55] Participants receive alternate forms at repeated assessments. The immediate memory index comprises the *list learning subtest*, with immediate recall of 10-items over 4 trials and the *story memory subtest*, with immediate recall of a 12-item story over two trials. The visuospatial index comprises the *figure copy subtest*, which involves copying a geometric figure and the 10-item, *line orientation subtest*. The language index comprises the 10-item, *picture naming subtest* and the *semantic fluency subtest*, where the participant must name as many exemplars of the given semantic category as they can in 60 seconds. The attention index comprises the *digit span forwards subtest*, involving immediate

repetition of increasing digit strings and the *coding subtest*, scored as the total number of correctly coded numbers generated using an item-to-number code within 90 seconds. The delayed memory index comprises the *list recall subtest*, involving free recall of the list learning task; *list recognition*, where the participant decides whether a word was included in the list learning task; *story recall*, where the participant freely recalls the story memory task; and *figure recall*, where the participant draws from memory the figure presented in the figure copy task.

Digit Span Backwards

Digit span backwards, from the Wechsler adult intelligent scale,[56] is used in conjunction with RBANS digit span forwards. It follows the RBANS digit span format except that the participant is asked to immediately repeat in reverse order.

Free and Cued Selective Reminding Test

The Free and Cued Selective Reminding Test assesses episodic memory and distinguishes retrieval from storage deficits.[57] Sixteen pictured items are encoded during the initial learning phase, where the participant identifies and names items responding to unique semantic cues. After a short delay, the participant freely recalls all items. The interviewer prompts for each item not recalled using the unique semantic cues from the learning phase. Participants receive alternate forms at repeated assessments. This test forms part of the Pre-Alzheimer Cognitive Composite.

Logical Memory

Logical Memory, taken from the third edition of the Wechsler memory scale, assesses episodic memory.[58] The participant immediately recalls short stories they have been read. After a 30-minute delay with intervening tests, the participant freely recalls the stories and answers yes or no questions that test story recognition. This test forms part of the Pre-Alzheimer Cognitive Composite.

National Adult Reading Test

The national adult reading test (second edition) estimates premorbid intelligence,[59] from printed irregular words.

Digit Symbol Substitution

The digit symbol substitution from the Wechsler Adult Intelligence Scale assesses processing speed and attention.[56] The participant has 90 seconds to code as many correct symbols as possible corresponding to presented numbers by using the given number-to-symbol code. This test forms part of the Pre-Alzheimer Cognitive Composite.

Trails Making Test B

The Trails Making Test B assesses executive function, attention and processing speed.[60] Following a practice sample to ensure the participant understands the task, the participant is presented with the test comprising 25 encircled numbers which they must connect by alternating between numbers and letters. The time it takes to complete the sample and any errors made are recorded.

Cambridge Neuropsychological Test Automated Battery

The tablet-based Cambridge Neuropsychological Test Automated Battery test battery assesses processing speed, episodic memory, attention, working memory and executive

For peer review only

function.[61] The reaction time task (simple and five choice variant) assesses processing speed. The participant holds down a response button and must release this to respond to a target on screen. The paired associates learning task (standard variant) assesses episodic memory. The participant learns associations between patterns and their locations. There is an initial learning stage followed by immediate recall. The rapid visual processing task (3 target variant) assesses attention. Single digits appear on the screen and the participant responds when they see a string matching the target sequences. The spatial working memory task (standard variant) assesses working memory and executive function. The participant searches inside multiple boxes on the screen to find and collect tokens. Tokens do not appear in the same box twice.

Four Mountains Task

The tablet-based four mountains task assesses allocentric spatial processing.[62] During a learning phase of each trial, the participant learns the topographical layout of 4 mountains presented in a computer-generated landscape. Following a delay, the participant is presented with four alternative images and must identify the target image that matches the topographical mountain layout of the image presented in the learning phase of the trial, but with potentially altered colours, textures and points of view.

Neurophysiology (M/EEG)

M/EEG data are collected simultaneously in a magnetically-shielded room. At Cambridge, data are collected using the Elekta VectorView system from 2017 to December 2019, with 204 planar gradiometers and 102 magnetometers and a 70-channel Easycap. Stage 1 scans and the first 12 follow-up scans used the same scanner. The MEGIN Triux Neo M/EEG scanner is used from March 2020 onwards with the same sensor configuration as the VectorView and a 64-channel Easycap. At Oxford, the MEGIN Triux Neo M/EEG scanner and an EasyCap 60 channel BrainCap for MEG with an augmented 10/20 layout are used for all data collection. M/EEG data are collected at 1000Hz.

The position of the standard fiducial points, >300 additional head points, five head position indicator coils and the EEG electrodes are recorded using the Polhemus digitisation system. The head position indicator coils measure head position within the MEG helmet. Three pairs of bipolar electrodes record electrocardiogram data, with electrodes placed on the right clavicle and left lower rib, and vertical and horizontal electrooculogram data, with electrodes placed above the left eyebrow, below the left eye and lateral canthus of each eye. A reference electrode is placed on the left side of the nose and a ground electrode is placed on the left cheek. During the seated scan, the participant rests or performs simple task using a button box to respond. The participant wears non-magnetic earphones with sound delivered though plastic tubes and, if necessary, non-magnetic glasses. Prior to M/EEG a Snellen eye test and pure tone audiometry assess sight and hearing thresholds. The tasks are carried out in the following order for each M/EEG session. The study will identify a harmonised MEG/EEG pre-processing pipeline, to be confirmed before baseline data acquisition is completed.

Simple audio-visual task

The participant fixates on a red central fixation dot and responds each time an auditory or visual stimulus is presented (see Figure 4a). Auditory tones (n=100) of 300, 600 or 1200Hz frequency are presented for 300-ms duration after a blank interval of 1000ms. Visual stimuli are concentric black and white circles that appear for 300ms after 3000ms. Participants respond as quickly as possible after detecting a stimulus. Filler trials (n=30) containing only

For peer review only

the red fixation dot are also included. Visual and auditory trials are randomly interspersed. Ten initial practice trials are also included to familiarise participants to the task.

Auditory Mismatch-negativity Task

The roving auditory mismatch negativity task is designed to elicit error responses to novel, deviant tones followed by rapid plasticity as predictions are updated upon repetition of the deviant stimulus.[63,64] The participant passively watches a muted nature documentary. Through earpieces, they hear binaural, in-phase sinusoidal tones >60dB above the average auditory threshold with a duration of 100ms and stimulus onset asynchrony of 500ms. The frequency of each tone is the same within a block but different between the blocks. Blocks range from 400 to 800 Hz. The number of tones per blocks varies from 3 to 11, according to a truncated exponential distribution. The first tone of each block is called a deviant tone and the sixth repetition is called a standard tone (see Figure 4b).

Scene Repetition Task

In this passive memory test, participants view a series of complex scenes (landscapes and cityscapes) and press a button only when they see a scene containing a moon (n=26, different moon images). Target scenes containing a moon require participants to attend to every scene but are not of interest; the main interest is the difference between initial and repeated presentation of the non-target scenes. Each scene is presented for 800ms and preceded by a fixation cross presented for 200ms on average (100-300ms). Scenes are pseudo-randomly intermixed with the constraint that there are 10 initial ‘burn-in’ complex scenes with 2 moon target scenes, followed by 256 complex scenes presented twice with 14-93 (median=42) intervening scenes between the first and repeat presentation (see Figure 4c).

Cross-modal Oddball Task

The task assesses hippocampal-dependent paired associates learning.[65,66] The trials comprise a visual abstract “object” for 700ms and a sound presented for 400ms, starting 300ms after trial onset. The inter-stimulus interval averages 300ms. During the initial training period (80 trials), participants learn the association between four standard pairs of visual objects and sounds. The main task consists of 770 bimodal trials and 40 unimodal, target trials. The trials are randomly intermixed. The bimodal trials consist of: the standard, learnt pairs of objects and sounds (n=670); standard objects paired with a novel sound (n=50); and mismatched object-sound pairs, where the sound from a pre-learnt, standard pair is presented with an object from a different learnt, standard pair (n=50). For unimodal trials, the participant presses a button when they see a fifth visual object (the letter ‘a’, see Figure 4d) which ensures the task is attended. The task is followed by a 10-item assessment in which a standard sound is presented and the participant reports which of four presented objects the sound was paired with most often during the task.

Eyes-open Resting State

The participant is presented with a small central fixation cross and is given the following instructions: ‘In the next 5 minutes, we will do a recording as you rest. Please clear your mind, relax, and try not to think of anything in particular. Please stay awake and focus your eyes on the cross at the centre of the screen’.

Eyes-closed Resting State

The participant is given the following instructions: ‘In the next 5 min, we will do a recording as you rest. Please clear your mind, relax, and try not to think of anything in particular. Please close your eyes but do not fall asleep.’

For peer review only

Magnetic Resonance Imaging

The MRI sequences use 3T Siemens PRISMA scanners, at the MRC Cognition and Brain Science Unit, Cambridge, and the Oxford Centre for Human Brain Activity, Oxford. The sequences in order of acquisition are: localiser, T1-weighted, T2 Fluid Attenuated Inversion Recovery (FLAIR), T2*-weighted, T2-weighted with fat saturation, diffusion weighted imaging, quantitative susceptibility mapping, resting state eyes open, hippocampal subfields, and 3D arterial spin labelling. Details of the sequences are in Table 4.

**T1-weighted**	The 3D T1-weighted structural image is acquired using the Generalized Autocalibrating Partially Parallel Acquisitions (GRAPPA) technique applied to a high-resolution Magnetization Prepared Rapid Gradient Echo (MPRAGE) sequence with the following parameters: TE=2.91ms, TR = 2300.00ms, TI=900.00ms, flip angle =9 degrees, acquisition matrix =256 x 240, voxel size =1.00mm isotropic, number of slices =176, slice thickness =1mm, acquisition time =5 minutes and 12 seconds
**T2 FLAIR**	The 3D FLAIR structural image is acquired using the following parameters: TE=394.00ms, TR =5000.00ms, TI =1650.00ms, flip angle =120 degrees, acquisition matrix =256 x 256, voxel size =1.00mm isotropic, number of slices =192, slice thickness =1.00mm, acquisition time =5 minutes and 7 seconds
**T2*-weighted**	The T2*-weighted structural image is acquired using the following parameters: TE =20.00ms, TR =640.00ms, Flip angle =20 degrees, acquisition matrix =256 x 256, number of slices =47, slice thickness =3.00mm, Voxel size =0.90x0.90x3.00mm, Acquisition time =4 minutes and 7 seconds
**T2-weighted with fat saturation**	The T2-weighted image with fat saturation is acquired with the following parameters: TE =78.00ms, TR =4000.00ms, flip angle =150 degrees, acquisition matrix =256x232, number of slices =47, slice thickness=3.00mm, voxel size =0.90x0.90x3.00mm, acquisition time =3 minutes and 26 seconds
**Diffusion Weighted Imaging**	The Diffusion-Weighted Images are acquired with a spin-echo echo-planar sequence. B-values of 0, 300, 700 and 2000 s/mm^2 were used with 116 diffusion gradient directions obtained via MRTrix3.[67] The number of signal averages was 12, 8, 32, and 64 for b-values of 0, 300, 700 and 2000, respectively. The acquisition is run in the transverse orientation without any angulation. Other scan parameters include: TR=3800ms; TE=85.00ms, voxel size=2.50mm isotropic, acquisition matrix =96x96, number of slices=64, slice thickness =2.50mm, acquisition time=8 minutes and 45 seconds
**Quantitative Susceptibility Mapping**	The quantitative susceptibility mapping correlates with Aβ-amyloid iron density and is acquired with a 3D Gradient Echo sequence with multiple echoes and flow compensation in the readout and slice directions. (TE 1=5.20ms, TE 2=10.4ms, TE 3 =15.6ms, TE 4 =20.8ms, TE 5=26.0ms; TR =31.0ms, flip angle =15 degrees, acquisition matrix =256x192,

	number of slices =160 (per TE), slice thickness =1.00mm, voxel size =1.00mm isotropic, acquisition time =5 minutes 10 seconds.
**Resting state eyes open**	For the resting-state scan, the participant is presented with a fixation cross and asked to maintain fixation throughout the task. Echo planar images are acquired with 200 volumes with the following parameters: TE=30.00ms, TR=1500.00ms, flip angle =90 degrees, acquisition matrix =64x64, number of slices =54, slice thickness =3.00mm, voxel size = 3.00mm isotropic, acquisition time =5 minutes 50 seconds
**Hippocampal subfields**	The high-resolution, hippocampal subfield images are acquired with T2-weighted, turbo spin echo sequences and run with the following parameters: TE=50.00ms, TR=8020.00ms, flip angle =122 degrees, acquisition matrix =448x448, number of slices =30, slice thickness =2.00mm, voxel size =0.4x0.4x2.0mm, acquisition time =8 minutes 11 seconds.
**Arterial Spin Labelling**	3D Arterial Spin Labelling images are acquired using a flow-sensitive alternating inversion recovery spin-echo pulsed sequence with Q2-TIPS bolus saturation in the transverse plane with the following parameters: TR=4000.00ms; TE =13.20ms; flip angle =130 degrees, labelling duration =800ms, post-labelling delay =2000ms, acquisition matrix =64x60; slices per slab =32, slice thickness =4.50mm, voxel size =1.85x1.85x4.50mm, acquisition time =6 minutes 46 seconds.

Table 4 MRI sequence parameters. *TE=Echo time, TR=Repetition time, TI=Inversion time*

CURRENT STATUS

The study is currently active at both sites.

ETHICS AND DISSEMINATION PLAN

The study has received a favourable opinion from the East of England – Cambridge Central Research Ethics Committee (REC reference 18/EE/0042). Imaging data and clinical severity scores are hosted by the Dementias Platform UK Imaging Platform (https://portal.dementiasplatform.uk), using XNAT technology (https://www.xnat.org). The data will be made available by a managed access process through the Dementias Platform UK, subject to constraints to preserve confidentiality and privacy.

CONCLUSION

With a pressing need to evaluate new treatments for Alzheimer's disease, measures that are sensitive to changes in the underlying neurophysiology of the human disease are needed. The NTAD study establishes a dataset that will allow detailed assessment of neurophysiological measures of annual progression, linked to cognitive and MRI changes and baseline blood and DNA. The unique design of the NTAD study enables an assessment of MEG as a potential biomarker in Alzheimer's disease intervention trials.

For peer review only

REFERENCES

1 LaFerla FM, Green KN. Animal models of Alzheimer disease. *Cold Spring Harb Perspect Med* 2012;**2**:1–13. doi:10.1101/cshperspect.a006320

2 Drummond E, Wisniewski T. Alzheimer's disease: experimental models and reality. Acta Neuropathol. 2017;**133**:155–75. doi:10.1007/s00401-016-1662-x

3 Cummings JL, Morstorf T, Zhong K. Alzheimer's disease drug-development pipeline: Few candidates, frequent failures. *Alzheimer's Res Ther* 2014;**6**:1–7. doi:10.1186/alzrt269

4 Yiannopoulou KG, Anastasiou AI, Zachariou V, *et al.* Reasons for failed trials of disease-modifying treatments for alzheimer disease and their contribution in recent research. Biomedicines. 2019;**7**. doi:10.3390/biomedicines7040097

5 Anderson RM, Hadjichrysanthou C, Evans S, *et al.* Why do so many clinical trials of therapies for Alzheimer's disease fail? Lancet. 2017;**390**:2327–9. doi:10.1016/S0140-6736(17)32399-1

6 Mehta D, Jackson R, Paul G, *et al.* Why do trials for Alzheimer's disease drugs keep failing? *Expert Opin Investig Drugs* 2017;**26**:735–9. doi:10.1080/13543784.2017.1323868.Why

7 Frisoni GB, Fox NC, Jack CR, *et al.* The clinical use of structural MRI in Alzheimer disease. *Nat Rev Neurol* 2010;**6**:67–77. doi:10.1038/nrneurol.2009.215

8 Holland D, McEvoy LK, Dale AM. Unbiased comparison of sample size estimates from longitudinal structural measures in ADNI. *Hum Brain Mapp* 2012;**33**:2586–602. doi:10.1002/hbm.21386

9 Leung KK, Barnes J, Ridgway GR, *et al.* Automated cross-sectional and longitudinal hippocampal volume measurement in mild cognitive impairment and Alzheimer's disease. *Neuroimage* 2010;**51**:1345–59. doi:10.1016/j.neuroimage.2010.03.018

10 Cope TE, Rittman T, Borchert RJ, *et al.* Tau burden and the functional connectome in Alzheimer's disease and progressive supranuclear palsy. *Brain* 2018;**141**:550–67. doi:10.1093/brain/awx347

11 Passamonti L, Rodríguez PV, Hong YT, *et al.* PK11195 binding in Alzheimer disease and progressive supranuclear palsy. *Neurology* 2018;**90**:e1989–96. doi:10.1212/WNL.0000000000005610

12 Passamonti L, Rodríguez PV, Hong YT, *et al.* 18F-AV-1451 positron emission tomography in Alzheimer's disease and progressive supranuclear palsy. *Brain* 2017;**140**:781–91. doi:10.1093/brain/aww340

13 Leuzy A, Chiotis K, Lemoine L, *et al.* Tau PET imaging in neurodegenerative tauopathies—still a challenge. Mol. Psychiatry. 2019;**24**:1112–34. doi:10.1038/s41380-018-0342-8

14 Ossenkoppele R, Schonhaut DR, Schö M, *et al.* Tau PET patterns mirror clinical and neuroanatomical variability in Alzheimer's disease. *Brain* 2016;**139**:1551–67. doi:10.1093/brain/aww041

15 Cai L, Innis R, Pike V. Radioligand Development for PET Imaging of beta-Amyloid (AD)-Current Status. *Curr Med Chem* 2006;**14**:19–52. doi:10.2174/092986707779313471

For peer review only

16 Bevan-Jones WR, Surendranathan A, Passamonti L, *et al.* Neuroimaging of inflammation in memory and related other disorders (NIMROD) study protocol: A deep phenotyping cohort study of the role of brain inflammation in dementia, depression and other neurological illnesses. *BMJ Open* 2017;**7**. doi:10.1136/bmjopen-2016-013187

17 Koffie RM, Hyman BT, Spires-Jones TL. Alzheimer's disease: Synapses gone cold. Mol. Neurodegener. 2011;**6**:1–9. doi:10.1186/1750-1326-6-63

18 Palop JJ, Mucke L. Amyloid-B-induced neuronal dysfunction in Alzheimer's disease: From synapses toward neural networks. Nat. Neurosci. 2010;**13**:812–8. doi:10.1038/nn.2583

19 Kashyap G, Bapat D, Das D, *et al.* Synapse loss and progress of Alzheimer's disease - A network model. *Sci Rep* 2019;**9**:1–9. doi:10.1038/s41598-019-43076-y

20 De Wilde MC, Overk CR, Sijben JW, *et al.* Meta-analysis of synaptic pathology in Alzheimer's disease reveals selective molecular vesicular machinery vulnerability. *Alzheimer's Dement* 2016;**12**:633–44. doi:10.1016/j.jalz.2015.12.005

21 Henstridge CM, Sideris DI, Carroll E, *et al.* Synapse loss in the prefrontal cortex is associated with cognitive decline in amyotrophic lateral sclerosis. *Acta Neuropathol* 2018;**135**:213–26. doi:10.1007/s00401-017-1797-4

22 Menkes-Caspi N, Yamin HG, Kellner V, *et al.* Pathological tau disrupts ongoing network activity. *Neuron* 2015;**85**:959–66. doi:10.1016/j.neuron.2015.01.025

23 Pinotsis DA, Schwarzkopf DS, Litvak V, *et al.* Dynamic causal modelling of lateral interactions in the visual cortex. *Neuroimage* 2013;**66**:563–76. doi:10.1016/j.neuroimage.2012.10.078

24 Adams NE, Hughes LE, Phillips HN, *et al.* GABA-ergic dynamics in human frontotemporal networks confirmed by pharmaco-magnetoencephalography. *J Neurosci* 2020;:1689–19. doi:10.1523/jneurosci.1689-19.2019

25 Sami S, Williams N, Hughes LE, *et al.* Neurophysiological signatures of Alzheimer's disease and frontotemporal lobar degeneration: pathology versus phenotype. *Brain* 2018;**141**:2500–10. doi:10.1093/brain/awy180

26 Sitnikova TA, Hughes JW, Ahlfors SP, *et al.* Short timescale abnormalities in the states of spontaneous synchrony in the functional neural networks in Alzheimer's disease. *NeuroImage Clin* 2018;**20**:128–52. doi:10.1016/j.nicl.2018.05.028

27 Puttaert D, Coquelet N, Wens V, *et al.* Alterations in resting-state network dynamics along the Alzheimer's disease continuum. *Sci Rep* 2020;**10**:1–13. doi:10.1038/s41598-020-76201-3

28 Hughes LE, Henson RN, Pereda E, *et al.* Biomagnetic biomarkers for dementia: A pilot multicentre study with a recommended methodological framework for magnetoencephalography. *Alzheimer's Dement Diagnosis, Assess Dis Monit* 2019;**11**:450–62. doi:10.1016/j.dadm.2019.04.009

29 Hughes LE, Nestor PJ, Hodges JR, *et al.* Magnetoencephalography of frontotemporal dementia: Spatiotemporally localized changes during semantic decisions. *Brain* 2011;**134**:2513–22. doi:10.1093/brain/awr196

30 Hughes LE, Rittman T, Robbins TW, *et al.* Reorganization of cortical oscillatory dynamics underlying disinhibition in frontotemporal dementia. *Brain* 2018;**141**:2486–

For peer review only

99. doi:10.1093/brain/awy176

31 Hughes LE, Rittman T, Regenthal R, *et al.* Improving response inhibition systems in frontotemporal dementia with citalopram. *Brain* 2015;**138**:1961–75. doi:10.1093/brain/awv133

32 Hughes LE, Rowe JB. The impact of neurodegeneration on network connectivity: A study of change detection in frontotemporal dementia. *J Cogn Neurosci* 2013;**25**:802–13. doi:10.1162/jocn_a_00356

33 van Dellen E, de Waal H, van der Flier WM, *et al.* Loss of EEG Network Efficiency Is Related to Cognitive Impairment in Dementia With Lewy Bodies. *Mov Disord* 2015;**30**:1785–93. doi:10.1002/mds.26309

34 Erdfelder E, FAul F, Buchner A, *et al.* Statistical power analyses using G*Power 3.1: Tests for correlation and regression analyses. *Behav Res Methods* 2009;**41**:1149–60. doi:10.3758/BRM.41.4.1149

35 Ossenkoppele R, Jansen WJ, Rabinovici GD, *et al.* Prevalence of Amyloid PET Positivity in Dementia Syndromes: A Meta-analysis HHS Public Access. *JAMA* 2015;**313**:1939–49. doi:10.1001/jama.2015.4669

36 Monsell SE, Kukull WA, Roher AE, *et al.* Characterizing apolipoprotein E ε4 carriers and noncarriers with the clinical diagnosis of mild to moderate Alzheimer dementia and minimal β-amyloid peptide plaques. *JAMA Neurol* 2015;**72**:1124–31. doi:10.1001/jamaneurol.2015.1721

37 McKhann GM, Knopman DS, Chertkow H, *et al.* The diagnosis of dementia due to Alzheimer’s disease: Recommendations from the National Institute on Aging-Alzheimer’s Association workgroups on diagnostic guidelines for Alzheimer’s disease. *Alzheimer’s Dement* 2011;**7**:263–9. doi:10.1016/j.jalz.2011.03.005

38 Sabri O, Sabbagh MN, Seibyl J, *et al.* Florbetaben PET imaging to detect amyloid beta plaques in Alzheimer’s disease: Phase 3 study. *Alzheimer’s Dement* 2015;**11**:964–74. doi:10.1016/j.jalz.2015.02.004

39 Rowe CC, Doré V, Jones G, *et al.* 18F-Florbetaben PET beta-amyloid binding expressed in Centiloids. *Eur J Nucl Med Mol Imaging* 2017;**44**:2053–9. doi:10.1007/s00259-017-3749-6

40 Mioshi E, Dawson K, Mitchell J, *et al.* The Addenbrooke’s Cognitive Examination revised (ACE-R): A brief cognitive test battery for dementia screening. *Int J Geriatr Psychiatry* 2006;**21**:1078–85. doi:10.1002/gps.1610

41 Folstein MF, Folstein SE, McHugh PR. ‘Mini-mental state’. A practical method for grading the cognitive state of patients for the clinician. *J Psychiatr Res* 1975;**12**:189–98. doi:10.1016/0022-3956(75)90026-6

42 Hughes CP, Berg L, Danziger WL, *et al.* A new clinical scale for the staging of dementia. *Br J Psychiatry* 1982;**140**:566–72. doi:10.1192/bjp.140.6.566

43 Morris JC. The clinical dementia rating (cdr): Current version and scoring rules. *Neurology* 1993;**43**:2412–4. doi:10.1212/wnl.43.11.2412-a

44 Washington University Alzheimer’s Disease Research Center. The CDR® Dementia Staging Instrument. SCALE. 1999;:1. doi:20 May 1999

45 Rosen WG, Terry RD, Fuld PA, *et al.* Pathological verification of ischemic score in differentiation of dementias. *Ann Neurol* 1980;**7**:486–8. doi:10.1002/ana.410070516

For peer review only

46 Ishihara T, Terada S. [Geriatric Depression Scale (GDS)]. *Nihon Rinsho* 2011;**69 Suppl 8**:455–8. doi:10.1300/j018v05n01_09

47 Spielberger CD, Gorsuch RL, Lushene RE. Manual for the State-Trait Anxiety Inventory. 1970.

48 Buysse DJ, Reynolds CF, Monk TH, *et al.* The Pittsburgh sleep quality index: A new instrument for psychiatric practice and research. *Psychiatry Res* 1989;**28**:193–213. doi:10.1016/0165-1781(89)90047-4

49 Jutten RJ, Peeters CFW, Leijdesdorff SMJ, *et al.* Detecting functional decline from normal aging to dementia: Development and validation of a short version of the Amsterdam IADL Questionnaire. *Alzheimer's Dement Diagnosis, Assess Dis Monit* 2017;**8**:26–35. doi:10.1016/j.dadm.2017.03.002

50 Ismail Z, Agüera-Ortiz L, Brodaty H, *et al.* The Mild Behavioral Impairment Checklist (MBI-C): A Rating Scale for Neuropsychiatric Symptoms in Pre-Dementia Populations. *J Alzheimer's Dis* 2017;**56**:929–38. doi:10.3233/JAD-160979

51 Koychev I, Lawson J, Chessell T, *et al.* Deep and Frequent Phenotyping study protocol: an observational study in prodromal Alzheimer's disease. *BMJ Open* 2019;**9**:24498. doi:10.1136/bmjopen-2018-024498

52 Solomon A, Kivipelto M, Molinuevo JL, *et al.* European Prevention of Alzheimer's Dementia Longitudinal Cohort Study (EPAD LCS): Study protocol. *BMJ Open* 2018;**8**. doi:10.1136/bmjopen-2017-021017

53 Ritchie K, Ropacki M, Albala B, *et al.* Recommended cognitive outcomes in preclinical Alzheimer's disease: Consensus statement from the European Prevention of Alzheimer's Dementia project. Alzheimer's Dement. 2017;**13**:186–95. doi:10.1016/j.jalz.2016.07.154

54 Donohue MC, Sperling RA, Salmon DP, *et al.* The preclinical Alzheimer cognitive composite: Measuring amyloid-related decline. *JAMA Neurol* 2014;**71**:961–70. doi:10.1001/jamaneurol.2014.803

55 Randolph C, Tierney MC, Mohr E, *et al.* The Repeatable Battery for the Assessment of Neuropsychological Status (RBANS): Preliminary clinical validity. *J Clin Exp Neuropsychol* 1998;**20**:310–9. doi:10.1076/jcen.20.3.310.823

56 Wechsler D. *Wechsler Adult Intelligence Scale - Revised*. San Antonio, TX: Psychological Corporation 1981.

57 Grober E, Veroff AE, Lipton RB. Temporal unfolding of declining episodic memory on the Free and Cued Selective Reminding Test in the predementia phase of Alzheimer's disease: Implications for clinical trials. *Alzheimer's Dement Diagnosis, Assess Dis Monit* 2018;**10**:161–71. doi:10.1016/j.dadm.2017.12.004

58 Wechsler D. *Wechsler Memory Scale - Third UK Edition.* London: Harcourt Assessment 1999.

59 Nelson HE, Willison JR. *National Adult Reading Test (NART): Test Manual (2nd edn).* 1991.

60 Bowie CR, Harvey PD. Administration and interpretation of the Trail Making Test. *Nat Protoc* 2006;**1**:2277–81. doi:10.1038/nprot.2006.390

61 Cambridge Cognition. CANTAB® [Cognitive assessment software]. 2019.

For peer review only

62 Hartley T, Bird CM, Chan D, *et al.* The hippocampus is required for short-term topographical memory in humans. *Hippocampus* 2007;**17**:34–8. doi:10.1002/hipo.20240

63 Kiebel SJ, Garrido MI, Friston KJ. Dynamic causal modelling of evoked responses: The role of intrinsic connections. *Neuroimage* 2007;**36**:332–45. doi:10.1016/j.neuroimage.2007.02.046

64 Friston K. A theory of cortical responses. *Philos Trans R Soc B Biol Sci* 2005;**360**:815–36. doi:10.1098/rstb.2005.1622

65 De Rover M, Pironti VA, McCabe JA, *et al.* Hippocampal dysfunction in patients with mild cognitive impairment: A functional neuroimaging study of a visuospatial paired associates learning task. *Neuropsychologia* 2011;**49**:2060–70. doi:10.1016/j.neuropsychologia.2011.03.037

66 Blackwell AD, Sahakian BJ, Vesey R, *et al.* Detecting dementia: Novel neuropsychological markers of preclinical Alzheimer's disease. *Dement Geriatr Cogn Disord* 2004;**17**:42–8. doi:10.1159/000074081

67 Tournier JD, Smith R, Raffelt D, *et al.* MRtrix3: A fast, flexible and open software framework for medical image processing and visualisation. Neuroimage. 2019;**202**. doi:10.1016/j.neuroimage.2019.116137

68 American Psychiatric Association. *Diagnostic and Statistical Manual of Mental Disorders*. Fifth. American Psychiatric Association 2013. doi:10.1176/appi.books.9780890425596

For peer review only

FOOTNOTES

Author statement: JHL and JBR drafted the manuscript, with review and contribution from all authors. JBR is the chief investigator of the study. RNH, ACN, MW, KDS and VR are principal investigators, contributing to conception and design. EK led protocol development and governance. JBR, MW, ACN, RNH, JI, SL, MP and KDS conceived and designed the study.

Patient and public involvement

The use and tolerability of M/EEG in combination with the PET or CSF, MRI and neuropsychology was discussed as part of Alzheimer Society sponsored patient groups with patients and carers affected by AD/MCI for the closely related Dementias Platform UK study known as 'Deep and frequent phenotyping', with which NTAD is aligned in design and methods. In addition, the use of MEG for dementia research featured in the public engagement event 'MEG and me' as part of the Cambridge Science Festival and has been discussed at open meetings at the Cambridge Science Festival and Alzheimer Research UK Eastern Region network public meetings. The study is also co-funded by Alzheimer Research UK, a charity with a joint patient advocacy and research funding remit.

Funding: This work is part of the Dementias Platform UK which is funded by Medical Research Council (MR/L023784/1 & MR/L023784/2), Janssen, AstraZeneca, Araclon, IXICO, Somalogic, GlaxoSmithKline, Invicro, Cambridge Cognition and Cognetivity. The study has additional support from Alzheimer's Research UK (ARUK-PG2017B-19), the Wellcome Trust (103838), Medical Research Council (SUAG/051 G101400; SUAG/046 G101400), NIHR Cambridge Biomedical Research Centre (BRC-1215-20014) and NIHR Oxford Biomedical Research Centre (BRC-1215-20008). The views expressed are those of the authors and not necessarily those of the NIHR or the Department of Health and Social Care. For the purpose of Open Access, the author has applied a CC BY public copyright licence to any Author Accepted Manuscript version arising from this submission.

Conflicts of interests: John Isaac is employed by Johnson & Johnson and may currently hold Johnson & Johnson stocks. Michael Perkinton is employed by AstraZeneca and may currently hold AstraZeneca stocks or stock options. Yun-Ju Cheng and Stephen Lowe are employed by Eli Lilly and may currently hold Eli Lilly stock. Mark Woolrich receives royalties from FSL and is senior editor for Neuroimage. James Rowe is Chief Scientific Adviser for Alzheimer Research UK and is a trustee of Brain, PSP association and Darwin college.

For peer review only

For peer review only - http://bmjopen.bmj.com/site/about/guidelines.xhtml

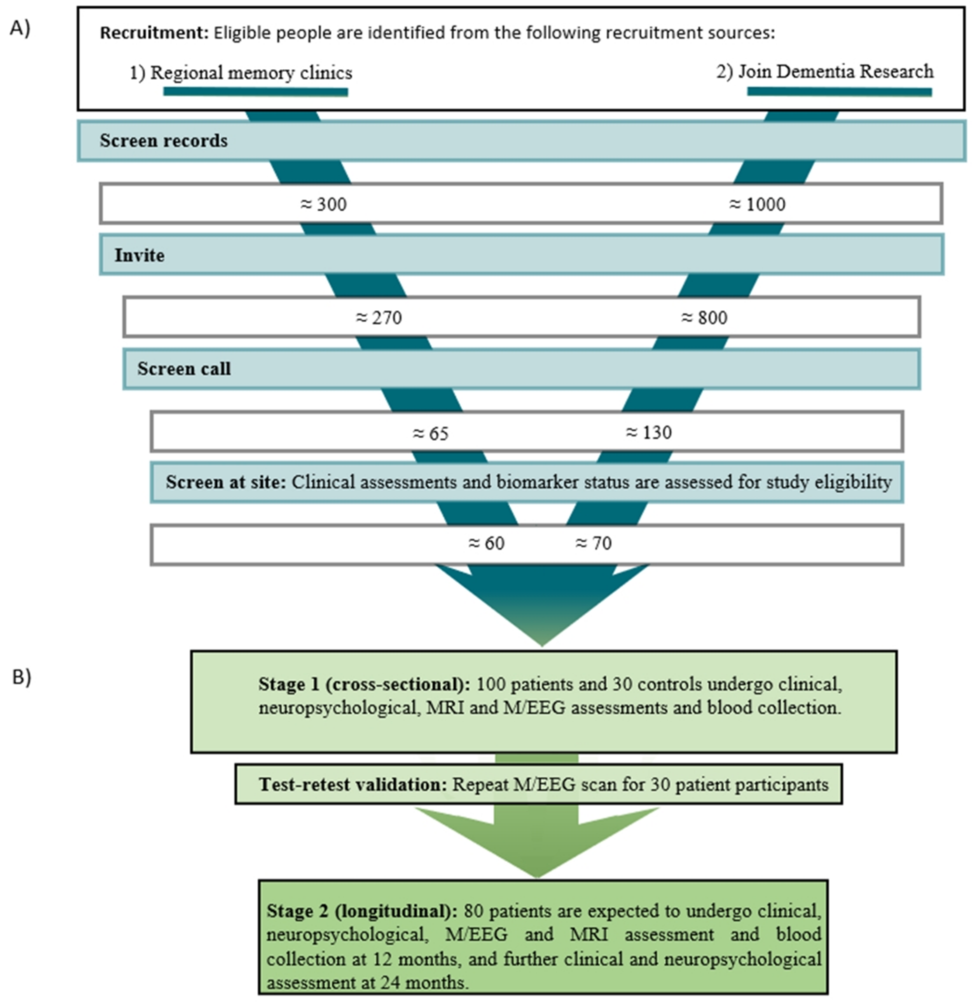

Figure 1 Participant flow chart. A) Recruitment strategy: Electronic screening of records from regional memory clinics and Join Dementia Research generates approximately 300 potential patient and 1000 potential patient and control participants who are invited to telephone screening. People who remain eligible are screened further on-site to identify 100 patient and 30 control participants. B) Study stages: Stage 1 consists of the cross-sectional baseline assessments and, for 30 patients, a repeat of the M/EEG assessment two weeks after the first. Only patients continue to the longitudinal stage 2 of the study, with repeat 12 and 24 months after baseline. Attrition of 20% is expected at stage 2. M/EEG=Magnetoencephalography combined with electroencephalography imaging, MRI=Magnetic resonance imaging

600x644mm (236 x 236 DPI)

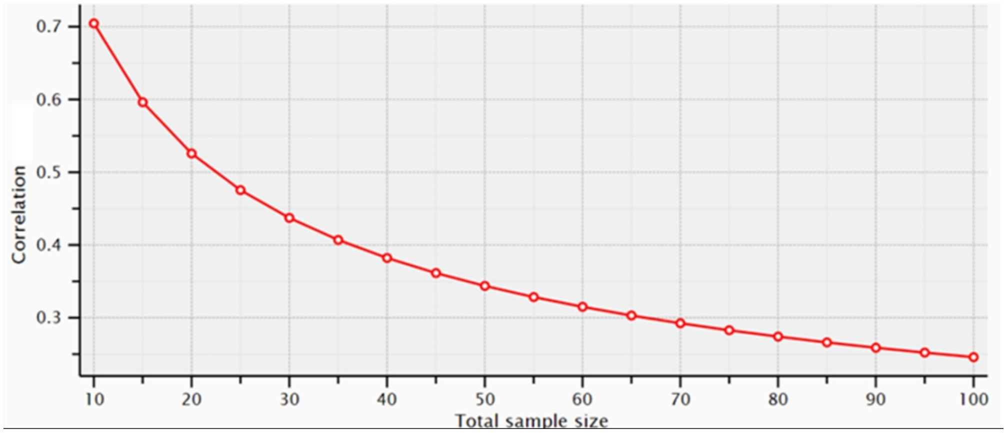

Figure 2 Effect sizes required to detect longitudinal correlations with disease severity at different attrition rates (resulting in reduced longitudinal sample size) with 80% power and α=0.05. We expect <20% attrition.

679x292mm (47 x 47 DPI)

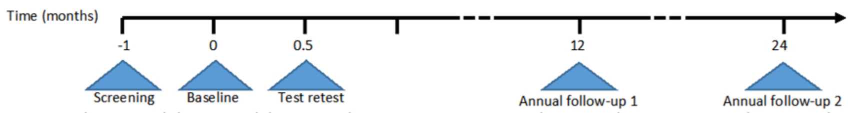

	Screening	Baseline	Test retest	Annual follow-up 1	Annual follow-up 2
Blood collection	X			X	
Biomarker samples	X				
Clinical assessments	X			X	X
Neuropsychological assessments		X		X	X
EEG and MEG scan		X	X	X	
MRI scan		X		X	

Figure 3 Timeline summary of study procedures

729x352mm (38 x 38 DPI)

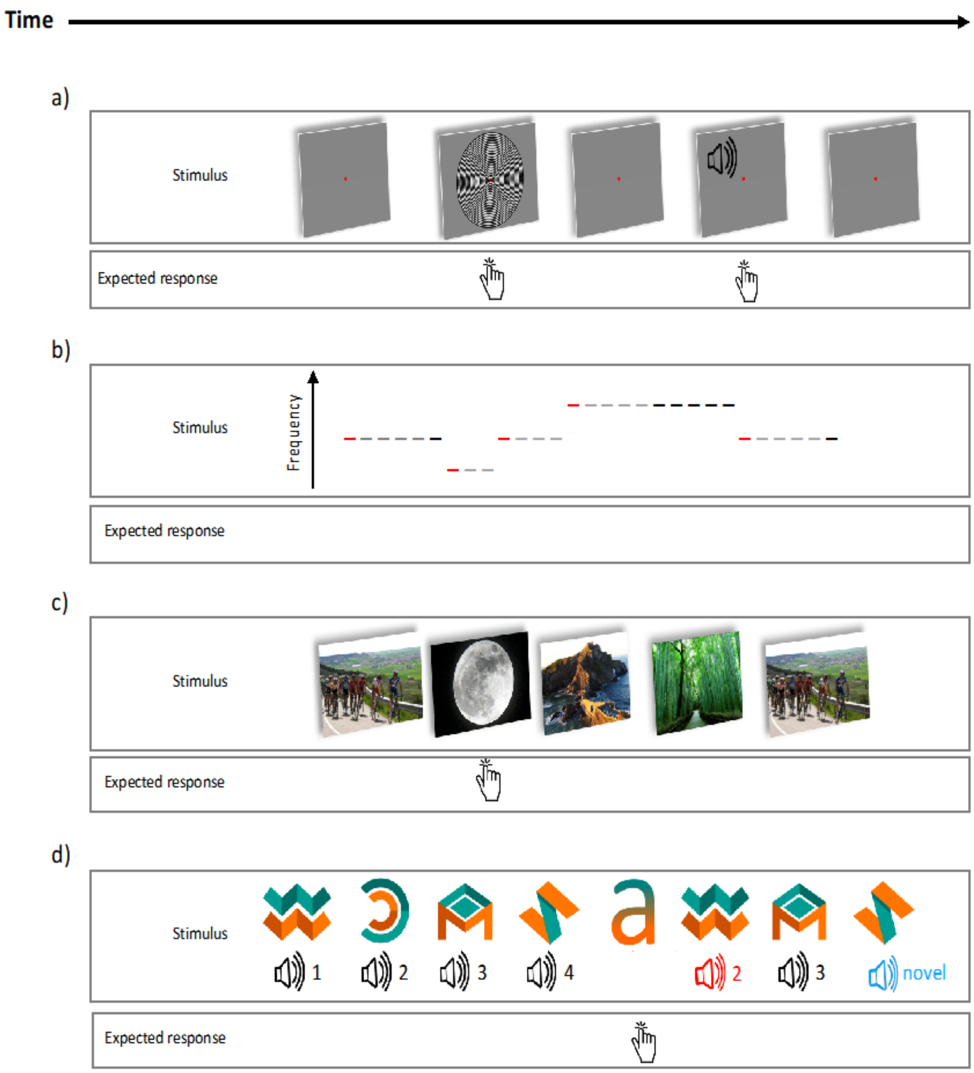

Figure 4 a) Audio-visual task: Participants visually fixate on a red dot and press a button whenever they hear or see something. (b) Mismatch-negativity task: Participants passively watch a nature documentary while listening to tones through the earpieces. Red dashes represent deviant tones and black dashes represent standard tones (from the sixth repetition). (c) Scene repetition task: Participants view scenes and press the button when they see a scene containing a moon. Each non-target scene repeats once during the task. (d) Cross-modal oddball task: Participants view object-tone pairs consisting of 4 learnt (standard) pairs (the standard sounds are black in the figure); associative-deviant, object-tone pairs (the associative-deviant sound is coloured red in the figure); and standard objects presented with novel tones (the novel-deviant tones are blue in the figure). The participant presses a button when they see the letter ‘a’.

577x632mm (38 x 38 DPI)

STROBE Statement—Checklist of items that should be included in reports of ***cohort studies***

NB result and discussion sections are less relevant as this is for an observational protocol paper

	Item No	Recommendation	Page No
**Title and abstract**	1	(*a*) Indicate the study's design with a commonly used term in the title or the abstract<br>(*b*) Provide in the abstract an informative and balanced summary of what was done and what was found	1<br>2
**Introduction**			
Background/rationale	2	Explain the scientific background and rationale for the investigation being reported	3
Objectives	3	State specific objectives, including any prespecified hypotheses	4
**Methods**			
Study design	4	Present key elements of study design early in the paper	5
Setting	5	Describe the setting, locations, and relevant dates, including periods of recruitment, exposure, follow-up, and data collection	7
Participants	6	(*a*) Give the eligibility criteria, and the sources and methods of selection of participants. Describe methods of follow-up<br>(*b*) For matched studies, give matching criteria and number of exposed and unexposed	9-15
Variables	7	Clearly define all outcomes, exposures, predictors, potential confounders, and effect modifiers. Give diagnostic criteria, if applicable	8-15
Data sources/ measurement	8*	For each variable of interest, give sources of data and details of methods of assessment (measurement). Describe comparability of assessment methods if there is more than one group	NA
Bias	9	Describe any efforts to address potential sources of bias	NA
Study size	10	Explain how the study size was arrived at	7
Quantitative variables	11	Explain how quantitative variables were handled in the analyses. If applicable, describe which groupings were chosen and why	4
Statistical methods	12	(*a*) Describe all statistical methods, including those used to control for confounding<br>(*b*) Describe any methods used to examine subgroups and interactions<br>(*c*) Explain how missing data were addressed<br>(*d*) If applicable, explain how loss to follow-up was addressed<br>(*e*) Describe any sensitivity analyses	4
**Results**			
Participants	13*	(a) Report numbers of individuals at each stage of study—eg numbers potentially eligible, examined for eligibility, confirmed eligible, included in the study, completing follow-up, and analysed<br>(b) Give reasons for non-participation at each stage<br>(c) Consider use of a flow diagram	Fig 1
Descriptive data	14*	(a) Give characteristics of study participants (eg demographic, clinical, social) and information on exposures and potential confounders<br>(b) Indicate number of participants with missing data for each variable of interest<br>(c) Summarise follow-up time (eg, average and total amount)	NA
Outcome data	15*	Report numbers of outcome events or summary measures over time	NA

For peer review only - http://bmjopen.bmj.com/site/about/guidelines.xhtml

Main results	16	(*a*) Give unadjusted estimates and, if applicable, confounder-adjusted estimates and their precision (eg, 95% confidence interval). Make clear which confounders were adjusted for and why they were included<br>(*b*) Report category boundaries when continuous variables were categorized<br>(*c*) If relevant, consider translating estimates of relative risk into absolute risk for a meaningful time period	NA
Other analyses	17	Report other analyses done—eg analyses of subgroups and interactions, and sensitivity analyses	NA
**Discussion**			
Key results	18	Summarise key results with reference to study objectives	NA
Limitations	19	Discuss limitations of the study, taking into account sources of potential bias or imprecision. Discuss both direction and magnitude of any potential bias	NA
Interpretation	20	Give a cautious overall interpretation of results considering objectives, limitations, multiplicity of analyses, results from similar studies, and other relevant evidence	NA
Generalisability	21	Discuss the generalisability (external validity) of the study results	NA
**Other information**			
Funding	22	Give the source of funding and the role of the funders for the present study and, if applicable, for the original study on which the present article is based	21

*Give information separately for exposed and unexposed groups.

Note: An Explanation and Elaboration article discusses each checklist item and gives methodological background and published examples of transparent reporting. The STROBE checklist is best used in conjunction with this article (freely available on the Web sites of PLoS Medicine at http://www.plosmedicine.org/, Annals of Internal Medicine at http://www.annals.org/, and Epidemiology at http://www.epidem.com/). Information on the STROBE Initiative is available at http://www.strobe-statement.org.

For peer review only

For peer review only - http://bmjopen.bmj.com/site/about/guidelines.xhtml

The New Therapeutics in Alzheimer's Disease Longitudinal Cohort study (NTAD): study protocol

Journal:	*BMJ Open*
Manuscript ID	bmjopen-2021-055135.R1
Article Type:	Protocol
Date Submitted by the Author:	24-Dec-2021

Complete List of Authors:	Lanskey, Juliette; University of Cambridge, MRC Cognition and Brain Sciences Unit<br>Kocagoncu, Ece; University of Cambridge, Department of Clinical Neurosciences and Cambridge University Hospitals NHS Foundation Trust, Cambridge Biomedical Campus<br>Quinn, Andrew; University of Oxford, Oxford Centre for Human Brain Activity, Wellcome Centre for Integrative Neuroimaging, Department of Psychiatry<br>Cheng, Yun-Ju; Lilly Corporate Center<br>Karadag, Melek; University of Cambridge, Department of Clinical Neurosciences and Cambridge University Hospitals NHS Foundation Trust, Cambridge Biomedical Campus<br>Pitt, Jemma; University of Oxford, Oxford Centre for Human Brain Activity, Wellcome Centre for Integrative Neuroimaging, Department of Psychiatry<br>Isaac, John; Johnson and Johnson London Innovation Center, Neuroscience External Innovation<br>Lowe, Stephen; Lilly Corporate Center<br>Perkinton, Michael; AstraZeneca, Neuroscience, BioPharmaceuticals R&D<br>Raymont, Vanessa; University of Oxford, Department of Psychiatry<br>Singh, Krish; Cardiff University, Cardiff University Brain Research Imaging Centre, School of Psychology<br>Woolrich, Mark; University of Oxford, Oxford Centre for Human Brain Activity, Wellcome Centre for Integrative Neuroimaging, Department of Psychiatry<br>Nobre, Anna; University of Oxford, Oxford Centre for Human Brain Activity, Wellcome Centre for Integrative Neuroimaging, Department of Psychiatry<br>Henson, Richard; University of Cambridge, MRC Cognition and Brain Sciences Unit; University of Cambridge, Department of Psychiatry<br>Rowe, James; University of Cambridge, Department of Clinical Neurosciences and Cambridge University Hospitals NHS Foundation Trust, Cambridge Biomedical Campus; University of Cambridge, MRC Cognition and Brain Sciences Unit

**Primary Subject Heading**:	Neurology
Secondary Subject Heading:	Radiology and imaging
Keywords:	Protocols & guidelines < HEALTH SERVICES ADMINISTRATION &

	MANAGEMENT, Dementia < NEUROLOGY, Neurophysiology < NEUROLOGY, NEUROPHYSIOLOGY, NEUROLOGY, RADIOLOGY & IMAGING

SCHOLARONE™
Manuscripts

The New Therapeutics in Alzheimer's Disease Longitudinal Cohort study (NTAD): study protocol

Juliette H Lanskey[1], Ece Kocagoncu[2], Andrew J Quinn[3], Yun-Ju Cheng[4], Melek Karadag[2], Jemma Pitt[3], John Isaac[5], Stephen Lowe[4], Michael Perkinton[6], Vanessa Raymont[7], Krish D Singh[8], Mark Woolrich[3], Anna C Nobre[3], Richard N Henson[1,9], James B Rowe[1,2] on behalf of the NTAD study group

Affiliations

1. MRC Cognition and Brain Sciences Unit, University of Cambridge, Cambridge, UK
2. Department of Clinical Neurosciences and Cambridge University Hospitals NHS Foundation Trust, Cambridge Biomedical Campus, Cambridge, UK
3. Oxford Centre for Human Brain Activity, Wellcome Centre for Integrative Neuroimaging, Department of Psychiatry, University of Oxford, Oxford, UK
4. Lilly Corporate Center, Indianapolis, USA
5. Neuroscience External Innovation, Johnson & Johnson Innovations, London, UK
6. Neuroscience, BioPharmaceuticals R&D, AstraZeneca, Cambridge, UK
7. Department of Psychiatry, University of Oxford, Oxford, UK
8. Cardiff University Brain Research Imaging Centre, School of Psychology, Cardiff University, Cardiff, UK
9. Department of Psychiatry, University of Cambridge, Cambridge, UK

Correspondence

Juliette Lanskey
MRC Cognition and Brain Sciences Unit,
15 Chaucer Road,
Cambridge, CB2 7EF, UK
Juliette.lanskey@mrc-cbu.cam.ac.uk

Words	3961
Figures	4
Tables	4

For peer review only

ABSTRACT

Introduction

With the pressing need to develop treatments that slow or stop the progression of Alzheimer's disease, new tools are needed to reduce clinical trial duration and validate new targets for human therapeutics. Such tools could be derived from neurophysiological measurements of disease.

Methods and Analysis

The New Therapeutics in Alzheimer's disease study (NTAD) aims to identify a biomarker set from magneto/electro-encephalography that is sensitive to disease and progression over one year. The study will recruit 100 people with amyloid-positive mild cognitive impairment or early-stage Alzheimer's disease and 30 healthy controls aged between 50 and 85 years. Repeat measurements of the clinical, cognitive and imaging data (magnetoencephalography, electroencephalography and magnetic resonance imaging) of participants with Alzheimer's disease or mild cognitive impairment will be taken at baseline and at one year. To assess reliability of magneto/electro-encephalographic changes, a subset of 30 participants with mild cognitive impairment or early-stage Alzheimer's disease will undergo repeat magneto/electro-encephalography two weeks after baseline. Clinical and cognitive assessment will be repeated at 2 years. Baseline and longitudinal changes in neurophysiology are the primary analyses of interest. Additional outputs will include atrophy and cognitive change and the respective numbers needed to treat each arm of simulated clinical trials of a future disease modifying therapy.

Ethics and Data statement

The study has received a favourable opinion from the East of England Cambridge Central Research Ethics Committee (REC reference 18/EE/0042). Results will be disseminated through internal reports, peer-reviewed scientific journals, conference presentations, website publication, submission to regulatory authorities and other publications. Data will be made available via the Dementias Platform UK Data Portal on completion of initial analyses by the NTAD study group.

For peer review only

STRENGTHS AND LIMITATIONS OF THIS STUDY

Strengths:

- NTAD is a longitudinal, multicentre study of magneto/electro-encephalographic measures of Alzheimer's disease progression
- NTAD assesses the test-retest reliability of magneto/electro-encephalographic parameters
- All participants with early-stage Alzheimer's disease or mild cognitive impairment will be amyloid positive

Limitations:

- Attrition during follow up may limit inferences
- Recruitment is from volunteer panels and clinical services that may not reflect national diversity

For peer review only

INTRODUCTION

With 44 million people living worldwide with dementia, treatments to slow or stop disease progression are urgently needed. Alzheimer's disease is the most prevalent dementia but despite rapid advances in therapeutics within preclinical models,[1,2] clinical trials remain expensive, slow and challenging[3–5] with high-profile failures.[6] Significant bottlenecks exist in early-phase trials, where efficacy rates are low and costs rapidly escalate. To bridge the gap between animal models and the human disease, better tools are needed to quantify pathogenic and pathophysiological mechanisms in patients, *in vivo* providing evidence to pursue or discontinue trials of a candidate treatment. These tools should be safe, scalable and able to support early-phase trials over a short duration and viable budget.

Brain imaging is widely used to diagnose dementia and measure pathology in clinical trials. There are diverse imaging methods with differential sensitivity to brain structure, chemistry, pathology and function. For example, magnetic resonance imaging (MRI) is commonly used to measure structural changes related to Alzheimer's disease,[7] such as entorhinal cortex[8] and hippocampus volumes.[9] Positron emission tomography (PET) can quantify and localise Alzheimer pathology through the use of ligands that bind to the aggregated tau protein,[10–14] beta-amyloid plaques[15] and neuroinflammation.[16]

Loss of synapses and synaptic plasticity is an early feature of Alzheimer's disease.[17–19] Indeed, cognitive decline may be more directly related to loss of synapses or synaptic plasticity than to atrophy.[20,21] As synaptic changes occur early in Alzheimer's disease,[20] synaptic measures could be sensitive to the earliest precursors of cognitive involvement. This accords with preclinical models in transgenic mice where network physiology and cognition are impaired before tangles or cell death.[22]

In contrast to preclinical models and post-mortem analysis, there are limited options to assess synaptic function in humans, *in vivo*. While PET imaging now offers ligands that indirectly quantify synaptic density, electroencephalography (EEG) and magnetoencephalography (MEG) measure neurophysiological properties dependent on synaptic integrity and function. MEG can identify synaptic and local circuit impairments[23,24] and their impact on network dynamics in Alzheimer's Disease,[25–28] frontotemporal dementia,[24,29–32] and Lewy-body disease.[33] MEG and EEG therefore have potential to support and de-risk clinical trials of novel compounds. To enable MEG as a viable biomarker in clinical trials, one must assess longitudinal MEG and test-retest reliability.

The New Therapeutics for Alzheimer's Disease (NTAD) study aims to identify viable MEG and EEG biomarkers for clinical trials. NTAD is a multicentre study established by the Dementias Platform UK and supported by the Medical Research Council, Alzheimer's Research UK and industry partners.

This paper describes the NTAD protocol to acquire longitudinal MEG and EEG data in people with biomarker-positive mild cognitive impairment or early Alzheimer's disease.

Research Aims

The primary objective is to identify a biomarker set from neurophysiological MEG and EEG (M/EEG) sensitive to the presence and progression of Alzheimer's disease. We aim to harmonise M/EEG protocols across sites to allow biomarker identification from pooled data. Such a neurophysiological biomarker, or biomarker set, should be related to cognitive function, have high test-retest reliability and track disease progression over the duration of clinical trials. Sensitivity to disease progression should ideally outperform current widely used biomarkers such as MRI and cognitive tests.

Our secondary objective is to identify a biomarker set that can predict disease progression, explain individual differences in the future disease trajectory and variation in cognitive decline over time.

Analysis

Investigators across sites will identify harmonised M/EEG pre-processing pipelines to reduce analytical variance. Following standardised pre-processing, investigators will address the study aims with multiple analytical approaches. Investigators are encouraged to pre-register specific analyses. Analyses will follow an overarching framework:

Stage 0 (descriptive) analysis will describe demographics; APOE genotype; blood-based, fluidic biomarkers; neurocognitive profiles and regional brain atrophy. This serves to confirm the expected patterns of cognitive decline and atrophy typical of people with mild cognitive impairment and early Alzheimer's disease and to enable comparison of the NTAD cohort across sites and with other study populations.

Stage 1 (baseline) analyses will identify reliable and sensitive baseline, disease-related M/EEG parameters in terms of (i) the sensitivity of cross-sectional M/EEG parameters to group differences, (ii) the correlation of cross-sectional M/EEG parameters to baseline cognition, atrophy and fluidic markers and (iii) the test-retest reliability of M/EEG parameters.

Stage 2 (longitudinal) analyses will assess the sensitivity of selected neurophysiological markers to longitudinal change by assessing how they change over ~12 months. We shall compare their sensitivity (as accuracy and effect sizes) to disease progression with MRI and cognitive measures. Mediation modelling will determine how longitudinal changes in brain structure and neurophysiology explain cognitive changes. Where data are pooled across sites, site location will be included as a model factor.

Stage 3 (predictive) analyses aim to identify baseline parameters that predict disease progression and identify the optimal parameter, or parameter set, that provide parsimonious or unique predictive information.

Parameters at stages 1-3 include: (i) summary statistics of evoked and induced responses for each task; (ii) summary statistics of the spectral density in resting-state recordings; (iii) data-

For peer review only

driven connectivity metrics, including those derived from Hidden Markov modelling;[34–36] and (iv) parameters of model-based analyses of evoked responses and spectral densities using dynamic causal modelling.[24,37,38]

DNA will allow determination of genotype, including common genetic variants; and post-hoc identification of rare, autosomal dominant cases which may have distinctive physiology and phenotype. *Post-hoc* exploratory analyses will examine the relationship between cognitive and imaging biomarkers as a function of (i) variations in tau haplotype, (ii) polymorphisms of genes related to plasticity, (iii) Alzheimer's disease risk alleles (e.g. apolipoprotein e4), (iv) polygenic risk scores of Alzheimer's disease and (v) plasma tau levels. RNA is collected to enable post-hoc transcriptomics. These are exploratory genetic options, whereas participation and the principal analyses are not genetically determined.

With data sharing, new analytical methods may be applied to this dataset alone, or in combination with other cohorts' data, e.g. ENIGMA-MEG (http://enigma.ini.usc.edu/ongoing/enigma-meg-working-group/) and BIOFIND (https://biofind-archive.mrc-cbu.cam.ac.uk/).

METHODS

Study Design

This is a repeated-measures, observational-design study with 130 participants tested at two sites: Cambridge University and Oxford University, each in partnership with local NHS Hospital Trusts. The planned start and end dates are 01/06/2018 and 01/07/2023. The protocol includes two stages (Figure 1). The first, cross-sectional stage consists of baseline and test-retest sessions. Baseline sessions are comprised of clinical and neuropsychological assessments, an MRI scan and a MEG combined with EEG (M/EEG) scan. For the test-retest session, a subset of patients returns for a second M/EEG approximately two weeks after the first. Patients proceed to stage 2, with repeat assessment at 12 months (including M/EEG and MRI, clinical and neuropsychology) and 24-months (clinical and neuropsychological reassessments).

Participant recruitment and selection

Participants are aged between 50 and 85 years with similar numbers of men and women, with symptomatic mild cognitive impairment or early dementia due to Alzheimer's Disease (n=100) or normal cognition (n=30, Figure 1). Potential participants are identified by a staged screening process. First, approved clinical staff undertake electronic screening of records held by regional memory clinics or NIHR Join Dementia Research, or research team members review electronic records of people who previously consented to data retention on a contact list regarding dementia research opportunities. A participant information sheet detailing the study procedures is provided to candidate participants and a study partner who regularly sees the participant and is willing to complete assessments. Candidate participants answer a series of questions derived from the inclusion and exclusion criteria (Table 1) during a telephone-based screening call.

The still potentially eligible participants are invited to on-site screening (see below). For those with Alzheimer's disease or mild cognitive impairment, clinical letters are sought from their memory assessment service, in addition to within-study assessments. This final

For peer review only - http://bmjopen.bmj.com/site/about/guidelines.xhtml

screening appointment allows further assessment of eligibility, against exclusion and inclusion criteria (Table 1) and Alzheimer biomarker status. Any uncertainty regarding eligibility is discussed with the principal investigator for the relevant site. Eligible people proceed to Stage 1.

For peer review only

Group inclusion criteria	
**Patients**	**Controls**
Diagnosis of MCI or AD#	No neurological diagnosis
CDR = 0.5 – 1	CDR = 0
Positive AD biomarker status (CSF or PET)	Known AD biomarker status (CSF or PET)
MMSE > 18	MMSE > 24
50-85 years	50-85 years

General exclusion criteria (patients and controls)
Significant neurological disease, other than Alzheimer's disease, that may affect cognition or ability to complete the study
Presence of any significant psychiatric disorder that could affect participation [86]
Any clinically important abnormality that could compromise study participation
A clinically significant illness, medical or surgical procedure, or trauma within 30 days prior to screening or baseline
Known or suspected systemic infection
Medications affecting cognition, unless on a stable dose for >30 days prior to baseline
Rosen Modified Hachinski Ischaemic score ≥ 4
History of seizure, except febrile seizures or single provoked seizure
Head trauma resulting in protracted loss of consciousness, or serious infectious disease affecting the brain, within five years of screening and baseline
Participation in a clinical trial of an investigational medicinal product
Impairment of vision or hearing that could affect study participation
Formal education ≤ 7 years
Lack of mental capacity or other ability to consent
Inability to read and write fluently in English
Inability to walk 10 metres independently*
Contraindications to blood sampling
Contraindications to lumbar puncture (e.g. spinal deformations) and amyloid PET scan
Contraindication to MRI (including, but not limited to, claustrophobia; pregnancy; MR-incompatible pacemakers and other MR-incompatible, implanted medical devices)
Metallic implants in the body that affect MEG recordings, as judged by the Investigator

***Table 1 Group inclusion criteria and general exclusion criteria**. MCI=Mild Cognitive Impairment, AD=Alzheimer's Disease, MMSE=Mini Mental State Examination, CDR=Clinical Dementia Rating. # according to* [85] or [54]. ** Ten metre walking is required for practicality and safety during the scanning sessions to avoid the use of walking aids which may have metallic constituents.*

Sample Size and Power

Previously published studies have detected the presence and progression of Alzheimer's disease or mild cognitive impairment with under 100 participants using MRI.[39] We aim to identify MEG measures that are at least as sensitive as MRI. For longitudinal analysis, MEG[46–48] and EEG[40–45] have been used over 11-36 months with 20-130 participants. Mandal and colleagues[49] summarise the accuracies of MEG measures to distinguish Alzheimer's disease from controls: 30/34 MEG measures had areas under the curve >0.64 approximating under Gaussian assumptions to Cohen's d>0.5.[50]

Power analyses were conducted in G*power.[51] For the cross-sectional NTAD data with 130 participants, for power of 80% and α=0.05, a one-tailed test should detect (i) medium, group-wise effect sizes of >0.5 and (ii) correlations with disease severity of >0.25. With 20% attrition in follow up (n=80) and α=0.05, longitudinal analyses of disease progression would have 80% power to detect within-sample effect size >0.28 (Figure 2).

STUDY PROCEDURES

Overview of protocol stages

Screening

Potential participants are identified from electronic records and screened further via telephone call (see 'Participant recruitment and selection' section). Prospective participants who remain eligible are invited to an in-person screening appointment. Any further questions they have are answered before they provide written, informed consent. After written, informed consent, a medically-qualified clinician administers the clinical interview, clinical dementia rating and Haschinski ischemic scale. In the context of this study, medically qualified means having at least MRCP part II qualifications and in ST3+ level specialty training (neurology or psychiatry), or completion of neurology or psychiatry specialist training. The remaining clinical assessments are completed by a member of the research team and include physiology and blood sampling. All research team members have current Good Clinical Practice training. If Alzheimer biomarker status is unknown from recent clinical or research assessments, participants proceed to either cerebrospinal fluid examination or amyloid PET imaging according to participant preference and eligibility. For patients, where a participant's positive amyloid status has been confirmed previously, a positive result enables participation, a negative result excludes participation. For controls, the Alzheimer biomarker status must be known to proceed but, given their CDR=0, a positive test does not prevent participation.

Stage 1: Baseline

One hundred people with mild cognitive impairment or Alzheimer's disease and 30 neurologically normal people proceed to baseline assessment. Participants undergo structured neuropsychological assessment, an M/EEG scan and MRI imaging of brain structure and function over two sessions (or three by preference).

Stage 1: Two-week M/EEG retest

We invite 30 people from the patient group (i.e. mild cognitive impairment or Alzheimer's disease) to repeat the M/EEG scan between two and four weeks after the first scan. Invitations are prioritised to people who can most readily attend the additional session (e.g. considering distance) until the target sample size is reached.

Stage 2: Annual follow-up 1

Participants in the patient group repeat the clinical and neuropsychological assessments, M/EEG scan, MRI scan and blood collection at 12 months after baseline.

Stage 2: Annual follow-up 2

Clinical and neuropsychological assessments are repeated at 24 months for participants in the patient group.

The study timeline is illustrated in Figure 3.

Blood Samples

Participants are asked to consume only water for two hours prior to blood collection. At baseline and follow-up, blood is drawn in the following order: 2.7ml in Sodium Citrate Tubes (plasma), 5ml in Serum Separator Tubes (serum), 10ml in EDTA tubes (DNA), 10ml in EDTA tubes (plasma and buffy coat) and 2x2.5ml in PAXgene tubes (RNA). Once filled, the EDTA and PAXgene tubes are inverted 10 times. From April 2021, 4.9ml of blood is collected in S-monovette tubes for SARS-CoV-2 serology. Samples are processed and frozen according to the guidelines in Table 2.

**Plasma**	The sample in the sodium citrate tube is centrifuged at 2000g for 10 minutes at room temperature. The plasma is aliquoted into 1.4 ml matrix tubes and stored at -80°C.
**Serum**	At least 30 minutes after blood collection, the sample in the serum separator tube is centrifuged at 2000g for 10 min at room temperature. The serum is aliquoted into 1.4ml matrix tubes and stored at -80°C.<br><br>Similarly, the sample in the S-monovette tube is centrifuged at 2000g for 10 minutes at room temperature at least 30 minutes after collection. The sample is aliquoted into 2ml Sarstedt tubes.
**DNA**	The first EDTA tube is frozen as soon as possible and stored at -80°C for later DNA extraction and analysis.
**Plasma and buffy coat**	The second EDTA tube is centrifuged immediately at 2500g for 15 minutes at room temperature. The plasma and buffy coat are aliquoted into separate 1.4 ml matrix tubes and stored at -80°C.
**RNA**	Paxgene tubes are stored at room temperature for 2 hours before being frozen upright at -20°C for 24 hours and then stored at -80°C.

Table 2 Blood sample processing guidelines

Biomarkers for Alzheimer disease pathology

Clinical criteria are insufficient to reliably diagnose the presence of Alzheimer's disease pathology.[52,53] We seek additional biomarker evidence, using either cerebrospinal fluid or PET imaging according to participant preference and eligibility.[54]

Cerebrospinal fluid

Cerebrospinal fluid is obtained by lumbar puncture and collected in polypropylene tubes. Within one hour of collection, cerebrospinal fluid is centrifuged, separated and the

supernatant frozen to −80°C for later batched analysis using a chemiluminescent enzyme immunoassay for total tau, phosphorylated tau and amyloid beta 1-42 levels. Positive amyloid status is indicated by a total tau to amyloid beta 1-42 ratio >1, and amyloid beta 1-42 concentration <450pg/ml. pTau181 concentration is recorded from April 2021.

Amyloid PET

Participants receive a 300 MBq bolus injection of florbetaben and are scanned 80-100 minutes post-injection on a GE Signa PET/MR scanner at the Wolfson Brain Imaging Centre, Cambridge or a GE D710 PET/CT scanner at the Churchill Hospital, Oxford. The centiloid method is used to classify the florbetaben scans as amyloid positive with centiloid >1.19.[55,56]

Clinical Assessments

Clinical assessments are completed at a clinical research centre in one or two sessions.

Clinical Interview

A study clinician interviews the participant and study partner. Clinicians follow a structured interview that covers socio-demographic factors, including age and years of education; lifestyle factors; family history of dementia; medical history, including information on significant medical conditions with date of onset; and concomitant medication usage, with details about dosage and duration.

Addenbrooke's Cognitive Examination

The revised Addenbrooke's Cognitive Examination evaluates orientation, memory, verbal fluency, language and visuo-spatial domains.[57] It is administered and scored according to the Administration and Scoring Guide (2006). Alternate versions at each visit reduce practice effects.

Mini-Mental State Examination

The Mini-Mental State Examination is acquired.[58]

Clinical Dementia Rating

The Clinical Dementia Rating quantifies dementia severity through a structured interview.[59–61] Interviewers rate participant impairments in 6 categories: memory, orientation, judgment and problem solving, community affairs, home and hobbies and personal care. The global CDR is derived from these ratings.[60]

Haschinski Ischaemic Score

The Rosen modification of Haschinski's Ischaemic Score seeks to differentiate between primary progressive dementias, including Alzheimer's disease and multi-infarct dementia.[62] A study clinician uses information from medical history, physical and neurological examination and medical records to determine the score. Scores below 4 indicate a low likelihood of vascular disease as the cause of dementia.

Self-reported questionnaires

Participants complete the 30-item Geriatric Depression Scale,[63] 40-item Spielberger State-Trait Anxiety Inventory,[64] and 11-item Pittsburgh Sleep Quality Index.[65] Study partners

For peer review only

complete the 30-item Amsterdam Instrumental Activity of Daily Living Questionnaire (short version)[66] and Mild Behavioral Impairment Checklist.[67]

Physiological measures

Height, weight, hip-waist ratio, blood pressure, pulse rate and temperature are measured for participants using a stadiometer, electronic weight scales and stretch-resistant tape.

Neuropsychological Assessment

The neuropsychological test battery closely resembles the Deep and Frequent Phenotyping study[68] and IMI-European Prevention of Alzheimer's Disease,[69,70] including the Pre-Alzheimer Cognitive Composite.[71] Assessments take place in a private, testing room in one session with breaks as needed (Table 3).

Order of assessments	Duration (minutes)
1. List learning (RBANS subtest)<br>2. Story memory (RBANS subtest)<br>3. Figure Copy (RBANS subtest)<br>4. Line orientation (RBANS subtest)<br>5. Picture naming (RBANS subtest)<br>6. Semantic fluency (RBANS subtest)<br>7. Digit span forwards (RBANS subtest)<br>8. Digit span backwards<br>9. Coding (RBANS subtest)<br>10. List recall (RBANS subtest)<br>11. List recognition (RBANS subtest)<br>12. Story recall (RBANS subtest)<br>13. Figure recall (RBANS subtest)	≈ 40
Break	≈ 15
14. Free and cued selective reminding test (PACC)<br>15. Logical memory part 1 (PACC)<br>16. National adult reading test<br>17. Digit symbol substitution (PACC)<br>18. Trails B<br>19. Logical memory part 2 (PACC)	≈ 45
Break	≈ 15
20. Reaction time task (CANTAB subtest)<br>21. Paired associates learning (CANTAB subtest)<br>22. Rapid visual processing (CANTAB subtest)<br>23. Spatial working memory (CANTAB subtest)<br>24. Four mountains task	≈ 30

Table 3 Order of neuropsychological assessments. *RBANS=Repeatable Battery for the Assessment of Neuropsychological Status, PACC=Pre-Alzheimer Cognitive Composite, CANTAB = Cambridge Neuropsychological Test Automated Battery*

Repeatable Battery for the Assessment of Neuropsychological Status (RBANS)

RBANS measures cognitive decline in 5 domains: immediate memory, visuospatial, language, attention and delayed memory.[72] Participants receive alternate forms at repeated assessments. The immediate memory index comprises the *list learning subtest*, with immediate recall of 10-items over 4 trials and the *story memory subtest*, with immediate recall of a 12-item story over two trials. The visuospatial index comprises the *figure copy subtest*, which involves copying a geometric figure and the 10-item, *line orientation subtest*. The language index comprises the 10-item, *picture naming subtest* and the *semantic fluency subtest*, where participants name as many exemplars of the given semantic category as possible in 60 seconds. The attention index comprises the *digit span forwards subtest*, involving immediate repetition of increasing digit strings and the *coding subtest*, scored as the total number of correctly coded numbers generated using an item-to-number code within 90 seconds. The delayed memory index comprises the *list recall subtest*, involving free recall of the list learning task; *list recognition*, where participants decide whether a word was included in the list learning task; *story recall*, where participants freely recall the story memory task; and *figure recall*, where participants draw from memory the figure presented in the figure copy task.

Digit Span Backwards

Digit span backwards, from the Wechsler adult intelligent scale,[73] is used in conjunction with RBANS digit span forwards. It follows the RBANS digit span format but participants are asked to immediately repeat in reverse order.

Free and Cued Selective Reminding Test

The Free and Cued Selective Reminding Test assesses episodic memory and distinguishes retrieval from storage deficits.[74] Sixteen pictured items are encoded during the initial learning phase, where participants identify and name items responding to unique semantic cues. After a short delay, participants freely recall all items. Interviewers prompt for each item not recalled using the unique semantic cues from the learning phase. Participants receive alternate forms at repeated assessments.

Logical Memory

Logical Memory, taken from the Wechsler memory scale (third edition), assesses episodic memory.[75] Participants immediately recall short stories they have been read. After a 30-minute delay with intervening tests, participants freely recall the stories and answer yes or no questions testing story recognition.

National Adult Reading Test

The national adult reading test (second edition) estimates premorbid intelligence from printed, irregular words.[76]

Digit Symbol Substitution

The digit symbol substitution from the Wechsler Adult Intelligence Scale assesses processing speed and attention.[73] Participants have 90 seconds to code as many correct symbols as possible corresponding to presented numbers by using the given number-to-symbol code.

Trails Making Test B

The Trails Making Test B assesses executive function, attention and processing speed.[77] Following a practice sample to ensure task comprehension, participants are presented with 25

For peer review only

encircled numbers on a page which they connect by alternating between numbers and letters. The time it takes to complete the sample and any errors made are recorded.

Cambridge Neuropsychological Test Automated Battery

The tablet-based Cambridge Neuropsychological Test Automated Battery assesses processing speed, episodic memory, attention, working memory and executive function.[78] The reaction time task (simple and five choice variant) assesses processing speed. Participants hold down a response button and release this to respond to on-screen targets. The paired-associates learning task (standard variant) assesses episodic memory. Participants learn associations between patterns and their locations. An initial learning stage precedes immediate recall. The rapid visual processing task (3 target variant) assesses attention. Single digits appear on the screen. Participants respond when they see a string matching target sequences. The spatial working memory task (standard variant) assesses working memory and executive function. Participants search inside on-screen boxes to find and collect tokens. Tokens never appear in the same box twice.

Four Mountains Task

The tablet-based four mountains task assesses allocentric spatial processing.[79] During a learning phase, participants learns the topographical layout of 4 mountains presented in a computer-generated landscape. Following a delay, participants are presented with four alternative images and identify the target image matching the topographical mountain layout of the learning-phase image, but with potentially altered colours, textures and points of view.

Neurophysiology (M/EEG)

M/EEG data are collected simultaneously at 1000Hz in a magnetically-shielded room. At Cambridge, data are collected using the Elekta VectorView system from 2017 to December 2019, with 204 planar gradiometers, 102 magnetometers and an EEG 70-channel Easycap. Stage 1 scans and the first 12 follow-up scans used the same scanner. The MEGIN Triux Neo M/EEG scanner is used from March 2020 onwards with the same sensor configuration as the VectorView and a 64-channel Easycap. At Oxford, the MEGIN Triux Neo M/EEG scanner and an EasyCap 60 channel BrainCap for MEG with an augmented 10/20 layout are used for all data collection.

The Polhemus digitisation system records the position of the standard fiducial points, >300 additional head points, five head position indicator coils and EEG electrodes. Head position indicator coils measure head position within the MEG helmet. Electrodes on the right clavicle and left lower rib record electrocardiogram data. Electrodes above and below the left eye and on bilateral canthi record vertical and horizontal electrooculogram data. Reference and ground electrodes are placed on the left side of the nose and left cheek, respectively. During the seated scan, participants rest or perform simple tasks, responding through a button box. Participants wear non-magnetic earphones with sound delivered though plastic tubes and, if necessary, non-magnetic glasses. Prior to M/EEG, a Snellen eye test and pure tone audiometry assess sight and hearing thresholds. Tasks are carried out in the following order.

Simple audio-visual task

Participants fixate on a red, central fixation dot and quickly respond to each auditory or visual stimuli with a button press (Figure 4a). Auditory tones (n=100) of 300Hz, 600Hz or 1200Hz are presented for 300ms after a blank interval of 1000ms. Visual stimuli are concentric black and white circles that appear for 300ms after 3000ms. Filler trials (n=30)

For peer review only

containing only the red fixation dot are included. Visual and auditory trials are randomly intermixed. Ten initial practice trials familiarise participants to the task.

Auditory Mismatch-negativity Task

The roving auditory mismatch negativity task elicits error responses to deviant tones followed by rapid plasticity as predictions are updated upon repetition of the deviant stimulus.[80,81] Participants passively watch a muted nature documentary. Through earpieces, they hear binaural, in-phase sinusoidal tones >60dB above the average auditory threshold with durations of 100ms and 500ms stimulus onset asynchrony. Tone frequencies are the same within but different between blocks. Blocks range from 400 to 800Hz. The number of tones per blocks varies from 3 to 11 according to a truncated exponential distribution (Figure 4b).

Scene-Repetition Task

In this passive memory test, participants view a series of complex scenes (landscapes and cityscapes) and press a button only when scenes contain a moon (n=26, different moon images). Target scenes containing a moon ensure attention but are not of interest; the main interest is differences between initial and repeated presentations of non-target scenes. Each scene is presented for 800ms and preceded by a fixation cross of 200ms on average (100-300ms). Scenes are pseudo-randomly intermixed with the constraint that 10 initial 'burn-in' complex scenes, with 2 moon target scenes, precede 256 complex scenes presented twice with 14-93 (median=42) intervening scenes between the first and repeat presentation (Figure 4c).

Cross-modal Oddball Task

The task assesses hippocampal-dependent, paired-associates learning.[82,83] Trials comprise of a 700ms visual, abstract "object" and a 400ms sound, starting 300ms after trial onset. Inter-stimulus intervals average 300ms. During the initial training period (80 trials), participants learn associations between four standard pairs of visual objects and sounds. The main task has 770 bimodal trials and 40 unimodal trials, randomly intermixed. Bimodal trials consist of: standard, learnt object-sound pairs (n=670); standard objects paired with novel sounds (n=50); and mismatched pairs, where the object and sound are from different standard pairs (n=50). Unimodal trials ensure the task is attended, participants press a button when they see the letter 'a' (Figure 4d). A 10-item assessment follows the task, participants hear a standard sound and report which of four presented objects the sound was paired with most often during the task.

Eyes-open Resting State

Participants are presented with a central fixation cross and instructed to clear their mind, relax, think of nothing specific and focus on the fixation cross for 5 minutes.

Eyes-closed Resting State

Participants are instructed to clear their mind, relax, think of nothing specific and close their eyes but stay awake.

Magnetic Resonance Imaging

MRI sequences use 3T Siemens PRISMA scanners at the Cambridge MRC Cognition and Brain Science Unit and Oxford Centre for Human Brain Activity (Table 4).

For peer review only

**T1-weighted**	The 3D T1-weighted structural image is acquired using the Generalized Autocalibrating Partially Parallel Acquisitions (GRAPPA) technique applied to a high-resolution Magnetization Prepared Rapid Gradient Echo (MPRAGE) sequence with the following parameters: TE=2.91ms, TR = 2300.00ms, TI=900.00ms, flip angle =9 degrees, acquisition matrix =256 x 240, voxel size =1.00mm isotropic, number of slices =176, slice thickness =1mm, acquisition time =5 minutes and 12 seconds
**T2 FLAIR**	The 3D FLAIR structural image is acquired using the following parameters: TE=394.00ms, TR =5000.00ms, TI =1650.00ms, flip angle =120 degrees, acquisition matrix =256 x 256, voxel size =1.00mm isotropic, number of slices =192, slice thickness =1.00mm, acquisition time =5 minutes and 7 seconds
**T2*-weighted**	The T2*-weighted structural image is acquired using the following parameters: TE =20.00ms, TR =640.00ms, Flip angle =20 degrees, acquisition matrix =256 x 256, number of slices =47, slice thickness =3.00mm, Voxel size =0.90x0.90x3.00mm, Acquisition time =4 minutes and 7 seconds
**T2-weighted with fat saturation**	The T2-weighted image with fat saturation is acquired with the following parameters: TE =78.00ms, TR =4000.00ms, flip angle =150 degrees, acquisition matrix =256x232, number of slices =47, slice thickness=3.00mm, voxel size =0.90x0.90x3.00mm, acquisition time =3 minutes and 26 seconds
**Diffusion Weighted Imaging**	The Diffusion-Weighted Images are acquired with a spin-echo echo-planar sequence. B-values of 0, 300, 700 and 2000 s/mm^2 were used with 116 diffusion gradient directions obtained via MRTrix3.[84] The number of signal averages was 12, 8, 32, and 64 for b-values of 0, 300, 700 and 2000, respectively. The acquisition is run in the transverse orientation without any angulation. Other scan parameters include: TR=3800ms; TE=85.00ms, voxel size=2.50mm isotropic, acquisition matrix =96x96, number of slices=64, slice thickness =2.50mm, acquisition time=8 minutes and 45 seconds
**Quantitative Susceptibility Mapping**	The quantitative susceptibility mapping correlates with Aβ-amyloid iron density and is acquired with a 3D Gradient Echo sequence with multiple echoes and flow compensation in the readout and slice directions. (TE 1=5.20ms, TE 2=10.4ms, TE 3 =15.6ms, TE 4 =20.8ms, TE 5=26.0ms; TR =31.0ms, flip angle =15 degrees, acquisition matrix =256x192, number of slices =160 (per TE), slice thickness =1.00mm, voxel size =1.00mm isotropic, acquisition time =5 minutes 10 seconds.
**Resting state eyes open**	For the resting-state scan, the participants are presented with a fixation cross and asked to maintain fixation throughout the task. Echo planar images are acquired with 200 volumes with the following parameters: TE=30.00ms, TR=1500.00ms, flip angle =90 degrees, acquisition matrix =64x64, number of slices =54, slice thickness =3.00mm, voxel size = 3.00mm isotropic, acquisition time =5 minutes 50 seconds
**Hippocampal subfields**	The high-resolution, hippocampal subfield images are acquired with T2-weighted, turbo spin echo sequences and run with the following

For peer review only - http://bmjopen.bmj.com/site/about/guidelines.xhtml

	parameters: TE=50.00ms, TR=8020.00ms, flip angle =122 degrees, acquisition matrix =448x448, number of slices =30, slice thickness =2.00mm, voxel size =0.4x0.4x2.0mm, acquisition time =8 minutes 11 seconds.
**Arterial Spin Labelling**	3D Arterial Spin Labelling images are acquired using a flow-sensitive alternating inversion recovery spin-echo pulsed sequence with Q2-TIPS bolus saturation in the transverse plane with the following parameters: TR=4000.00ms; TE =13.20ms; flip angle =130 degrees, labelling duration =800ms, post-labelling delay =2000ms, acquisition matrix =64x60; slices per slab =32, slice thickness =4.50mm, voxel size =1.85x1.85x4.50mm, acquisition time =6 minutes 46 seconds.

Table 4 Order and parameters of MRI sequences. *FLAIR=Fluid Attenuated Inversion Recovery, TE=Echo time, TR=Repetition time, TI=Inversion time*

CURRENT STATUS

The study is active at both sites.

ETHICS AND DISSEMINATION

The study received a favourable opinion from the East of England – Cambridge Central Research Ethics Committee (REC reference 18/EE/0042). Imaging data and clinical severity scores are hosted by Dementias Platform UK Imaging Platform (https://portal.dementiasplatform.uk), using XNAT (https://www.xnat.org). Data will be made available with a managed access process through Dementias Platform UK, with constraints to preserve confidentiality and privacy.

For peer review only

Figure Legends

Figure 1 Participant flow chart. A) Recruitment strategy: Electronic screening of records from regional memory clinics and Join Dementia Research generates approximately 300 potential patient and 1000 potential patient and control participants who are invited to telephone screening. People who remain eligible are screened further on-site to identify 100 patient and 30 control participants. B) Study stages: Stage 1 consists of the cross-sectional baseline assessments and, for 30 patients, a repeat of the M/EEG assessment two weeks after the first. Only patients continue to the longitudinal stage 2 of the study, with repeat assessments 12 and 24 months after baseline. Attrition of 20% is expected at stage 2. M/EEG=Magnetoencephalography combined with electroencephalography imaging, MRI=Magnetic resonance imaging

Figure 2 Effect sizes required to detect longitudinal correlations with disease severity at different attrition rates (resulting in reduced longitudinal sample size) with 80% power and α=0.05. We expect <20% attrition.

Figure 3 Timeline summary of study procedures

Figure 4 a) Audio-visual task: Participants visually fixate on a red dot and press a button whenever they hear or see something. (b) Mismatch-negativity task: Participants passively watch a nature documentary while listening to tones through the earpieces. Red dashes represent deviant tones and black dashes represent standard tones (from the sixth repetition). (c) Scene repetition task: Participants view scenes and press the button when they see a scene containing a moon. Each non-target scene repeats once during the task. (d) Cross-modal oddball task: Participants view object-tone pairs consisting of 4 learnt (standard) pairs (the standard sounds are black in the figure); associative-deviant, object-tone pairs (the associative deviant sound is coloured red in the figure); and standard objects presented with novel tones (the novel deviant tones are blue in the figure). Participants press a button when they see the letter ‘a’.

For peer review only

REFERENCES

1 LaFerla FM, Green KN. Animal models of Alzheimer disease. *Cold Spring Harb Perspect Med* 2012;**2**:1–13. doi:10.1101/cshperspect.a006320

2 Drummond E, Wisniewski T. Alzheimer's disease: experimental models and reality. Acta Neuropathol. 2017;**133**:155–75. doi:10.1007/s00401-016-1662-x

3 Cummings JL, Morstorf T, Zhong K. Alzheimer's disease drug-development pipeline: Few candidates, frequent failures. *Alzheimer's Res Ther* 2014;**6**:1–7. doi:10.1186/alzrt269

4 Yiannopoulou KG, Anastasiou AI, Zachariou V, *et al.* Reasons for failed trials of disease-modifying treatments for alzheimer disease and their contribution in recent research. Biomedicines. 2019;**7**. doi:10.3390/biomedicines7040097

5 Anderson RM, Hadjichrysanthou C, Evans S, *et al.* Why do so many clinical trials of therapies for Alzheimer's disease fail? Lancet. 2017;**390**:2327–9. doi:10.1016/S0140-6736(17)32399-1

6 Mehta D, Jackson R, Paul G, *et al.* Why do trials for Alzheimer's disease drugs keep failing? *Expert Opin Investig Drugs* 2017;**26**:735–9. doi:10.1080/13543784.2017.1323868.Why

7 Frisoni GB, Fox NC, Jack CR, *et al.* The clinical use of structural MRI in Alzheimer disease. *Nat Rev Neurol* 2010;**6**:67–77. doi:10.1038/nrneurol.2009.215

8 Holland D, McEvoy LK, Dale AM. Unbiased comparison of sample size estimates from longitudinal structural measures in ADNI. *Hum Brain Mapp* 2012;**33**:2586–602. doi:10.1002/hbm.21386

9 Leung KK, Barnes J, Ridgway GR, *et al.* Automated cross-sectional and longitudinal hippocampal volume measurement in mild cognitive impairment and Alzheimer's disease. *Neuroimage* 2010;**51**:1345–59. doi:10.1016/j.neuroimage.2010.03.018

10 Cope TE, Rittman T, Borchert RJ, *et al.* Tau burden and the functional connectome in Alzheimer's disease and progressive supranuclear palsy. *Brain* 2018;**141**:550–67. doi:10.1093/brain/awx347

11 Passamonti L, Rodríguez PV, Hong YT, *et al.* PK11195 binding in Alzheimer disease and progressive supranuclear palsy. *Neurology* 2018;**90**:e1989–96. doi:10.1212/WNL.0000000000005610

12 Passamonti L, Rodríguez PV, Hong YT, *et al.* 18F-AV-1451 positron emission tomography in Alzheimer's disease and progressive supranuclear palsy. *Brain* 2017;**140**:781–91. doi:10.1093/brain/aww340

13 Leuzy A, Chiotis K, Lemoine L, *et al.* Tau PET imaging in neurodegenerative tauopathies—still a challenge. Mol. Psychiatry. 2019;**24**:1112–34. doi:10.1038/s41380-018-0342-8

14 Ossenkoppele R, Schonhaut DR, Schö M, *et al.* Tau PET patterns mirror clinical and neuroanatomical variability in Alzheimer's disease. *Brain* 2016;**139**:1551–67. doi:10.1093/brain/aww041

15 Cai L, Innis R, Pike V. Radioligand Development for PET Imaging of beta-Amyloid (AD)-Current Status. *Curr Med Chem* 2006;**14**:19–52. doi:10.2174/092986707779313471

For peer review only

16 Bevan-Jones WR, Surendranathan A, Passamonti L, *et al.* Neuroimaging of inflammation in memory and related other disorders (NIMROD) study protocol: A deep phenotyping cohort study of the role of brain inflammation in dementia, depression and other neurological illnesses. *BMJ Open* 2017;**7**. doi:10.1136/bmjopen-2016-013187

17 Koffie RM, Hyman BT, Spires-Jones TL. Alzheimer's disease: Synapses gone cold. Mol. Neurodegener. 2011;**6**:1–9. doi:10.1186/1750-1326-6-63

18 Palop JJ, Mucke L. Amyloid-B-induced neuronal dysfunction in Alzheimer's disease: From synapses toward neural networks. Nat. Neurosci. 2010;**13**:812–8. doi:10.1038/nn.2583

19 Kashyap G, Bapat D, Das D, *et al.* Synapse loss and progress of Alzheimer's disease - A network model. *Sci Rep* 2019;**9**:1–9. doi:10.1038/s41598-019-43076-y

20 De Wilde MC, Overk CR, Sijben JW, *et al.* Meta-analysis of synaptic pathology in Alzheimer's disease reveals selective molecular vesicular machinery vulnerability. *Alzheimer's Dement* 2016;**12**:633–44. doi:10.1016/j.jalz.2015.12.005

21 Henstridge CM, Sideris DI, Carroll E, *et al.* Synapse loss in the prefrontal cortex is associated with cognitive decline in amyotrophic lateral sclerosis. *Acta Neuropathol* 2018;**135**:213–26. doi:10.1007/s00401-017-1797-4

22 Menkes-Caspi N, Yamin HG, Kellner V, *et al.* Pathological tau disrupts ongoing network activity. *Neuron* 2015;**85**:959–66. doi:10.1016/j.neuron.2015.01.025

23 Pinotsis DA, Schwarzkopf DS, Litvak V, *et al.* Dynamic causal modelling of lateral interactions in the visual cortex. *Neuroimage* 2013;**66**:563–76. doi:10.1016/j.neuroimage.2012.10.078

24 Adams NE, Hughes LE, Phillips HN, *et al.* GABA-ergic dynamics in human frontotemporal networks confirmed by pharmaco-magnetoencephalography. *J Neurosci* 2020;:1689–19. doi:10.1523/jneurosci.1689-19.2019

25 Sami S, Williams N, Hughes LE, *et al.* Neurophysiological signatures of Alzheimer's disease and frontotemporal lobar degeneration: pathology versus phenotype. *Brain* 2018;**141**:2500–10. doi:10.1093/brain/awy180

26 Sitnikova TA, Hughes JW, Ahlfors SP, *et al.* Short timescale abnormalities in the states of spontaneous synchrony in the functional neural networks in Alzheimer's disease. *NeuroImage Clin* 2018;**20**:128–52. doi:10.1016/j.nicl.2018.05.028

27 Puttaert D, Coquelet N, Wens V, *et al.* Alterations in resting-state network dynamics along the Alzheimer's disease continuum. *Sci Rep* 2020;**10**:1–13. doi:10.1038/s41598-020-76201-3

28 Hughes LE, Henson RN, Pereda E, *et al.* Biomagnetic biomarkers for dementia: A pilot multicentre study with a recommended methodological framework for magnetoencephalography. *Alzheimer's Dement Diagnosis, Assess Dis Monit* 2019;**11**:450–62. doi:10.1016/j.dadm.2019.04.009

29 Hughes LE, Nestor PJ, Hodges JR, *et al.* Magnetoencephalography of frontotemporal dementia: Spatiotemporally localized changes during semantic decisions. *Brain* 2011;**134**:2513–22. doi:10.1093/brain/awr196

30 Hughes LE, Rittman T, Robbins TW, *et al.* Reorganization of cortical oscillatory dynamics underlying disinhibition in frontotemporal dementia. *Brain* 2018;**141**:2486–

For peer review only - http://bmjopen.bmj.com/site/about/guidelines.xhtml

99. doi:10.1093/brain/awy176

31 Hughes LE, Rittman T, Regenthal R, *et al.* Improving response inhibition systems in frontotemporal dementia with citalopram. *Brain* 2015;**138**:1961–75. doi:10.1093/brain/awv133

32 Hughes LE, Rowe JB. The impact of neurodegeneration on network connectivity: A study of change detection in frontotemporal dementia. *J Cogn Neurosci* 2013;**25**:802–13. doi:10.1162/jocn_a_00356

33 van Dellen E, de Waal H, van der Flier WM, *et al.* Loss of EEG Network Efficiency Is Related to Cognitive Impairment in Dementia With Lewy Bodies. *Mov Disord* 2015;**30**:1785–93. doi:10.1002/mds.26309

34 Woolrich MW, Baker A, Luckhoo H, *et al.* Dynamic state allocation for MEG source reconstruction. *Neuroimage* 2013;**77**:77. doi:10.1016/J.NEUROIMAGE.2013.03.036

35 Baker AP, Brookes MJ, Rezek IA, *et al.* Fast transient networks in spontaneous human brain activity. *Elife* 2014;**2014**. doi:10.7554/ELIFE.01867

36 Vidaurre D, Quinn AJ, Baker AP, *et al.* Spectrally resolved fast transient brain states in electrophysiological data. *Neuroimage* 2016;**126**:81–95. doi:10.1016/j.neuroimage.2015.11.047

37 Adams NE, Hughes LE, Rouse MA, *et al.* GABAergic cortical network physiology in frontotemporal lobar degeneration. *Brain* 2021;**144**:2135–45. doi:10.1093/BRAIN/AWAB097

38 Shaw AD, Muthukumaraswamy SD, Saxena N, *et al.* Generative modelling of the thalamo-cortical circuit mechanisms underlying the neurophysiological effects of ketamine. *Neuroimage* 2020;**221**:117189. doi:10.1016/j.neuroimage.2020.117189

39 Holland D, Brewer JB, Hagler DJ, *et al.* Subregional neuroanatomical change as a biomarker for Alzheimer's disease. *Proc Natl Acad Sci U S A* 2009;**106**:20954. doi:10.1073/PNAS.0906053106

40 Bennys K, Rondouin G, Benattar E, *et al.* Can event-related potential predict the progression of mild cognitive impairment? *J Clin Neurophysiol* 2011;**28**:625–32. doi:10.1097/WNP.0B013E31823CC2D3

41 Lai CL, Lin RT, Liou LM, *et al.* The role of event-related potentials in cognitive decline in Alzheimer's disease. *Clin Neurophysiol* 2010;**121**:194–9. doi:10.1016/J.CLINPH.2009.11.001

42 Fruehwirt W, Dorffner G, Roberts S, *et al.* Associations of event-related brain potentials and Alzheimer's disease severity: A longitudinal study. *Prog Neuropsychopharmacol Biol Psychiatry* 2019;**92**:31–8. doi:10.1016/J.PNPBP.2018.12.013

43 Papaliagkas VT, Kimiskidis VK, Tsolaki MN, *et al.* Cognitive event-related potentials: Longitudinal changes in mild cognitive impairment. *Clin Neurophysiol* 2011;**122**:1322–6. doi:10.1016/J.CLINPH.2010.12.036

44 Chapman RM, McCrary JW, Gardner MN, *et al.* Brain ERP components predict which individuals progress to Alzheimer's disease and which do not. *Neurobiol Aging* 2011;**32**:1742–55. doi:10.1016/J.NEUROBIOLAGING.2009.11.010

45 Papaliagkas V, Kimiskidis V, Tsolaki M, *et al.* Usefulness of event-related potentials in the assessment of mild cognitive impairment. *BMC Neurosci* 2008;**9**:1–10.

For peer review only

doi:10.1186/1471-2202-9-107/TABLES/4

46 Pusil S, López ME, Cuesta P, *et al.* Hypersynchronization in mild cognitive impairment: the 'X' model. *Brain* 2019;**142**:3936–50. doi:10.1093/BRAIN/AWZ320

47 Fernández A, Turrero A, Zuluaga P, *et al.* Magnetoencephalographic Parietal δ Dipole Density in Mild Cognitive Impairment: Preliminary Results of a Method to Estimate the Risk of Developing Alzheimer Disease. *Arch Neurol* 2006;**63**:427–30. doi:10.1001/ARCHNEUR.63.3.427

48 Xu M, Sanz DL, Garces P, *et al.* A Graph Gaussian Embedding Method for Predicting Alzheimer's Disease Progression with MEG Brain Networks. *IEEE Trans Biomed Eng* 2021;**68**:1579–88. doi:10.1109/TBME.2021.3049199

49 Mandal PK, Banerjee A, Tripathi M, *et al.* A Comprehensive Review of Magnetoencephalography (MEG) Studies for Brain Functionality in Healthy Aging and Alzheimer's Disease (AD). *Front Comput Neurosci* 2018;**12**:60. doi:10.3389/FNCOM.2018.00060

50 Salgado JF. Transforming the area under the normal curve (AUC) into cohen's d, pearson's rpb, odds-ratio, and natural log odds-ratio: Two conversion tables. *Eur J Psychol Appl to Leg Context* 2018;**10**:35–47. doi:10.5093/EJPALC2018A5

51 Erdfelder E, FAul F, Buchner A, *et al.* Statistical power analyses using G*Power 3.1: Tests for correlation and regression analyses. *Behav Res Methods* 2009;**41**:1149–60. doi:10.3758/BRM.41.4.1149

52 Ossenkoppele R, Jansen WJ, Rabinovici GD, *et al.* Prevalence of Amyloid PET Positivity in Dementia Syndromes: A Meta-analysis HHS Public Access. *JAMA* 2015;**313**:1939–49. doi:10.1001/jama.2015.4669

53 Monsell SE, Kukull WA, Roher AE, *et al.* Characterizing apolipoprotein E ε4 carriers and noncarriers with the clinical diagnosis of mild to moderate Alzheimer dementia and minimal β-amyloid peptide plaques. *JAMA Neurol* 2015;**72**:1124–31. doi:10.1001/jamaneurol.2015.1721

54 McKhann GM, Knopman DS, Chertkow H, *et al.* The diagnosis of dementia due to Alzheimer's disease: Recommendations from the National Institute on Aging-Alzheimer's Association workgroups on diagnostic guidelines for Alzheimer's disease. *Alzheimer's Dement* 2011;**7**:263–9. doi:10.1016/j.jalz.2011.03.005

55 Sabri O, Sabbagh MN, Seibyl J, *et al.* Florbetaben PET imaging to detect amyloid beta plaques in Alzheimer's disease: Phase 3 study. *Alzheimer's Dement* 2015;**11**:964–74. doi:10.1016/j.jalz.2015.02.004

56 Rowe CC, Doré V, Jones G, *et al.* 18F-Florbetaben PET beta-amyloid binding expressed in Centiloids. *Eur J Nucl Med Mol Imaging* 2017;**44**:2053–9. doi:10.1007/s00259-017-3749-6

57 Mioshi E, Dawson K, Mitchell J, *et al.* The Addenbrooke's Cognitive Examination revised (ACE-R): A brief cognitive test battery for dementia screening. *Int J Geriatr Psychiatry* 2006;**21**:1078–85. doi:10.1002/gps.1610

58 Folstein MF, Folstein SE, McHugh PR. 'Mini-mental state'. A practical method for grading the cognitive state of patients for the clinician. *J Psychiatr Res* 1975;**12**:189–98. doi:10.1016/0022-3956(75)90026-6

59 Hughes CP, Berg L, Danziger WL, *et al.* A new clinical scale for the staging of

For peer review only

dementia. *Br J Psychiatry* 1982;**140**:566–72. doi:10.1192/bjp.140.6.566

60 Morris JC. The clinical dementia rating (cdr): Current version and scoring rules. *Neurology* 1993;**43**:2412–4. doi:10.1212/wnl.43.11.2412-a

61 Washington University Alzheimer's Disease Research Center. The CDR® Dementia Staging Instrument. SCALE. 1999;:1. doi:20 May 1999

62 Rosen WG, Terry RD, Fuld PA, *et al.* Pathological verification of ischemic score in differentiation of dementias. *Ann Neurol* 1980;**7**:486–8. doi:10.1002/ana.410070516

63 Ishihara T, Terada S. [Geriatric Depression Scale (GDS)]. *Nihon Rinsho* 2011;**69 Suppl 8**:455–8. doi:10.1300/j018v05n01_09

64 Spielberger CD, Gorsuch RL, Lushene RE. Manual for the State-Trait Anxiety Inventory. 1970.

65 Buysse DJ, Reynolds CF, Monk TH, *et al.* The Pittsburgh sleep quality index: A new instrument for psychiatric practice and research. *Psychiatry Res* 1989;**28**:193–213. doi:10.1016/0165-1781(89)90047-4

66 Jutten RJ, Peeters CFW, Leijdesdorff SMJ, *et al.* Detecting functional decline from normal aging to dementia: Development and validation of a short version of the Amsterdam IADL Questionnaire. *Alzheimer's Dement Diagnosis, Assess Dis Monit* 2017;**8**:26–35. doi:10.1016/j.dadm.2017.03.002

67 Ismail Z, Agüera-Ortiz L, Brodaty H, *et al.* The Mild Behavioral Impairment Checklist (MBI-C): A Rating Scale for Neuropsychiatric Symptoms in Pre-Dementia Populations. *J Alzheimer's Dis* 2017;**56**:929–38. doi:10.3233/JAD-160979

68 Koychev I, Lawson J, Chessell T, *et al.* Deep and Frequent Phenotyping study protocol: an observational study in prodromal Alzheimer's disease. *BMJ Open* 2019;**9**:24498. doi:10.1136/bmjopen-2018-024498

69 Solomon A, Kivipelto M, Molinuevo JL, *et al.* European Prevention of Alzheimer's Dementia Longitudinal Cohort Study (EPAD LCS): Study protocol. *BMJ Open* 2018;**8**. doi:10.1136/bmjopen-2017-021017

70 Ritchie K, Ropacki M, Albala B, *et al.* Recommended cognitive outcomes in preclinical Alzheimer's disease: Consensus statement from the European Prevention of Alzheimer's Dementia project. Alzheimer's Dement. 2017;**13**:186–95. doi:10.1016/j.jalz.2016.07.154

71 Donohue MC, Sperling RA, Salmon DP, *et al.* The preclinical Alzheimer cognitive composite: Measuring amyloid-related decline. *JAMA Neurol* 2014;**71**:961–70. doi:10.1001/jamaneurol.2014.803

72 Randolph C, Tierney MC, Mohr E, *et al.* The Repeatable Battery for the Assessment of Neuropsychological Status (RBANS): Preliminary clinical validity. *J Clin Exp Neuropsychol* 1998;**20**:310–9. doi:10.1076/jcen.20.3.310.823

73 Wechsler D. *Wechsler Adult Intelligence Scale - Revised*. San Antonio, TX: Psychological Corporation 1981.

74 Grober E, Veroff AE, Lipton RB. Temporal unfolding of declining episodic memory on the Free and Cued Selective Reminding Test in the predementia phase of Alzheimer's disease: Implications for clinical trials. *Alzheimer's Dement Diagnosis, Assess Dis Monit* 2018;**10**:161–71. doi:10.1016/j.dadm.2017.12.004

For peer review only

75 Wechsler D. *Wechsler Memory Scale - Third UK Edition.* London: Harcourt Assessment 1999.

76 Nelson HE, Willison JR. *National Adult Reading Test (NART): Test Manual (2nd edn).* 1991.

77 Bowie CR, Harvey PD. Administration and interpretation of the Trail Making Test. *Nat Protoc* 2006;**1**:2277–81. doi:10.1038/nprot.2006.390

78 Cambridge Cognition. CANTAB® [Cognitive assessment software]. 2019.

79 Hartley T, Bird CM, Chan D, *et al.* The hippocampus is required for short-term topographical memory in humans. *Hippocampus* 2007;**17**:34–8. doi:10.1002/hipo.20240

80 Kiebel SJ, Garrido MI, Friston KJ. Dynamic causal modelling of evoked responses: The role of intrinsic connections. *Neuroimage* 2007;**36**:332–45. doi:10.1016/j.neuroimage.2007.02.046

81 Friston K. A theory of cortical responses. *Philos Trans R Soc B Biol Sci* 2005;**360**:815–36. doi:10.1098/rstb.2005.1622

82 De Rover M, Pironti VA, McCabe JA, *et al.* Hippocampal dysfunction in patients with mild cognitive impairment: A functional neuroimaging study of a visuospatial paired associates learning task. *Neuropsychologia* 2011;**49**:2060–70. doi:10.1016/j.neuropsychologia.2011.03.037

83 Blackwell AD, Sahakian BJ, Vesey R, *et al.* Detecting dementia: Novel neuropsychological markers of preclinical Alzheimer's disease. *Dement Geriatr Cogn Disord* 2004;**17**:42–8. doi:10.1159/000074081

84 Tournier JD, Smith R, Raffelt D, *et al.* MRtrix3: A fast, flexible and open software framework for medical image processing and visualisation. Neuroimage. 2019;**202**. doi:10.1016/j.neuroimage.2019.116137

85 Albert MS, DeKosky ST, Dickson D, *et al.* The diagnosis of mild cognitive impairment due to Alzheimer's disease: Recommendations from the National Institute on Aging-Alzheimer's Association workgroups on diagnostic guidelines for Alzheimer's disease. *Alzheimers Dement* 2011;**7**:270. doi:10.1016/J.JALZ.2011.03.008

86 American Psychiatric Association. *Diagnostic and Statistical Manual of Mental Disorders*. Fifth. American Psychiatric Association 2013. doi:10.1176/appi.books.9780890425596

For peer review only

FOOTNOTES

Author statement: JHL and JBR drafted the manuscript, with review and contribution from all authors. JBR is the chief investigator of the study. RNH, ACN, MW, KDS and VR are principal investigators, contributing to conception and design. EK led protocol development and governance. JBR, MW, ACN, RNH, JI, SL, MP and KDS conceived and designed the study.

Patient and public involvement

The use and tolerability of M/EEG in combination with the PET or CSF, MRI and neuropsychology was discussed as part of Alzheimer Society sponsored patient groups with patients and carers affected by AD/MCI for the closely related Dementias Platform UK study known as 'Deep and frequent phenotyping', with which NTAD is aligned in design and methods. In addition, the use of MEG for dementia research featured in the public engagement event 'MEG and me' as part of the Cambridge Science Festival and has been discussed at open meetings at the Cambridge Science Festival and Alzheimer Research UK Eastern Region network public meetings. The study is also co-funded by Alzheimer Research UK, a charity with a joint patient advocacy and research funding remit.

Funding: This work is part of the Dementias Platform UK which is funded by Medical Research Council (MR/L023784/1 & MR/L023784/2), Janssen, AstraZeneca, Araclon, IXICO, Somalogic, GlaxoSmithKline, Invicro, Cambridge Cognition and Cognetivity. The study has additional support from Alzheimer's Research UK (ARUK-PG2017B-19), the Wellcome Trust (103838), Medical Research Council (SUAG/051 G101400; SUAG/046 G101400), NIHR Cambridge Biomedical Research Centre (BRC-1215-20014) and NIHR Oxford Biomedical Research Centre (BRC-1215-20008). The views expressed are those of the authors and not necessarily those of the NIHR or the Department of Health and Social Care. For the purpose of Open Access, the author has applied a CC BY public copyright licence to any Author Accepted Manuscript version arising from this submission.

Conflicts of interests: John Isaac is employed by Johnson & Johnson and may currently hold Johnson & Johnson stocks. Michael Perkinton is employed by AstraZeneca and may currently hold AstraZeneca stocks or stock options. Yun-Ju Cheng and Stephen Lowe are employed by Eli Lilly and may currently hold Eli Lilly stock. Mark Woolrich receives royalties from FSL and is senior editor for Neuroimage. James Rowe is Chief Scientific Adviser for Alzheimer Research UK and is a trustee of Brain, PSP association and Darwin college.

For peer review only

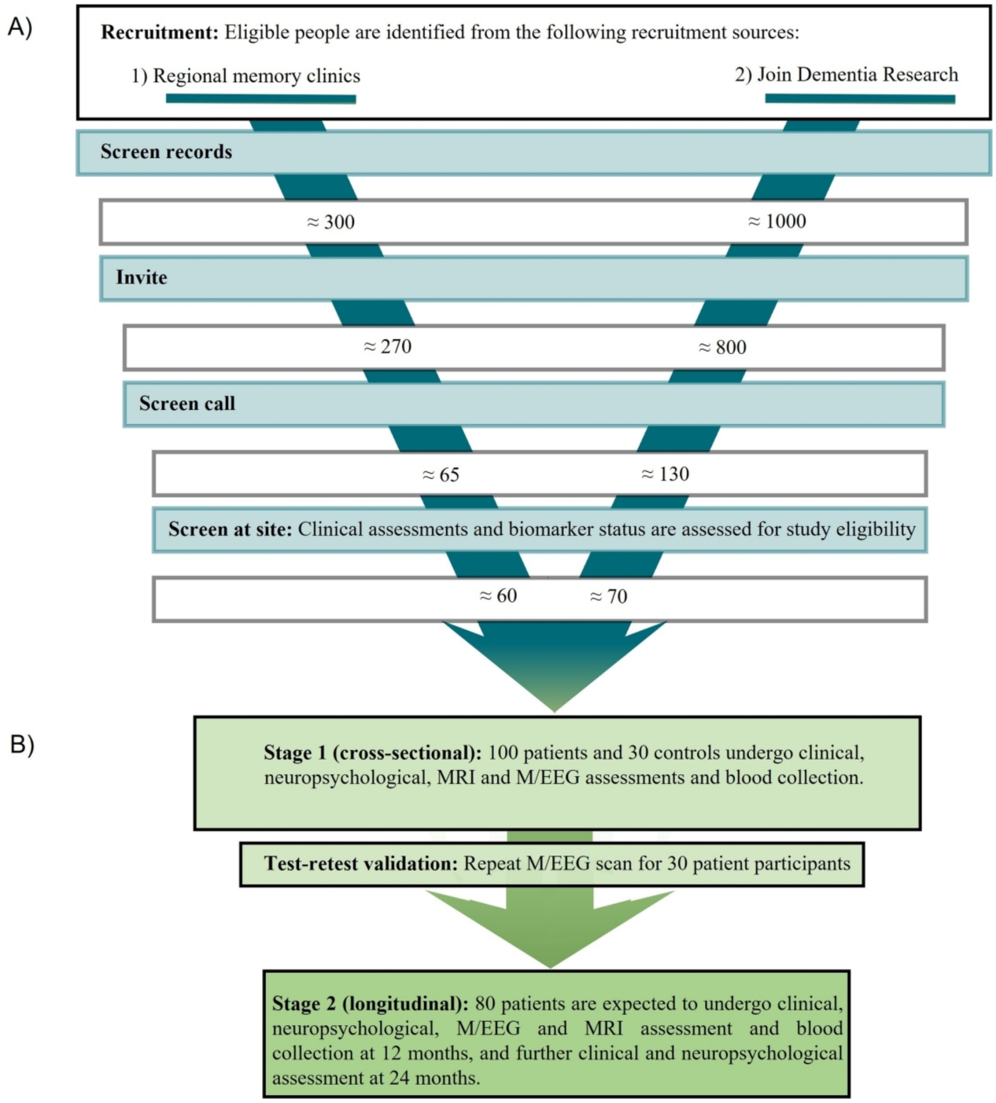

Participant flow chart. A) Recruitment strategy: Electronic screening of records from regional memory clinics and Join Dementia Research generates approximately 300 potential patient and 1000 potential patient and control participants who are invited to telephone screening. People who remain eligible are screened further on-site to identify 100 patient and 30 control participants. B) Study stages: Stage 1 consists of the cross-sectional baseline assessments and, for 30 patients, a repeat of the M/EEG assessment two weeks after the first. Only patients continue to the longitudinal stage 2 of the study, with repeat assessments 12 and 24 months after baseline. Attrition of 20% is expected at stage 2. M/EEG=Magnetoencephalography combined with electroencephalography imaging, MRI=Magnetic resonance imaging

170x188mm (300 x 300 DPI)

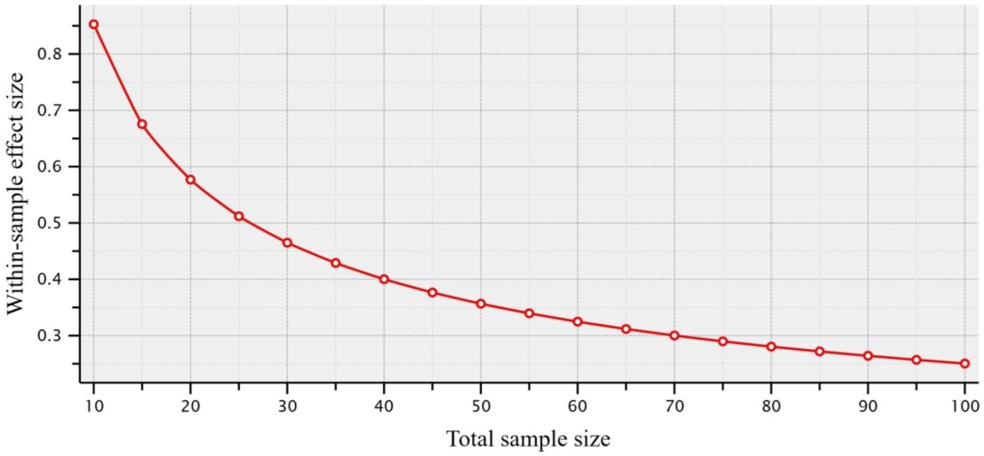

Effect sizes required to detect longitudinal correlations with disease severity at different attrition rates (resulting in reduced longitudinal sample size) with 80% power and α=0.05. We expect <20% attrition.

178x83mm (300 x 300 DPI)

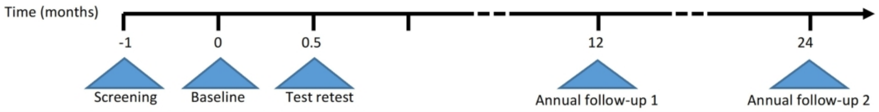

	Screening	Baseline	Test retest	Annual follow-up 1	Annual follow-up 2
Blood collection	X			X	
Biomarker samples	X				
Clinical assessments	X			X	X
Neuropsychological assessments		X		X	X
EEG and MEG scan		X	X	X	
MRI scan		X		X	

Timeline summary of study procedures

336x159mm (300 x 300 DPI)

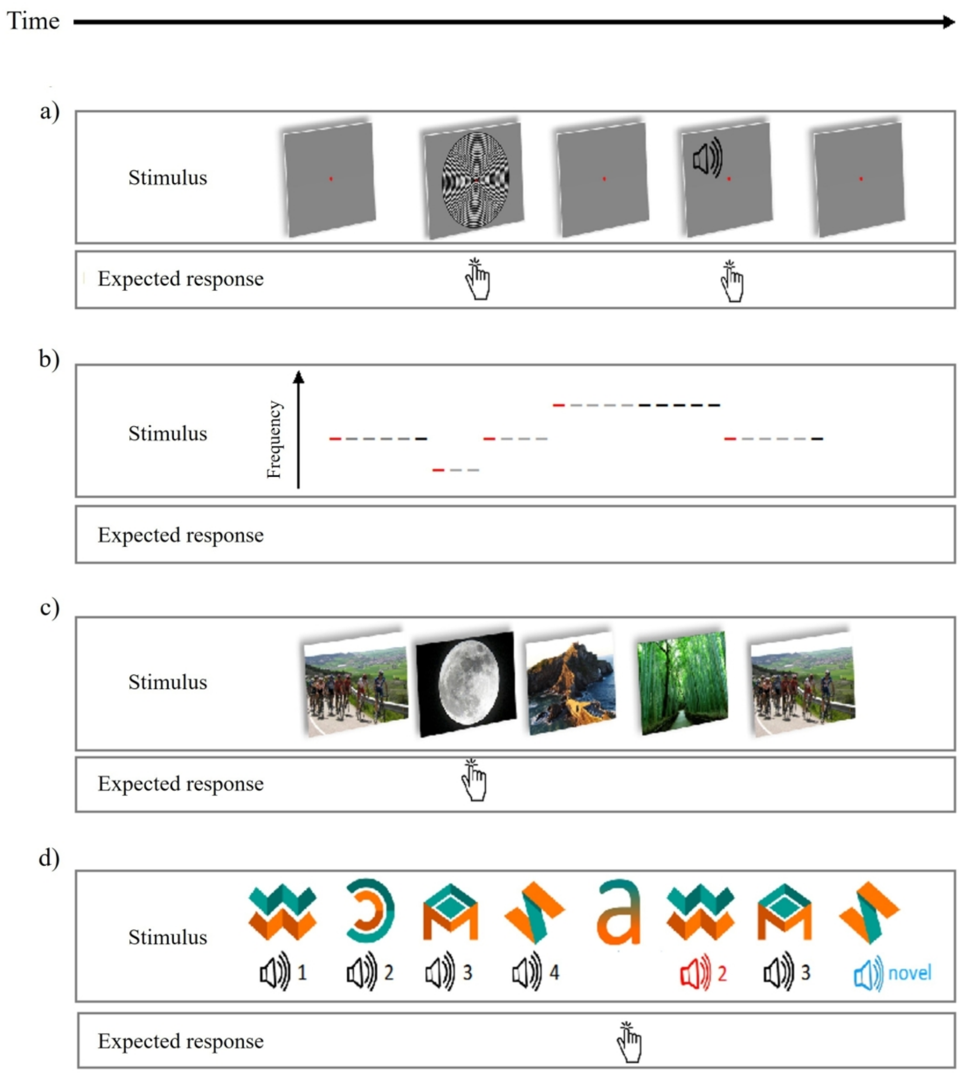

a) Audio-visual task: Participants visually fixate on a red dot and press a button whenever they hear or see something. (b) Mismatch-negativity task: Participants passively watch a nature documentary while listening to tones through the earpieces. Red dashes represent deviant tones and black dashes represent standard tones (from the sixth repetition). (c) Scene repetition task: Participants view scenes and press the button when they see a scene containing a moon. Each non-target scene repeats once during the task. (d) Cross-modal oddball task: Participants view object-tone pairs consisting of 4 learnt (standard) pairs (the standard sounds are black in the figure); associative-deviant, object-tone pairs (the associative deviant sound is coloured red in the figure); and standard objects presented with novel tones (the novel deviant tones are blue in the figure). Participants press a button when they see the letter ‘a’.

190x208mm (300 x 300 DPI)

STROBE Statement—Checklist of items that should be included in reports of ***cohort studies***
NB result and discussion sections are less relevant as this is for an observational protocol paper

	Item No	Recommendation	Page No
**Title and abstract**	1	(*a*) Indicate the study's design with a commonly used term in the title or the abstract	1
		(*b*) Provide in the abstract an informative and balanced summary of what was done and what was found	2
**Introduction**			
Background/rationale	2	Explain the scientific background and rationale for the investigation being reported	3
Objectives	3	State specific objectives, including any prespecified hypotheses	4
**Methods**			
Study design	4	Present key elements of study design early in the paper	5
Setting	5	Describe the setting, locations, and relevant dates, including periods of recruitment, exposure, follow-up, and data collection	7
Participants	6	(*a*) Give the eligibility criteria, and the sources and methods of selection of participants. Describe methods of follow-up<br>(*b*) For matched studies, give matching criteria and number of exposed and unexposed	9-15
Variables	7	Clearly define all outcomes, exposures, predictors, potential confounders, and effect modifiers. Give diagnostic criteria, if applicable	8-15
Data sources/ measurement	8*	For each variable of interest, give sources of data and details of methods of assessment (measurement). Describe comparability of assessment methods if there is more than one group	NA
Bias	9	Describe any efforts to address potential sources of bias	NA
Study size	10	Explain how the study size was arrived at	7
Quantitative variables	11	Explain how quantitative variables were handled in the analyses. If applicable, describe which groupings were chosen and why	4
Statistical methods	12	(*a*) Describe all statistical methods, including those used to control for confounding<br>(*b*) Describe any methods used to examine subgroups and interactions<br>(*c*) Explain how missing data were addressed<br>(*d*) If applicable, explain how loss to follow-up was addressed<br>(*e*) Describe any sensitivity analyses	4
**Results**			
Participants	13*	(a) Report numbers of individuals at each stage of study—eg numbers potentially eligible, examined for eligibility, confirmed eligible, included in the study, completing follow-up, and analysed<br>(b) Give reasons for non-participation at each stage<br>(c) Consider use of a flow diagram	Fig 1
Descriptive data	14*	(a) Give characteristics of study participants (eg demographic, clinical, social) and information on exposures and potential confounders<br>(b) Indicate number of participants with missing data for each variable of interest<br>(c) Summarise follow-up time (eg, average and total amount)	NA
Outcome data	15*	Report numbers of outcome events or summary measures over time	NA

For peer review only - http://bmjopen.bmj.com/site/about/guidelines.xhtml

Main results	16	(*a*) Give unadjusted estimates and, if applicable, confounder-adjusted estimates and their precision (eg, 95% confidence interval). Make clear which confounders were adjusted for and why they were included<br>(*b*) Report category boundaries when continuous variables were categorized<br>(*c*) If relevant, consider translating estimates of relative risk into absolute risk for a meaningful time period	NA
Other analyses	17	Report other analyses done—eg analyses of subgroups and interactions, and sensitivity analyses	NA
**Discussion**			
Key results	18	Summarise key results with reference to study objectives	NA
Limitations	19	Discuss limitations of the study, taking into account sources of potential bias or imprecision. Discuss both direction and magnitude of any potential bias	NA
Interpretation	20	Give a cautious overall interpretation of results considering objectives, limitations, multiplicity of analyses, results from similar studies, and other relevant evidence	NA
Generalisability	21	Discuss the generalisability (external validity) of the study results	NA
**Other information**			
Funding	22	Give the source of funding and the role of the funders for the present study and, if applicable, for the original study on which the present article is based	21

*Give information separately for exposed and unexposed groups.

Note: An Explanation and Elaboration article discusses each checklist item and gives methodological background and published examples of transparent reporting. The STROBE checklist is best used in conjunction with this article (freely available on the Web sites of PLoS Medicine at http://www.plosmedicine.org/, Annals of Internal Medicine at http://www.annals.org/, and Epidemiology at http://www.epidem.com/). Information on the STROBE Initiative is available at http://www.strobe-statement.org.

For peer review only

BMJ Open

The New Therapeutics in Alzheimer's Disease Longitudinal Cohort study (NTAD): study protocol

Journal:	*BMJ Open*
Manuscript ID	bmjopen-2021-055135.R2
Article Type:	Protocol
Date Submitted by the Author:	21-Sep-2022
Complete List of Authors:	Lanskey, Juliette; University of Cambridge, MRC Cognition and Brain Sciences Unit<br>Kocagoncu, Ece; University of Cambridge, Department of Clinical Neurosciences and Cambridge University Hospitals NHS Foundation Trust, Cambridge Biomedical Campus<br>Quinn, Andrew; University of Oxford, Oxford Centre for Human Brain Activity, Wellcome Centre for Integrative Neuroimaging, Department of Psychiatry<br>Cheng, Yun-Ju; Lilly Corporate Center<br>Karadag, Melek; University of Cambridge, Department of Clinical Neurosciences and Cambridge University Hospitals NHS Foundation Trust, Cambridge Biomedical Campus<br>Pitt, Jemma; University of Oxford, Oxford Centre for Human Brain Activity, Wellcome Centre for Integrative Neuroimaging, Department of Psychiatry<br>Isaac, John; Johnson and Johnson London Innovation Center, Neuroscience External Innovation<br>Lowe, Stephen; Lilly Corporate Center<br>Perkinton, Michael; AstraZeneca, Neuroscience, BioPharmaceuticals R&D<br>Raymont, Vanessa; University of Oxford, Department of Psychiatry<br>Singh, Krish; Cardiff University, Cardiff University Brain Research Imaging Centre, School of Psychology<br>Woolrich, Mark; University of Oxford, Oxford Centre for Human Brain Activity, Wellcome Centre for Integrative Neuroimaging, Department of Psychiatry<br>Nobre, Anna; University of Oxford, Oxford Centre for Human Brain Activity, Wellcome Centre for Integrative Neuroimaging, Department of Psychiatry<br>Henson, Richard; University of Cambridge, MRC Cognition and Brain Sciences Unit; University of Cambridge, Department of Psychiatry<br>Rowe, James; University of Cambridge, Department of Clinical Neurosciences and Cambridge University Hospitals NHS Foundation Trust, Cambridge Biomedical Campus; University of Cambridge, MRC Cognition and Brain Sciences Unit
**Primary Subject Heading**:	Neurology
Secondary Subject Heading:	Radiology and imaging, Pharmacology and therapeutics
Keywords:	Protocols & guidelines < HEALTH SERVICES ADMINISTRATION &

	MANAGEMENT, Dementia < NEUROLOGY, Neurophysiology < NEUROLOGY, NEUROPHYSIOLOGY, NEUROLOGY, RADIOLOGY & IMAGING

SCHOLARONE™
Manuscripts

The New Therapeutics in Alzheimer's Disease Longitudinal Cohort study (NTAD): study protocol

Juliette H Lanskey[1], Ece Kocagoncu[2], Andrew J Quinn[3], Yun-Ju Cheng[4], Melek Karadag[2], Jemma Pitt[3], John Isaac[5], Stephen Lowe[4], Michael Perkinton[6], Vanessa Raymont[7], Krish D Singh[8], Mark Woolrich[3], Anna C Nobre[3], Richard N Henson[1,9], James B Rowe[1,2] on behalf of the NTAD study group

Affiliations

1. MRC Cognition and Brain Sciences Unit, University of Cambridge, Cambridge, UK
2. Department of Clinical Neurosciences and Cambridge University Hospitals NHS Foundation Trust, Cambridge Biomedical Campus, Cambridge, UK
3. Oxford Centre for Human Brain Activity, Wellcome Centre for Integrative Neuroimaging, Department of Psychiatry, University of Oxford, Oxford, UK
4. Lilly Corporate Center, Indianapolis, USA
5. Neuroscience External Innovation, Johnson & Johnson Innovations, London, UK
6. Neuroscience, BioPharmaceuticals R&D, AstraZeneca, Cambridge, UK
7. Department of Psychiatry, University of Oxford, Oxford, UK
8. Cardiff University Brain Research Imaging Centre, School of Psychology, Cardiff University, Cardiff, UK
9. Department of Psychiatry, University of Cambridge, Cambridge, UK

Correspondence

Juliette Lanskey
MRC Cognition and Brain Sciences Unit,
15 Chaucer Road,
Cambridge, CB2 7EF, UK
Juliette.lanskey@mrc-cbu.cam.ac.uk

Words	4000
Figures	4
Tables	4

For peer review only

ABSTRACT

Introduction

With the pressing need to develop treatments that slow or stop the progression of Alzheimer's disease, new tools are needed to reduce clinical trial duration and validate new targets for human therapeutics. Such tools could be derived from neurophysiological measurements of disease.

Methods and Analysis

The New Therapeutics in Alzheimer's disease study (NTAD) aims to identify a biomarker set from magneto/electro-encephalography that is sensitive to disease and progression over one year. The study will recruit 100 people with amyloid-positive mild cognitive impairment or early-stage Alzheimer's disease and 30 healthy controls aged between 50 and 85 years. Measurements of the clinical, cognitive and imaging data (magnetoencephalography, electroencephalography and magnetic resonance imaging) of all participants will be taken at baseline. These measurements will be repeated after approximately one year on participants with Alzheimer's disease or mild cognitive impairment and clinical and cognitive assessment of these participants will be repeated again after approximately two years. To assess reliability of magneto/electro-encephalographic changes, a subset of 30 participants with mild cognitive impairment or early-stage Alzheimer's disease will also undergo repeat magneto/electro-encephalography two weeks after baseline. Baseline and longitudinal changes in neurophysiology are the primary analyses of interest. Additional outputs will include atrophy and cognitive change and estimated numbers needed to treat each arm of simulated clinical trials of a future disease modifying therapy.

Ethics and Data statement

The study has received a favourable opinion from the East of England Cambridge Central Research Ethics Committee (REC reference 18/EE/0042). Results will be disseminated through internal reports, peer-reviewed scientific journals, conference presentations, website publication, submission to regulatory authorities and other publications. Data will be made available via the Dementias Platform UK Data Portal on completion of initial analyses by the NTAD study group.

For peer review only

STRENGTHS AND LIMITATIONS OF THIS STUDY

Strengths:

- NTAD is a longitudinal, multicentre study of magneto/electro-encephalographic measures of Alzheimer's disease progression
- NTAD assesses the test-retest reliability of magneto/electro-encephalographic parameters
- All participants with early-stage Alzheimer's disease or mild cognitive impairment will be amyloid positive

Limitations:

- Attrition during follow up may limit inferences
- Recruitment is from volunteer panels and clinical services that may not reflect national diversity

For peer review only

INTRODUCTION

With 44 million people living worldwide with dementia, treatments to slow or stop disease progression are urgently needed. Alzheimer's disease is the most prevalent dementia but despite rapid advances in therapeutics within preclinical models,[1,2] clinical trials remain expensive, slow and challenging[3–5] with high-profile failures.[6] Significant bottlenecks exist in early-phase trials, where efficacy rates are low and costs rapidly escalate. To bridge the gap between animal models and the human disease, better tools are needed to quantify pathogenic and pathophysiological mechanisms in patients *in vivo* providing evidence to pursue or discontinue trials of a candidate treatment. These tools should be safe, scalable and able to support early-phase trials over a short duration and viable budget.

Brain imaging is widely used to diagnose dementia and measure pathology in clinical trials. There are diverse imaging methods with differential sensitivity to brain structure, chemistry, pathology and function. For example, magnetic resonance imaging (MRI) is commonly used to measure structural changes related to Alzheimer's disease,[7] such as entorhinal cortex[8] and hippocampus volumes.[9] Positron emission tomography (PET) can quantify and localise Alzheimer pathology through the use of ligands that bind to the aggregated tau protein,[10–14] beta-amyloid plaques,[15] synapses[16] and neuroinflammation.[17]

Loss of synapses and synaptic plasticity is an early feature of Alzheimer's disease.[18–20] Indeed, cognitive decline may be more directly related to loss of synapses or synaptic plasticity than to atrophy.[21,22] As synaptic changes occur early in Alzheimer's disease,[21] synaptic measures could be sensitive to the earliest precursors of cognitive involvement. This accords with preclinical models in transgenic mice, where network physiology and cognition are impaired before tangles or cell death.[23]

In contrast to preclinical models and post-mortem analysis, there are limited options to assess synaptic function in humans, *in vivo*. While PET imaging now offers ligands that indirectly quantify synaptic density, electroencephalography (EEG) and magnetoencephalography (MEG) measure neurophysiological properties dependent on synaptic integrity and function. MEG, for example, can identify synaptic and local circuit impairments[24,25] and their impact on network dynamics in Alzheimer's Disease,[26–29] frontotemporal dementia,[25,30–33] and Lewy-body disease.[34] MEG and EEG (M/EEG) therefore have potential to support and de-risk clinical trials of novel compounds. To enable M/EEG as viable biomarkers in clinical trials, one must assess their test-retest reliability.

For peer review only - http://bmjopen.bmj.com/site/about/guidelines.xhtml

The New Therapeutics for Alzheimer's Disease (NTAD) study aims to identify viable MEG and EEG biomarkers for clinical trials. NTAD is a multicentre study established by the Dementias Platform UK, and supported by the Medical Research Council, Alzheimer's Research UK and industry partners.

This paper describes the NTAD protocol to acquire longitudinal MEG and EEG data in people with biomarker-positive mild cognitive impairment or early Alzheimer's disease.

Research Aims

The primary objective is to identify a biomarker set from neurophysiological M/EEG sensitive to the presence and progression of Alzheimer's disease, in support of experimental medicine studies. We aim to harmonise M/EEG protocols across sites to allow biomarker identification from pooled data. Such a neurophysiological biomarker, or biomarker set, should be related to cognitive function, have high test-retest reliability and track disease progression over the duration of clinical trials. Sensitivity to disease progression should ideally outperform current widely used biomarkers such as MRI and cognitive tests. Our secondary objective is to identify a biomarker set that can predict disease progression, explain individual differences in the future disease trajectory and variation in cognitive decline over time. This paper describes the study design, including participants and methods for data acquisition, harmonisation and sharing.

Analysis

Investigators across sites will identify harmonised M/EEG pre-processing pipelines to reduce analytical variance. Following standardised pre-processing, investigators will address the study aims with multiple analytical approaches, which will be detailed in future, peer-reviewed papers. Where appropriate, investigators are encouraged to pre-register analyses. Analyses will follow an overarching framework:

Stage 0 (descriptive) analysis will describe demographics; APOE genotype; blood-based, fluidic biomarkers; neurocognitive profiles and regional brain atrophy. This serves to confirm the expected patterns of cognitive decline and atrophy typical of people with mild cognitive impairment and early Alzheimer's disease and to enable comparison of the NTAD cohort across sites and with other study populations.

Stage 1 (baseline) analyses will identify reliable and sensitive baseline, disease-related M/EEG parameters in terms of (i) the sensitivity of cross-sectional M/EEG parameters to group differences, (ii) the correlation of cross-sectional M/EEG parameters to baseline cognition, atrophy and fluidic markers and (iii) the test-retest reliability of M/EEG parameters.

Stage 2 (longitudinal) analyses will assess the sensitivity of selected neurophysiological markers to longitudinal change by assessing how they change over ~12 months. Sensitivity (as accuracy and effect sizes) to disease progression can be compared across modalities.

Stage 3 (predictive) analyses aim to identify baseline parameters that predict disease progression and identify the optimal parameter, or parameter set, that provides parsimonious or unique predictive information.

Potential parameters at stages 1-3 include: (i) summary statistics of evoked and induced responses for each task; (ii) summary statistics of the spectral density in resting-state recordings; (iii) data-driven connectivity metrics, including those derived from Hidden Markov Modelling[35–37] and (iv) parameters of model-based analyses of evoked responses

For peer review only

and spectral densities using dynamic causal modelling.[25,38,39] The dataset will be suitable for mediation modelling to determine how longitudinal changes in brain structure and neurophysiology explain cognitive changes. Full details of these analyses will be described in their respective pre-registration reports and peer-reviewed publications. With data sharing, new analytical methods may be applied to this dataset alone or in combination with other cohorts' data, e.g. ENIGMA-MEG (http://enigma.ini.usc.edu/ongoing/enigma-meg-working-group/) and BIOFIND (https://biofind-archive.mrc-cbu.cam.ac.uk/).

DNA will allow the description of genotype, including common genetic variants, and post-hoc identification of rare, autosomal dominant cases which may have distinctive physiology and phenotype. Potential analyses could examine the relationship between neurophysiological changes and (i) Alzheimer's disease risk alleles (e.g. apolipoprotein e4), (ii) polygenic risk scores of Alzheimer's disease and (iii) plasma tau levels; noting that the study is not designed for adequate power for genome-wide associations except as a contribution to pooled analyses. RNA is collected to enable post-hoc transcriptomics analysis.

METHODS

Study Design

This is a repeated-measures, observational-design study with 130 participants tested at two sites: Cambridge University and Oxford University, each in partnership with local NHS Hospital Trusts. The planned start and end dates are 01/06/2018 and 01/07/2023. The protocol includes two stages (Figure 1). The first, cross-sectional stage consists of baseline and test-retest sessions. Baseline sessions are comprised of clinical and neuropsychological assessments, an MRI scan and a M/EEG scan. For the test-retest session, a subset of patients returns for a second M/EEG approximately two weeks after the first. Patients proceed to stage 2, with repeat assessment at 12 months (including M/EEG, MRI, clinical and neuropsychology reassessments) and 24-months (clinical and neuropsychological reassessments).

Participant recruitment and selection

Participants are aged between 50 and 85 years with similar numbers of men and women, with symptomatic mild cognitive impairment or early Alzheimer's Disease (n=100) or normal cognition (n=30, Figure 1). Potential participants are identified by a staged screening process. First, approved clinical staff undertake electronic screening of records held by regional memory clinics or NIHR Join Dementia Research, or research team members review electronic records of people who previously consented to data retention on a contact list regarding dementia research opportunities. A participant information sheet detailing the study procedures is provided to candidate participants and a study partner who regularly sees the participant and is willing to complete assessments. Candidate participants answer a series of questions derived from the inclusion and exclusion criteria (Table 1) during a telephone-based screening call.

Potentially eligible participants are invited to on-site screening (see below). For those with Alzheimer's disease or mild cognitive impairment, clinical letters are sought from their memory assessment service, in addition to within-study assessments. This final screening appointment allows further assessment of eligibility, against exclusion and inclusion criteria

For peer review only

(Table 1) and Alzheimer biomarker status. Any uncertainty regarding eligibility is discussed with the principal investigator for the relevant site. Eligible people proceed to Stage 1.

Group inclusion criteria	
**Patients**	**Controls**
Diagnosis of MCI or AD#	No neurological diagnosis
CDR = 0.5 – 1	CDR = 0
Positive AD biomarker status (CSF or PET)	Known AD biomarker status (CSF or PET)
MMSE > 18	MMSE > 24
50-85 years	50-85 years
**General exclusion criteria (patients and controls)**	
Significant neurological disease, other than Alzheimer's disease, that may affect cognition or ability to complete the study	
Presence of any significant psychiatric disorder that could affect participation [40]	
Any clinically important abnormality that could compromise study participation	
A clinically significant illness, medical or surgical procedure, or trauma within 30 days prior to screening or baseline	
Known or suspected systemic infection	
Medications affecting cognition, unless on a stable dose for >30 days prior to baseline	
Rosen Modified Hachinski Ischaemic score ≥ 4	
History of seizure, except febrile seizures or single provoked seizure	
Head trauma resulting in protracted loss of consciousness, or serious infectious disease affecting the brain, within five years of screening and baseline	
Participation in a clinical trial of an investigational medicinal product	
Impairment of vision or hearing that could affect study participation	
Formal education ≤ 7 years	
Lack of mental capacity or other ability to consent	
Inability to read and write fluently in English	
Inability to walk 10 metres independently*	
Contraindications to blood sampling	
Contraindications to lumbar puncture (e.g. spinal deformations) and amyloid PET scan	
Contraindication to MRI (including, but not limited to, claustrophobia; pregnancy; MR-incompatible pacemakers and other MR-incompatible, implanted medical devices)	
Metallic implants in the body that affect MEG recordings, as judged by the Investigator	

Table 1 Group inclusion criteria and general exclusion criteria. *MCI=Mild Cognitive Impairment, AD=Alzheimer's Disease, MMSE=Mini Mental State Examination, CDR=Clinical Dementia Rating. # according to* [41] or [42] * *Ten metre walking is required for practicality and safety during the scanning sessions to avoid the use of walking aids, which may have metallic constituents.*

For peer review only

Sample Size and Power

Previously published studies have detected the presence and progression of Alzheimer's disease or mild cognitive impairment with under 100 participants using MRI.[43] We aim to identify MEG measures that are at least as sensitive as MRI. For longitudinal analysis, MEG[44–46] and EEG[47–52] have been used over 11-36 months with 20-130 participants. Mandal and colleagues[53] summarise the accuracies of several MEG measures to distinguish Alzheimer's disease from controls: 30/34 MEG measures had areas under the curve >0.64 approximating (under Gaussian assumptions) to Cohen's d>0.5.[54]

Power analyses were conducted in G*power3.1,[55] to determine effect sizes detectable with 80% power and 5% error rate. For the cross-sectional NTAD data with a group of 100 patients and 30 controls, a one-tailed, independent t-test can detect medium effect sizes d>0.52, while one-tailed correlations with disease severity in the patient group can detect effect sizes of R>0.24. Assuming 20% attrition at follow up (n=80), longitudinal analyses of disease progression in the patient group would have 80% power to detect within-sample differences of d>0.28 (Figure 2). Future pre-registration and analysis reports will undertake specific power estimations.

Patient and public involvement

The use and tolerability of M/EEG in combination with the PET or CSF, MRI and neuropsychology was discussed as part of Alzheimer Society sponsored patient groups with patients and carers affected by AD or MCI for the closely related Dementias Platform UK study known as 'Deep and frequent phenotyping' with which NTAD is aligned in design and methods. In addition, the use of MEG for dementia research featured in the public engagement event 'MEG and me' as part of the Cambridge Science Festival and has been discussed at open meetings at the Cambridge Science Festival and Alzheimer Research UK Eastern Region network public meetings. The study is also co-funded by Alzheimer Research UK, a charity with a joint patient advocacy and research funding remit.

STUDY PROCEDURES

Overview of protocol stages

Screening

Potential participants are identified from electronic records and screened further via telephone call. Prospective participants who remain eligible are invited to an in-person screening appointment. Any further questions they have are answered before they provide written, informed consent. After written, informed consent, a medically-qualified clinician administers the clinical interview, clinical dementia rating and Haschinski ischemic scale. In the context of this study, medically qualified means having at least MRCP part II qualifications and in ST3+ level neurology or psychiatry training, or completion of neurology or psychiatry specialist training. The remaining clinical assessments are completed by a member of the research team and include physiology and blood sampling. All research team members have current Good Clinical Practice training. If Alzheimer biomarker status is unknown from recent clinical or research assessments, participants proceed to either cerebrospinal fluid examination or amyloid PET imaging according to participant preference and eligibility. For patients, where a participant's positive amyloid status has been confirmed previously, a positive result enables participation, a negative result excludes participation.

For peer review only

For controls, the Alzheimer biomarker status must be known to proceed but, given their CDR=0, a positive test does not prevent participation.

Stage 1: Baseline
One hundred people with mild cognitive impairment or Alzheimer's disease and 30 neurologically normal people proceed to baseline assessment. Participants undergo structured neuropsychological assessment, M/EEG and MRI over two sessions (or three by preference).

Stage 1: Two-week M/EEG retest
We invite 30 people from the patient group (i.e. mild cognitive impairment or Alzheimer's disease) to repeat the M/EEG scan between two and four weeks after the first scan. Invitations are prioritised to people who can most readily attend the additional session (e.g. considering distance) until the target sample size is reached.

Stage 2: Annual follow-up 1
Participants in the patient group repeat the clinical and neuropsychological assessments, M/EEG scan, MRI scan and blood collection at 12 months after baseline.

Stage 2: Annual follow-up 2
Clinical and neuropsychological assessments are repeated at 24 months for participants in the patient group.

The study timeline is illustrated in Figure 3.

Blood Samples

Participants are asked to consume only water for two hours prior to blood collection. At baseline and follow-up, blood is drawn in the following order: 2.7ml in Sodium Citrate Tubes (plasma), 5ml in Serum Separator Tubes (serum), 10ml in EDTA tubes (DNA), 10ml in EDTA tubes (plasma and buffy coat) and 2x2.5ml in PAXgene tubes (RNA). Once filled, the EDTA and PAXgene tubes are inverted 10 times. From April 2021, 4.9ml of blood is collected in S-monovette tubes for SARS-CoV-2 serology. Samples are processed and frozen according to the guidelines in Table 2.

**Plasma**	The sample in the sodium citrate tube is centrifuged at 2000g for 10 minutes at room temperature. The plasma is aliquoted into 1.4 ml matrix tubes and stored at -80°C.
**Serum**	At least 30 minutes after blood collection, the sample in the serum separator tube is centrifuged at 2000g for 10 min at room temperature. The serum is aliquoted into 1.4ml matrix tubes and stored at -80°C.<br><br>Similarly, the sample in the S-monovette tube is centrifuged at 2000g for 10 minutes at room temperature at least 30 minutes after collection. The sample is aliquoted into 2ml Sarstedt tubes.
**DNA**	The first EDTA tube is frozen as soon as possible and stored at -80°C for later DNA extraction and analysis.
**Plasma and buffy coat**	The second EDTA tube is centrifuged immediately at 2500g for 15 minutes at room temperature. The plasma and buffy coat are aliquoted into separate 1.4 ml matrix tubes and stored at -80°C.
**RNA**	Paxgene tubes are stored at room temperature for 2 hours before being frozen upright at -20°C for 24 hours and then stored at -80°C.

Table 2 Blood sample processing guidelines

For peer review only - http://bmjopen.bmj.com/site/about/guidelines.xhtml

Biomarkers for Alzheimer disease pathology

Clinical criteria are insufficient to reliably diagnose the presence of Alzheimer's disease pathology.[56,57] We seek additional biomarker evidence, using either cerebrospinal fluid or PET imaging according to participant preference and eligibility.[42]

Cerebrospinal fluid

Cerebrospinal fluid is obtained by lumbar puncture and collected in polypropylene tubes. Within one hour of collection, cerebrospinal fluid is centrifuged, separated and the supernatant frozen to −80°C for later batched analysis using a chemiluminescent enzyme immunoassay for total tau, phosphorylated tau and amyloid beta 1-42 levels. Positive amyloid status is indicated by a total tau to amyloid beta 1-42 ratio >1, and amyloid beta 1-42 concentration <450pg/ml. pTau181 concentration is recorded from April 2021.

Amyloid PET

Participants receive a 300MBq bolus injection of florbetaben and are scanned 80-100 minutes post-injection on a GE Signa PET/MR scanner at the Wolfson Brain Imaging Centre, Cambridge or a GE D710 PET/CT scanner at the Churchill Hospital, Oxford. The centiloid method is used to classify the florbetaben scans as amyloid positive with centiloid >1.19.[58,59]

Clinical Assessments

Clinical assessments are completed at a clinical research centre in one or two sessions.

Clinical Interview

A study clinician interviews the participant and study partner. Clinicians follow a structured interview that covers socio-demographic factors, including age and years of education; lifestyle factors; family history of dementia; medical history, including information on significant medical conditions with date of onset; and concomitant medication usage, with details about dosage and duration.

Addenbrooke's Cognitive Examination

The revised Addenbrooke's Cognitive Examination evaluates orientation, memory, verbal fluency, language and visuo-spatial domains.[60] It is administered and scored according to the Administration and Scoring Guide (2006). Alternate versions at each visit reduce practice effects.

Mini-Mental State Examination

The Mini-Mental State Examination is acquired.[61]

Clinical Dementia Rating

The Clinical Dementia Rating quantifies dementia severity through a structured interview.[62–64] Interviewers rate participant impairments in 6 categories: memory, orientation, judgment and problem solving, community affairs, home and hobbies and personal care. The global CDR is derived from these ratings.[63]

Haschinski Ischaemic Score

For peer review only

The Rosen modification of Haschinski's Ischaemic Score seeks to differentiate between primary progressive dementias, including Alzheimer's disease and multi-infarct dementia.[65] A study clinician uses information from medical history, physical and neurological examination and medical records to determine the score. Scores below 4 indicate a low likelihood of vascular disease as the cause of dementia.

Self-reported questionnaires

Participants complete the 30-item Geriatric Depression Scale,[66] 40-item Spielberger State-Trait Anxiety Inventory,[67] and 11-item Pittsburgh Sleep Quality Index.[68] Study partners complete the 30-item Amsterdam Instrumental Activity of Daily Living Questionnaire (short version)[69] and Mild Behavioral Impairment Checklist.[70]

Physiological measures

Height, weight, hip-waist ratio, blood pressure, pulse rate and temperature are measured for participants using a stadiometer, electronic weight scales and stretch-resistant tape.

Neuropsychological Assessment

For peer review only

The neuropsychological test battery closely resembles the Deep and Frequent Phenotyping study[71] and IMI-European Prevention of Alzheimer's Disease,[72,73] including the Pre-Alzheimer Cognitive Composite.[74] Assessments take place in a private, testing room in one session with breaks as needed, but two breaks by default (see Table 3).

Order of assessments	Duration (minutes)
1. List learning (RBANS subtest)<br>2. Story memory (RBANS subtest)<br>3. Figure Copy (RBANS subtest)<br>4. Line orientation (RBANS subtest)<br>5. Picture naming (RBANS subtest)<br>6. Semantic fluency (RBANS subtest)<br>7. Digit span forwards (RBANS subtest)<br>8. Digit span backwards<br>9. Coding (RBANS subtest)<br>10. List recall (RBANS subtest)<br>11. List recognition (RBANS subtest)<br>12. Story recall (RBANS subtest)<br>13. Figure recall (RBANS subtest)	≈ 40
Break	≈ 15
14. Free and cued selective reminding test (PACC)<br>15. Logical memory part 1 (PACC)<br>16. National adult reading test<br>17. Digit symbol substitution (PACC)<br>18. Trails B<br>19. Logical memory part 2 (PACC)	≈ 45
Break	≈ 15
20. Reaction time task (CANTAB subtest)<br>21. Paired associates learning (CANTAB subtest)<br>22. Rapid visual processing (CANTAB subtest)<br>23. Spatial working memory (CANTAB subtest)<br>24. Four mountains task	≈ 30

Table 3 Order of neuropsychological assessments. *RBANS=Repeatable Battery for the Assessment of Neuropsychological Status, PACC=Pre-Alzheimer Cognitive Composite, CANTAB = Cambridge Neuropsychological Test Automated Battery*

Repeatable Battery for the Assessment of Neuropsychological Status (RBANS)

RBANS measures cognitive decline in 5 domains: immediate memory, visuospatial, language, attention and delayed memory.[75] Participants receive alternate forms at repeated assessments. The immediate memory index comprises the *list learning subtest*, with immediate recall of 10-items over 4 trials and the *story memory subtest*, with immediate recall of a 12-item story over two trials. The visuospatial index comprises the *figure copy subtest*, which involves copying a geometric figure and the 10-item, *line orientation subtest*. The language index comprises the 10-item, *picture naming subtest* and the *semantic fluency subtest*, where participants name as many exemplars of the given semantic category as

possible in 60 seconds. The attention index comprises the *digit span forwards subtest*, involving immediate repetition of increasing digit strings and the *coding subtest*, scored as the total number of correctly coded numbers generated using an item-to-number code within 90 seconds. The delayed memory index comprises the *list recall subtest*, involving free recall of the list learning task; *list recognition*, where participants decide whether a word was included in the list learning task; *story recall*, where participants freely recall the story memory task; and *figure recall*, where participants draw from memory the figure presented in the figure copy task.

Digit Span Backwards

Digit span backwards, from the Wechsler adult intelligent scale,[76] is used in conjunction with RBANS digit span forwards. It follows the RBANS digit span format but participants are asked to immediately repeat in reverse order.

Free and Cued Selective Reminding Test

The Free and Cued Selective Reminding Test assesses episodic memory and distinguishes retrieval from storage deficits.[77] Sixteen pictured items are encoded during the initial learning phase, where participants identify and name items responding to unique semantic cues. After a short delay, participants freely recall all items. Interviewers prompt for each item not recalled using the unique semantic cues from the learning phase. Participants receive alternate forms at repeated assessments.

Logical Memory

Logical Memory, taken from the Wechsler memory scale (third edition), assesses episodic memory.[78] Participants immediately recall short stories they have been read. After a 30-minute delay with intervening tests, participants freely recall the stories and answer yes or no questions testing story recognition.

National Adult Reading Test

The national adult reading test (second edition) estimates premorbid intelligence from printed, irregular words.[79]

Digit Symbol Substitution

The digit symbol substitution from the Wechsler Adult Intelligence Scale assesses processing speed and attention.[76] Participants have 90 seconds to code as many correct symbols as possible corresponding to presented numbers by using the given number-to-symbol code.

Trails Making Test B

The Trails Making Test B assesses executive function, attention and processing speed.[80] Following a practice sample to ensure task comprehension, participants are presented with 25 encircled numbers on a page which they connect by alternating between numbers and letters. The time it takes to complete the sample and any errors made are recorded.

Cambridge Neuropsychological Test Automated Battery

The tablet-based Cambridge Neuropsychological Test Automated Battery assesses processing speed, episodic memory, attention, working memory and executive function.[81] The reaction time task (simple and five choice variant) assesses processing speed. Participants hold down a response button and release this to respond to on-screen targets. The paired-associates learning task (standard variant) assesses episodic memory. Participants learn

For peer review only

associations between patterns and their locations. An initial learning stage precedes immediate recall. The rapid visual processing task (3 target variant) assesses attention. Single digits appear on the screen. Participants respond when they see a string matching target sequences. The spatial working memory task (standard variant) assesses working memory and executive function. Participants search inside on-screen boxes to find and collect tokens. Tokens never appear in the same box twice.

Four Mountains Task

The tablet-based four mountains task assesses allocentric spatial processing.[82] During a learning phase, participants learn the topographical layout of 4 mountains presented in a computer-generated landscape. Following a delay, participants are presented with four alternative images and identify the target image matching the topographical mountain layout of the learning-phase image, but with potentially altered colours, textures and points of view.

Neurophysiology (M/EEG)

M/EEG data are collected simultaneously at 1000Hz in a magnetically-shielded room. At Cambridge, data are collected using the Elekta VectorView system from 2017 to December 2019, with 204 planar gradiometers, 102 magnetometers and an 70-channel Easycap EEG. Stage 1 scans and the first 12 follow-up scans used the same scanner. The MEGIN Triux Neo M/EEG scanner is used from March 2020 onwards with the same sensor configuration as the VectorView and a 64-channel Easycap. At Oxford, the MEGIN Triux Neo M/EEG scanner and an EasyCap 60 channel BrainCap for MEG with an augmented 10/20 layout are used for all data collection.

The Polhemus digitisation system records the position of the standard fiducial points, >300 additional head points, five head position indicator coils and EEG electrodes. Head position indicator coils measure head position within the MEG helmet. Electrodes on the right clavicle and left lower rib record electrocardiogram data. Electrodes above and below the left eye and on bilateral canthi record vertical and horizontal electrooculogram data. Reference and ground electrodes are placed on the left side of the nose and left cheek, respectively. During the seated scan, participants rest or perform simple tasks, responding through a button box. Participants wear non-magnetic earphones with sound delivered though plastic tubes and, if necessary, non-magnetic glasses. Prior to M/EEG, a Snellen eye test and pure tone audiometry assess sight and hearing thresholds. Tasks are performed in the following order.

Simple audio-visual task

Participants fixate on a red, central fixation dot and quickly respond to each auditory or visual stimuli with a button press (Figure 4a). Auditory tones (n=100) of 300Hz, 600Hz or 1200Hz are presented for 300ms after a blank interval of 1000ms. Visual stimuli are concentric black and white circles that appear for 300ms after 3000ms. Filler trials (n=30) containing only the red fixation dot are included. Visual and auditory trials are randomly intermixed. Ten initial practice trials familiarise participants to the task.

Auditory Mismatch-negativity Task

The roving auditory mismatch negativity task elicits error responses to deviant tones followed by rapid plasticity as predictions are updated upon repetition of the deviant stimulus.[83,84] Participants passively watch a muted nature documentary. Through earpieces, they hear binaural, in-phase sinusoidal tones >60dB above the average auditory threshold with durations of 100ms and 500ms stimulus onset asynchrony. Tone frequencies

For peer review only

are the same within but different between blocks. Blocks range from 400 to 800Hz. The number of tones per blocks varies from 3 to 11 according to a truncated exponential distribution (Figure 4b).

Scene-Repetition Task

In this passive memory test, participants view a series of complex scenes (landscapes and cityscapes) and press a button only when scenes contain a moon (n=26, different moon images). Target scenes containing a moon ensure attention but are not of interest; the main interest is differences between initial and repeated presentations of non-target scenes. Each scene is presented for 800ms and preceded by a fixation cross of 200ms on average (100-300ms). Scenes are pseudo-randomly intermixed with the constraint that 10 initial 'burn-in' scenes, with 2 moon target scenes, precede 256 scenes presented twice, with 14-93 (median=42) intervening scenes between the first and repeat presentation (Figure 4c).

Cross-modal Oddball Task

The task assesses hippocampal-dependent, paired-associates learning.[85,86] Trials comprise of a 700ms visual, abstract "object" and a 400ms sound, starting 300ms after trial onset. Inter-stimulus intervals average 300ms. During the initial training period (80 trials), participants learn associations between four standard pairs of visual objects and sounds. The main task has 770 bimodal trials and 40 unimodal trials, randomly intermixed. Bimodal trials consist of: standard, learnt object-sound pairs (n=670); standard objects paired with novel sounds (n=50); and mismatched pairs, where the object and sound are from different standard pairs (n=50). Unimodal trials ensure the task is attended, participants press a button when they see the letter 'a' (Figure 4d). A 10-item assessment follows the task, participants hear a standard sound and report which of four presented objects the sound was paired with most often during the task.

Eyes-open Resting State

Participants are presented with a central fixation cross and instructed to clear their mind, relax, think of nothing specific and focus on the fixation cross for 5 minutes.

Eyes-closed Resting State

Participants are instructed to clear their mind, relax, think of nothing specific and close their eyes but stay awake.

Magnetic Resonance Imaging

MRI sequences use 3T Siemens PRISMA scanners at the Cambridge MRC Cognition and Brain Science Unit and Oxford Centre for Human Brain Activity (Table 4).

**T1-weighted**	The 3D T1-weighted structural image is acquired using the Generalized Autocalibrating Partially Parallel Acquisitions (GRAPPA) technique applied to a high-resolution Magnetization Prepared Rapid Gradient Echo (MPRAGE) sequence with the following parameters: TE=2.91ms, TR = 2300.00ms, TI=900.00ms, flip angle =9 degrees, acquisition matrix =256 x 240, voxel size =1.00mm isotropic, number of slices =176, slice thickness =1mm, acquisition time =5 minutes and 12 seconds
**T2 FLAIR**	The 3D FLAIR structural image is acquired using the following parameters: TE=394.00ms, TR =5000.00ms, TI =1650.00ms, flip angle

	=120 degrees, acquisition matrix =256 x 256, voxel size =1.00mm isotropic, number of slices =192, slice thickness =1.00mm, acquisition time =5 minutes and 7 seconds
**T2*-weighted**	The T2*-weighted structural image is acquired using the following parameters: TE =20.00ms, TR =640.00ms, Flip angle =20 degrees, acquisition matrix =256 x 256, number of slices =47, slice thickness =3.00mm, Voxel size =0.90x0.90x3.00mm, Acquisition time =4 minutes and 7 seconds
**T2-weighted with fat saturation**	The T2-weighted image with fat saturation is acquired with the following parameters: TE =78.00ms, TR =4000.00ms, flip angle =150 degrees, acquisition matrix =256x232, number of slices =47, slice thickness=3.00mm, voxel size =0.90x0.90x3.00mm, acquisition time =3 minutes and 26 seconds
**Diffusion Weighted Imaging**	The Diffusion-Weighted Images are acquired with a spin-echo echo-planar sequence. B-values of 0, 300, 700 and 2000 s/mm^2 were used with 116 diffusion gradient directions obtained via MRTrix3.[87] The number of signal averages was 12, 8, 32, and 64 for b-values of 0, 300, 700 and 2000, respectively. The acquisition is run in the transverse orientation without any angulation. Other scan parameters include: TR=3800ms; TE=85.00ms, voxel size=2.50mm isotropic, acquisition matrix =96x96, number of slices=64, slice thickness =2.50mm, acquisition time=8 minutes and 45 seconds
**Quantitative Susceptibility Mapping**	The quantitative susceptibility mapping correlates with Aβ-amyloid iron density and is acquired with a 3D Gradient Echo sequence with multiple echoes and flow compensation in the readout and slice directions. (TE 1=5.20ms, TE 2=10.4ms, TE 3 =15.6ms, TE 4 =20.8ms, TE 5=26.0ms; TR =31.0ms, flip angle =15 degrees, acquisition matrix =256x192, number of slices =160 (per TE), slice thickness =1.00mm, voxel size =1.00mm isotropic, acquisition time =5 minutes 10 seconds.
**Resting state eyes open**	For the resting-state scan, the participants are presented with a fixation cross and asked to maintain fixation throughout the task. Echo planar images are acquired with 200 volumes with the following parameters: TE=30.00ms, TR=1500.00ms, flip angle =90 degrees, acquisition matrix =64x64, number of slices =54, slice thickness =3.00mm, voxel size = 3.00mm isotropic, acquisition time =5 minutes 50 seconds
**Hippocampal subfields**	The high-resolution, hippocampal subfield images are acquired with T2-weighted, turbo spin echo sequences and run with the following parameters: TE=50.00ms, TR=8020.00ms, flip angle =122 degrees, acquisition matrix =448x448, number of slices =30, slice thickness =2.00mm, voxel size =0.4x0.4x2.0mm, acquisition time =8 minutes 11 seconds.
**Arterial Spin Labelling**	3D Arterial Spin Labelling images are acquired using a flow-sensitive alternating inversion recovery spin-echo pulsed sequence with Q2-TIPS bolus saturation in the transverse plane with the following parameters: TR=4000.00ms; TE =13.20ms; flip angle =130 degrees, labelling duration =800ms, post-labelling delay =2000ms, acquisition matrix =64x60; slices

For peer review only

	per slab =32, slice thickness =4.50mm, voxel size =1.85x1.85x4.50mm, acquisition time =6 minutes 46 seconds.

Table 4 Order and parameters of MRI sequences. *FLAIR=Fluid Attenuated Inversion Recovery, TE=Echo time, TR=Repetition time, TI=Inversion time*

CURRENT STATUS

The study is active at both sites.

ETHICS AND DISSEMINATION

The study received a favourable opinion from the East of England – Cambridge Central Research Ethics Committee (REC reference 18/EE/0042). Imaging data and clinical scores are hosted by Dementias Platform UK Imaging Platform (https://portal.dementiasplatform.uk), using XNAT (https://www.xnat.org). Data will be made available with a managed access process through Dementias Platform UK, subject to requesters agreeing to a Code of Conduct to preserve data security, confidentiality and privacy.

For peer review only

Figure Legends

Figure 1 Participant flow chart. A) Recruitment strategy: Electronic screening of records from regional memory clinics and Join Dementia Research generates approximately 300 potential patient and 1000 potential patient and control participants who are invited to telephone screening. People who remain eligible are screened further on-site to identify 100 patient and 30 control participants. B) Study stages: Stage 1 consists of the cross-sectional baseline assessments and, for 30 patients, a repeat of the M/EEG assessment two weeks after the first. Only patients continue to the longitudinal stage 2 of the study, with repeat assessments 12 and 24 months after baseline. Attrition of 20% is expected at stage 2. M/EEG=Magnetoencephalography combined with electroencephalography imaging, MRI=Magnetic resonance imaging

Figure 2 Effect sizes required to detect longitudinal correlations with disease severity at different attrition rates (resulting in reduced longitudinal sample size) with 80% power and α=0.05. Effect sizes were estimated using G*power3.1 software for one-tailed, paired t-tests. We anticipate <20% attrition per annum.

Figure 3 Timeline summary of study procedures

Figure 4 a) Audio-visual task: Participants visually fixate on a red dot and press a button whenever they hear or see something. (b) Mismatch-negativity task: Participants passively watch a nature documentary while listening to tones through the earpieces. Red dashes represent deviant tones and black dashes represent standard tones (from the sixth repetition). (c) Scene repetition task: Participants view scenes and press the button when they see a scene containing a moon. Each non-target scene repeats once during the task. Images in the figure are for illustrative purposes only. (d) Cross-modal oddball task: Participants view object-tone pairs consisting of 4 learnt (standard) pairs (the standard sounds are black in the figure); associative-deviant, object-tone pairs (the associative deviant sound is coloured red in the figure); and standard objects presented with novel tones (the novel deviant tones are blue in the figure). Participants press a button when they see the letter ‘a’. The visual objects were designed by Freepik.

For peer review only

FOOTNOTES

Author statement: JHL and JBR drafted the manuscript, with review and contribution from EK, AJQ, YC, MK, JP, JI, SL, MP, VR, KDS, MW, ACN and RNH. JBR is the chief investigator of the study. RNH, ACN, MW, KDS and VR are principal investigators, contributing to conception and design. EK led protocol development and governance. JBR, MW, ACN, RNH, JI, SL, MP and KDS conceived and designed the study.

Funding: This work is part of the Dementias Platform UK (MR/L023784/1 & MR/L023784/2) which is funded by the Medical Research Council, Janssen, AstraZeneca, Araclon, IXICO, Somalogic, GlaxoSmithKline, Invicro, Cambridge Cognition and Cognetivity. The study has additional support from Alzheimer's Research UK (ARUK-PG2017B-19), the Wellcome Trust (103838), Medical Research Council (SUAG/051 G101400; SUAG/046 G101400), NIHR Cambridge Biomedical Research Centre (BRC-1215-20014) and NIHR Oxford Biomedical Research Centre (BRC-1215-20008). The views expressed are those of the authors and not necessarily those of the NIHR or the Department of Health and Social Care. For the purpose of Open Access, the author has applied a CC BY public copyright licence to any Author Accepted Manuscript version arising from this submission.

Conflicts of interests: John Isaac is employed by Johnson & Johnson and may currently hold Johnson & Johnson stocks. Michael Perkinton is employed by AstraZeneca and may currently hold AstraZeneca stocks or stock options. Yun-Ju Cheng and Stephen Lowe are employed by Eli Lilly and may currently hold Eli Lilly stock. Mark Woolrich receives royalties from FSL and is senior editor for Neuroimage. James Rowe is Chief Scientific Adviser for Alzheimer Research UK and is a trustee of Brain, PSP association and Darwin college.

REFERENCES

1 LaFerla FM, Green KN. Animal models of Alzheimer disease. *Cold Spring Harb Perspect Med* 2012;**2**:1–13. doi:10.1101/cshperspect.a006320

2 Drummond E, Wisniewski T. Alzheimer's disease: experimental models and reality. Acta Neuropathol. 2017;**133**:155–75. doi:10.1007/s00401-016-1662-x

3 Cummings JL, Morstorf T, Zhong K. Alzheimer's disease drug-development pipeline: Few candidates, frequent failures. *Alzheimer's Res Ther* 2014;**6**:1–7. doi:10.1186/alzrt269

4 Yiannopoulou KG, Anastasiou AI, Zachariou V, *et al.* Reasons for failed trials of disease-modifying treatments for alzheimer disease and their contribution in recent research. Biomedicines. 2019;**7**. doi:10.3390/biomedicines7040097

5 Anderson RM, Hadjichrysanthou C, Evans S, *et al.* Why do so many clinical trials of therapies for Alzheimer's disease fail? Lancet. 2017;**390**:2327–9. doi:10.1016/S0140-6736(17)32399-1

6 Mehta D, Jackson R, Paul G, *et al.* Why do trials for Alzheimer's disease drugs keep failing? *Expert Opin Investig Drugs* 2017;**26**:735–9. doi:10.1080/13543784.2017.1323868.Why

7 Frisoni GB, Fox NC, Jack CR, *et al.* The clinical use of structural MRI in Alzheimer disease. *Nat Rev Neurol* 2010;**6**:67–77. doi:10.1038/nrneurol.2009.215

8 Holland D, McEvoy LK, Dale AM. Unbiased comparison of sample size estimates

For peer review only

from longitudinal structural measures in ADNI. *Hum Brain Mapp* 2012;**33**:2586–602. doi:10.1002/hbm.21386

9 Leung KK, Barnes J, Ridgway GR, *et al.* Automated cross-sectional and longitudinal hippocampal volume measurement in mild cognitive impairment and Alzheimer's disease. *Neuroimage* 2010;**51**:1345–59. doi:10.1016/j.neuroimage.2010.03.018

10 Cope TE, Rittman T, Borchert RJ, *et al.* Tau burden and the functional connectome in Alzheimer's disease and progressive supranuclear palsy. *Brain* 2018;**141**:550–67. doi:10.1093/brain/awx347

11 Passamonti L, Rodríguez PV, Hong YT, *et al.* PK11195 binding in Alzheimer disease and progressive supranuclear palsy. *Neurology* 2018;**90**:e1989–96. doi:10.1212/WNL.0000000000005610

12 Passamonti L, Rodríguez PV, Hong YT, *et al.* 18F-AV-1451 positron emission tomography in Alzheimer's disease and progressive supranuclear palsy. *Brain* 2017;**140**:781–91. doi:10.1093/brain/aww340

13 Leuzy A, Chiotis K, Lemoine L, *et al.* Tau PET imaging in neurodegenerative tauopathies—still a challenge. Mol. Psychiatry. 2019;**24**:1112–34. doi:10.1038/s41380-018-0342-8

14 Ossenkoppele R, Schonhaut DR, Schö M, *et al.* Tau PET patterns mirror clinical and neuroanatomical variability in Alzheimer's disease. *Brain* 2016;**139**:1551–67. doi:10.1093/brain/aww041

15 Cai L, Innis R, Pike V. Radioligand Development for PET Imaging of beta-Amyloid (AD)-Current Status. *Curr Med Chem* 2006;**14**:19–52. doi:10.2174/092986707779313471

16 Venkataraman A V, Mansur A, Rizzo G, *et al.* Widespread cell stress and mitochondrial dysfunction in early Alzheimer's Disease. *medRxiv* 2021;**1051**:2021.08.11.21261851.http://medrxiv.org/content/early/2021/08/13/2021.08.11.21261851.abstract

17 Bevan-Jones WR, Surendranathan A, Passamonti L, *et al.* Neuroimaging of inflammation in memory and related other disorders (NIMROD) study protocol: A deep phenotyping cohort study of the role of brain inflammation in dementia, depression and other neurological illnesses. *BMJ Open* 2017;**7**. doi:10.1136/bmjopen-2016-013187

18 Koffie RM, Hyman BT, Spires-Jones TL. Alzheimer's disease: Synapses gone cold. Mol. Neurodegener. 2011;**6**:1–9. doi:10.1186/1750-1326-6-63

19 Palop JJ, Mucke L. Amyloid-B-induced neuronal dysfunction in Alzheimer's disease: From synapses toward neural networks. Nat. Neurosci. 2010;**13**:812–8. doi:10.1038/nn.2583

20 Kashyap G, Bapat D, Das D, *et al.* Synapse loss and progress of Alzheimer's disease - A network model. *Sci Rep* 2019;**9**:1–9. doi:10.1038/s41598-019-43076-y

21 De Wilde MC, Overk CR, Sijben JW, *et al.* Meta-analysis of synaptic pathology in Alzheimer's disease reveals selective molecular vesicular machinery vulnerability. *Alzheimer's Dement* 2016;**12**:633–44. doi:10.1016/j.jalz.2015.12.005

22 Henstridge CM, Sideris DI, Carroll E, *et al.* Synapse loss in the prefrontal cortex is associated with cognitive decline in amyotrophic lateral sclerosis. *Acta Neuropathol*

For peer review only

2018;**135**:213–26. doi:10.1007/s00401-017-1797-4

23 Menkes-Caspi N, Yamin HG, Kellner V, *et al.* Pathological tau disrupts ongoing network activity. *Neuron* 2015;**85**:959–66. doi:10.1016/j.neuron.2015.01.025

24 Pinotsis DA, Schwarzkopf DS, Litvak V, *et al.* Dynamic causal modelling of lateral interactions in the visual cortex. *Neuroimage* 2013;**66**:563–76. doi:10.1016/j.neuroimage.2012.10.078

25 Adams NE, Hughes LE, Phillips HN, *et al.* GABA-ergic dynamics in human frontotemporal networks confirmed by pharmaco-magnetoencephalography. *J Neurosci* 2020;:1689–19. doi:10.1523/jneurosci.1689-19.2019

26 Sami S, Williams N, Hughes LE, *et al.* Neurophysiological signatures of Alzheimer's disease and frontotemporal lobar degeneration: pathology versus phenotype. *Brain* 2018;**141**:2500–10. doi:10.1093/brain/awy180

27 Sitnikova TA, Hughes JW, Ahlfors SP, *et al.* Short timescale abnormalities in the states of spontaneous synchrony in the functional neural networks in Alzheimer's disease. *NeuroImage Clin* 2018;**20**:128–52. doi:10.1016/j.nicl.2018.05.028

28 Puttaert D, Coquelet N, Wens V, *et al.* Alterations in resting-state network dynamics along the Alzheimer's disease continuum. *Sci Rep* 2020;**10**:1–13. doi:10.1038/s41598-020-76201-3

29 Hughes LE, Henson RN, Pereda E, *et al.* Biomagnetic biomarkers for dementia: A pilot multicentre study with a recommended methodological framework for magnetoencephalography. *Alzheimer's Dement Diagnosis, Assess Dis Monit* 2019;**11**:450–62. doi:10.1016/j.dadm.2019.04.009

30 Hughes LE, Nestor PJ, Hodges JR, *et al.* Magnetoencephalography of frontotemporal dementia: Spatiotemporally localized changes during semantic decisions. *Brain* 2011;**134**:2513–22. doi:10.1093/brain/awr196

31 Hughes LE, Rittman T, Robbins TW, *et al.* Reorganization of cortical oscillatory dynamics underlying disinhibition in frontotemporal dementia. *Brain* 2018;**141**:2486–99. doi:10.1093/brain/awy176

32 Hughes LE, Rittman T, Regenthal R, *et al.* Improving response inhibition systems in frontotemporal dementia with citalopram. *Brain* 2015;**138**:1961–75. doi:10.1093/brain/awv133

33 Hughes LE, Rowe JB. The impact of neurodegeneration on network connectivity: A study of change detection in frontotemporal dementia. *J Cogn Neurosci* 2013;**25**:802–13. doi:10.1162/jocn_a_00356

34 van Dellen E, de Waal H, van der Flier WM, *et al.* Loss of EEG Network Efficiency Is Related to Cognitive Impairment in Dementia With Lewy Bodies. *Mov Disord* 2015;**30**:1785–93. doi:10.1002/mds.26309

35 Woolrich MW, Baker A, Luckhoo H, *et al.* Dynamic state allocation for MEG source reconstruction. *Neuroimage* 2013;**77**:77. doi:10.1016/J.NEUROIMAGE.2013.03.036

36 Baker AP, Brookes MJ, Rezek IA, *et al.* Fast transient networks in spontaneous human brain activity. *Elife* 2014;**2014**. doi:10.7554/ELIFE.01867

37 Vidaurre D, Quinn AJ, Baker AP, *et al.* Spectrally resolved fast transient brain states in electrophysiological data. *Neuroimage* 2016;**126**:81–95. doi:10.1016/j.neuroimage.2015.11.047

For peer review only

38 Adams NE, Hughes LE, Rouse MA, *et al.* GABAergic cortical network physiology in frontotemporal lobar degeneration. *Brain* 2021;**144**:2135–45. doi:10.1093/BRAIN/AWAB097

39 Shaw AD, Muthukumaraswamy SD, Saxena N, *et al.* Generative modelling of the thalamo-cortical circuit mechanisms underlying the neurophysiological effects of ketamine. *Neuroimage* 2020;**221**:117189. doi:10.1016/j.neuroimage.2020.117189

40 American Psychiatric Association. *Diagnostic and Statistical Manual of Mental Disorders*. Fifth. American Psychiatric Association 2013. doi:10.1176/appi.books.9780890425596

41 Albert MS, DeKosky ST, Dickson D, *et al.* The diagnosis of mild cognitive impairment due to Alzheimer's disease: Recommendations from the National Institute on Aging-Alzheimer's Association workgroups on diagnostic guidelines for Alzheimer's disease. *Alzheimers Dement* 2011;**7**:270. doi:10.1016/J.JALZ.2011.03.008

42 McKhann GM, Knopman DS, Chertkow H, *et al.* The diagnosis of dementia due to Alzheimer's disease: Recommendations from the National Institute on Aging-Alzheimer's Association workgroups on diagnostic guidelines for Alzheimer's disease. *Alzheimer's Dement* 2011;**7**:263–9. doi:10.1016/j.jalz.2011.03.005

43 Holland D, Brewer JB, Hagler DJ, *et al.* Subregional neuroanatomical change as a biomarker for Alzheimer's disease. *Proc Natl Acad Sci U S A* 2009;**106**:20954. doi:10.1073/PNAS.0906053106

44 Pusil S, López ME, Cuesta P, *et al.* Hypersynchronization in mild cognitive impairment: the 'X' model. *Brain* 2019;**142**:3936–50. doi:10.1093/BRAIN/AWZ320

45 Fernández A, Turrero A, Zuluaga P, *et al.* Magnetoencephalographic Parietal δ Dipole Density in Mild Cognitive Impairment: Preliminary Results of a Method to Estimate the Risk of Developing Alzheimer Disease. *Arch Neurol* 2006;**63**:427–30. doi:10.1001/ARCHNEUR.63.3.427

46 Xu M, Sanz DL, Garces P, *et al.* A Graph Gaussian Embedding Method for Predicting Alzheimer's Disease Progression with MEG Brain Networks. *IEEE Trans Biomed Eng* 2021;**68**:1579–88. doi:10.1109/TBME.2021.3049199

47 Bennys K, Rondouin G, Benattar E, *et al.* Can event-related potential predict the progression of mild cognitive impairment? *J Clin Neurophysiol* 2011;**28**:625–32. doi:10.1097/WNP.0B013E31823CC2D3

48 Lai CL, Lin RT, Liou LM, *et al.* The role of event-related potentials in cognitive decline in Alzheimer's disease. *Clin Neurophysiol* 2010;**121**:194–9. doi:10.1016/J.CLINPH.2009.11.001

49 Fruehwirt W, Dorffner G, Roberts S, *et al.* Associations of event-related brain potentials and Alzheimer's disease severity: A longitudinal study. *Prog Neuropsychopharmacol Biol Psychiatry* 2019;**92**:31–8. doi:10.1016/J.PNPBP.2018.12.013

50 Papaliagkas VT, Kimiskidis VK, Tsolaki MN, *et al.* Cognitive event-related potentials: Longitudinal changes in mild cognitive impairment. *Clin Neurophysiol* 2011;**122**:1322–6. doi:10.1016/J.CLINPH.2010.12.036

51 Chapman RM, McCrary JW, Gardner MN, *et al.* Brain ERP components predict which

For peer review only

individuals progress to Alzheimer's disease and which do not. *Neurobiol Aging* 2011;**32**:1742–55. doi:10.1016/J.NEUROBIOLAGING.2009.11.010

52 Papaliagkas V, Kimiskidis V, Tsolaki M, *et al.* Usefulness of event-related potentials in the assessment of mild cognitive impairment. *BMC Neurosci* 2008;**9**:1–10. doi:10.1186/1471-2202-9-107/TABLES/4

53 Mandal PK, Banerjee A, Tripathi M, *et al.* A Comprehensive Review of Magnetoencephalography (MEG) Studies for Brain Functionality in Healthy Aging and Alzheimer's Disease (AD). *Front Comput Neurosci* 2018;**12**:60. doi:10.3389/FNCOM.2018.00060

54 Salgado JF. Transforming the area under the normal curve (AUC) into cohen's d, pearson's rpb, odds-ratio, and natural log odds-ratio: Two conversion tables. *Eur J Psychol Appl to Leg Context* 2018;**10**:35–47. doi:10.5093/EJPALC2018A5

55 Erdfelder E, FAul F, Buchner A, *et al.* Statistical power analyses using G*Power 3.1: Tests for correlation and regression analyses. *Behav Res Methods* 2009;**41**:1149–60. doi:10.3758/BRM.41.4.1149

56 Ossenkoppele R, Jansen WJ, Rabinovici GD, *et al.* Prevalence of Amyloid PET Positivity in Dementia Syndromes: A Meta-analysis HHS Public Access. *JAMA* 2015;**313**:1939–49. doi:10.1001/jama.2015.4669

57 Monsell SE, Kukull WA, Roher AE, *et al.* Characterizing apolipoprotein E ε4 carriers and noncarriers with the clinical diagnosis of mild to moderate Alzheimer dementia and minimal β-amyloid peptide plaques. *JAMA Neurol* 2015;**72**:1124–31. doi:10.1001/jamaneurol.2015.1721

58 Sabri O, Sabbagh MN, Seibyl J, *et al.* Florbetaben PET imaging to detect amyloid beta plaques in Alzheimer's disease: Phase 3 study. *Alzheimer's Dement* 2015;**11**:964–74. doi:10.1016/j.jalz.2015.02.004

59 Rowe CC, Doré V, Jones G, *et al.* 18F-Florbetaben PET beta-amyloid binding expressed in Centiloids. *Eur J Nucl Med Mol Imaging* 2017;**44**:2053–9. doi:10.1007/s00259-017-3749-6

60 Mioshi E, Dawson K, Mitchell J, *et al.* The Addenbrooke's Cognitive Examination revised (ACE-R): A brief cognitive test battery for dementia screening. *Int J Geriatr Psychiatry* 2006;**21**:1078–85. doi:10.1002/gps.1610

61 Folstein MF, Folstein SE, McHugh PR. 'Mini-mental state'. A practical method for grading the cognitive state of patients for the clinician. *J Psychiatr Res* 1975;**12**:189–98. doi:10.1016/0022-3956(75)90026-6

62 Hughes CP, Berg L, Danziger WL, *et al.* A new clinical scale for the staging of dementia. *Br J Psychiatry* 1982;**140**:566–72. doi:10.1192/bjp.140.6.566

63 Morris JC. The clinical dementia rating (cdr): Current version and scoring rules. *Neurology* 1993;**43**:2412–4. doi:10.1212/wnl.43.11.2412-a

64 Washington University Alzheimer's Disease Research Center. The CDR® Dementia Staging Instrument. SCALE. 1999;:1. doi:20 May 1999

65 Rosen WG, Terry RD, Fuld PA, *et al.* Pathological verification of ischemic score in differentiation of dementias. *Ann Neurol* 1980;**7**:486–8. doi:10.1002/ana.410070516

66 Ishihara T, Terada S. [Geriatric Depression Scale (GDS)]. *Nihon Rinsho* 2011;**69 Suppl 8**:455–8. doi:10.1300/j018v05n01_09

For peer review only

67 Spielberger CD, Gorsuch RL, Lushene RE. Manual for the State-Trait Anxiety Inventory. 1970.

68 Buysse DJ, Reynolds CF, Monk TH, *et al.* The Pittsburgh sleep quality index: A new instrument for psychiatric practice and research. *Psychiatry Res* 1989;**28**:193–213. doi:10.1016/0165-1781(89)90047-4

69 Jutten RJ, Peeters CFW, Leijdesdorff SMJ, *et al.* Detecting functional decline from normal aging to dementia: Development and validation of a short version of the Amsterdam IADL Questionnaire. *Alzheimer's Dement Diagnosis, Assess Dis Monit* 2017;**8**:26–35. doi:10.1016/j.dadm.2017.03.002

70 Ismail Z, Agüera-Ortiz L, Brodaty H, *et al.* The Mild Behavioral Impairment Checklist (MBI-C): A Rating Scale for Neuropsychiatric Symptoms in Pre-Dementia Populations. *J Alzheimer's Dis* 2017;**56**:929–38. doi:10.3233/JAD-160979

71 Koychev I, Lawson J, Chessell T, *et al.* Deep and Frequent Phenotyping study protocol: an observational study in prodromal Alzheimer's disease. *BMJ Open* 2019;**9**:24498. doi:10.1136/bmjopen-2018-024498

72 Solomon A, Kivipelto M, Molinuevo JL, *et al.* European Prevention of Alzheimer's Dementia Longitudinal Cohort Study (EPAD LCS): Study protocol. *BMJ Open* 2018;**8**. doi:10.1136/bmjopen-2017-021017

73 Ritchie K, Ropacki M, Albala B, *et al.* Recommended cognitive outcomes in preclinical Alzheimer's disease: Consensus statement from the European Prevention of Alzheimer's Dementia project. Alzheimer's Dement. 2017;**13**:186–95. doi:10.1016/j.jalz.2016.07.154

74 Donohue MC, Sperling RA, Salmon DP, *et al.* The preclinical Alzheimer cognitive composite: Measuring amyloid-related decline. *JAMA Neurol* 2014;**71**:961–70. doi:10.1001/jamaneurol.2014.803

75 Randolph C, Tierney MC, Mohr E, *et al.* The Repeatable Battery for the Assessment of Neuropsychological Status (RBANS): Preliminary clinical validity. *J Clin Exp Neuropsychol* 1998;**20**:310–9. doi:10.1076/jcen.20.3.310.823

76 Wechsler D. *Wechsler Adult Intelligence Scale - Revised.* San Antonio, TX: Psychological Corporation 1981.

77 Grober E, Veroff AE, Lipton RB. Temporal unfolding of declining episodic memory on the Free and Cued Selective Reminding Test in the predementia phase of Alzheimer's disease: Implications for clinical trials. *Alzheimer's Dement Diagnosis, Assess Dis Monit* 2018;**10**:161–71. doi:10.1016/j.dadm.2017.12.004

78 Wechsler D. *Wechsler Memory Scale - Third UK Edition.* London: Harcourt Assessment 1999.

79 Nelson HE, Willison JR. *National Adult Reading Test (NART): Test Manual (2nd edn).* 1991.

80 Bowie CR, Harvey PD. Administration and interpretation of the Trail Making Test. *Nat Protoc* 2006;**1**:2277–81. doi:10.1038/nprot.2006.390

81 Cambridge Cognition. CANTAB® [Cognitive assessment software]. 2019.

82 Hartley T, Bird CM, Chan D, *et al.* The hippocampus is required for short-term topographical memory in humans. *Hippocampus* 2007;**17**:34–8. doi:10.1002/hipo.20240

For peer review only

83 Kiebel SJ, Garrido MI, Friston KJ. Dynamic causal modelling of evoked responses: The role of intrinsic connections. *Neuroimage* 2007;**36**:332–45. doi:10.1016/j.neuroimage.2007.02.046

84 Friston K. A theory of cortical responses. *Philos Trans R Soc B Biol Sci* 2005;**360**:815–36. doi:10.1098/rstb.2005.1622

85 De Rover M, Pironti VA, McCabe JA, *et al.* Hippocampal dysfunction in patients with mild cognitive impairment: A functional neuroimaging study of a visuospatial paired associates learning task. *Neuropsychologia* 2011;**49**:2060–70. doi:10.1016/j.neuropsychologia.2011.03.037

86 Blackwell AD, Sahakian BJ, Vesey R, *et al.* Detecting dementia: Novel neuropsychological markers of preclinical Alzheimer's disease. *Dement Geriatr Cogn Disord* 2004;**17**:42–8. doi:10.1159/000074081

87 Tournier JD, Smith R, Raffelt D, *et al.* MRtrix3: A fast, flexible and open software framework for medical image processing and visualisation. Neuroimage. 2019;**202**. doi:10.1016/j.neuroimage.2019.116137

For peer review only

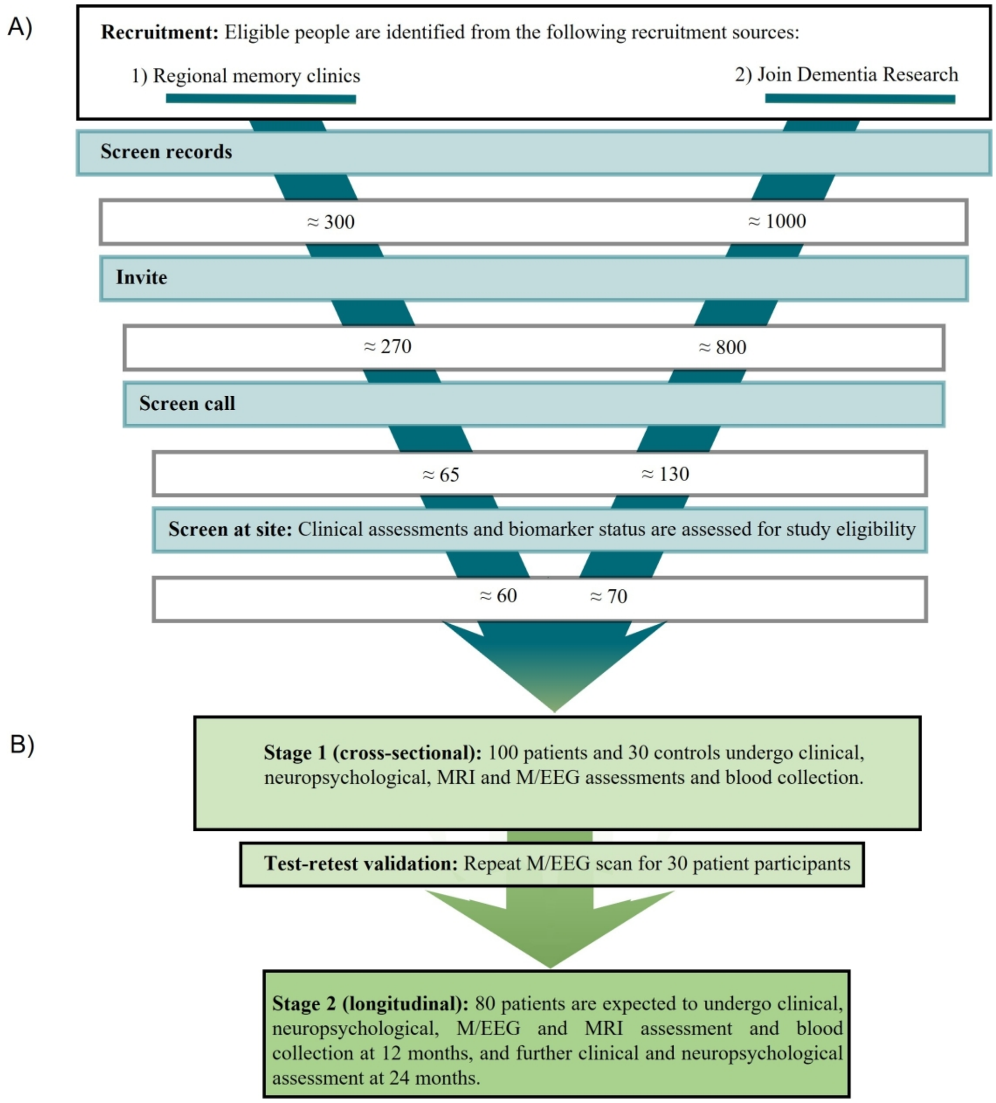

Participant flow chart. A) Recruitment strategy: Electronic screening of records from regional memory clinics and Join Dementia Research generates approximately 300 potential patient and 1000 potential patient and control participants who are invited to telephone screening. People who remain eligible are screened further on-site to identify 100 patient and 30 control participants. B) Study stages: Stage 1 consists of the cross-sectional baseline assessments and, for 30 patients, a repeat of the M/EEG assessment two weeks after the first. Only patients continue to the longitudinal stage 2 of the study, with repeat assessments 12 and 24 months after baseline. Attrition of 20% is expected at stage 2. M/EEG=Magnetoencephalography combined with electroencephalography imaging, MRI=Magnetic resonance imaging

170x188mm (330 x 330 DPI)

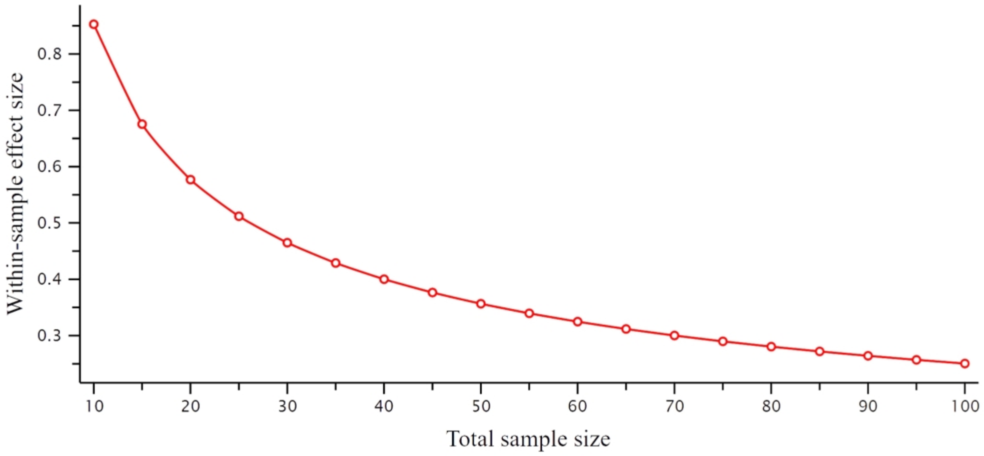

Effect sizes required to detect longitudinal correlations with disease severity at different attrition rates (resulting in reduced longitudinal sample size) with 80% power and α=0.05. Effect sizes were estimated using G*power3.1 software for one-tailed, paired t-tests . We anticipate <20% attrition per annum.

445x209mm (300 x 300 DPI)

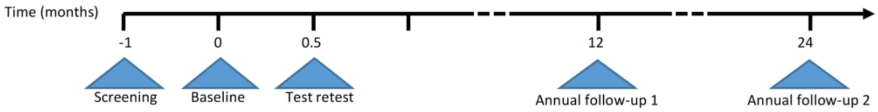

	Screening	Baseline	Test retest		Annual follow-up 1		Annual follow-up 2
Blood collection	X				X		
Biomarker samples	X						
Clinical assessments	X				X		X
Neuropsychological assessments		X			X		X
EEG and MEG scan		X	X		X		
MRI scan		X			X		

Timeline summary of study procedures

336x159mm (330 x 330 DPI)

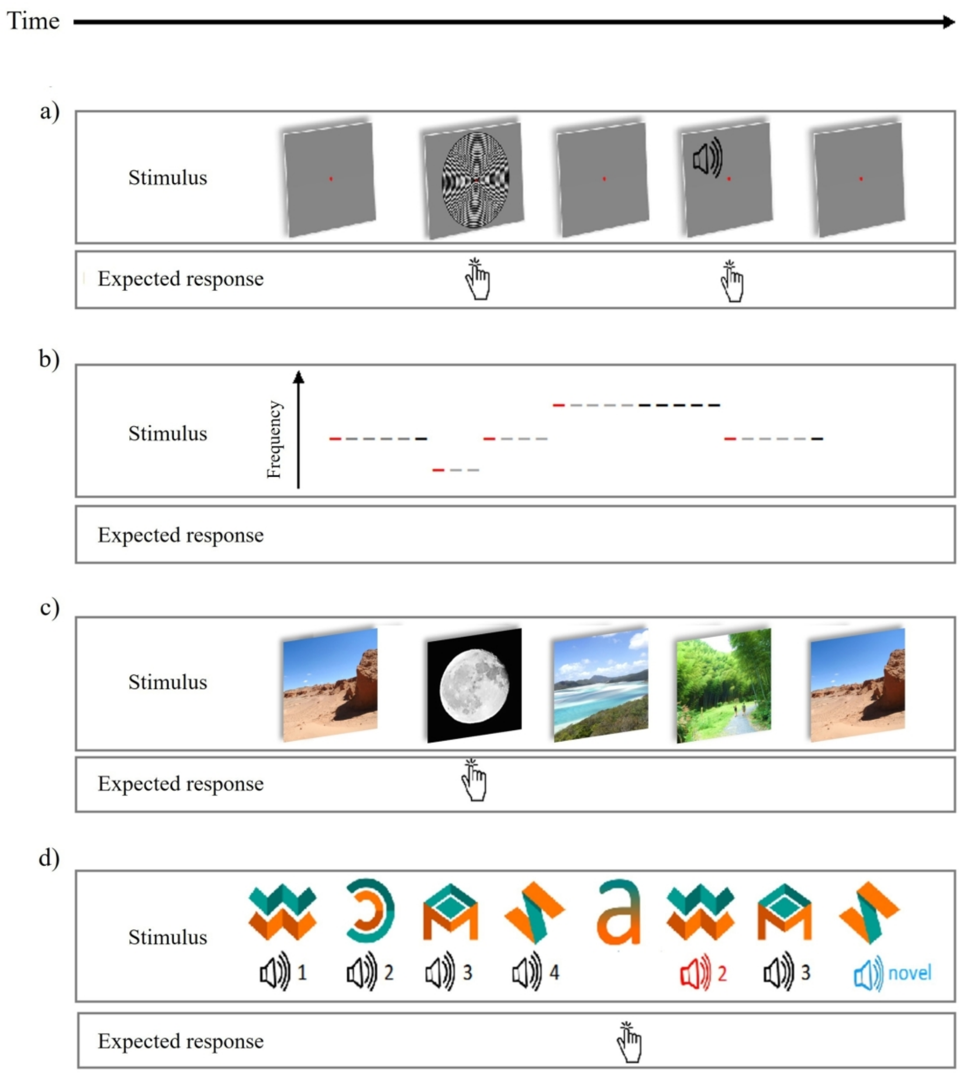

a) Audio-visual task: Participants visually fixate on a red dot and press a button whenever they hear or see something. (b) Mismatch-negativity task: Participants passively watch a nature documentary while listening to tones through the earpieces. Red dashes represent deviant tones and black dashes represent standard tones (from the sixth repetition). (c) Scene repetition task: Participants view scenes and press the button when they see a scene containing a moon. Each non-target scene repeats once during the task. Images in the figure are for illustrative purposes only. (d) Cross-modal oddball task: Participants view object-tone pairs consisting of 4 learnt (standard) pairs (the standard sounds are black in the figure); associative-deviant, object-tone pairs (the associative deviant sound is coloured red in the figure); and standard objects presented with novel tones (the novel deviant tones are blue in the figure). Participants press a button when they see the letter 'a'. The visual objects were designed by Freepik.

190x208mm (300 x 300 DPI)

STROBE Statement—Checklist of items that should be included in reports of ***cohort studies***

NB result and discussion sections are less relevant as this is for an observational protocol paper

	Item No	Recommendation	Page No
**Title and abstract**	1	(*a*) Indicate the study's design with a commonly used term in the title or the abstract	1
		(*b*) Provide in the abstract an informative and balanced summary of what was done and what was found	2
**Introduction**			
Background/rationale	2	Explain the scientific background and rationale for the investigation being reported	3
Objectives	3	State specific objectives, including any prespecified hypotheses	4
**Methods**			
Study design	4	Present key elements of study design early in the paper	5
Setting	5	Describe the setting, locations, and relevant dates, including periods of recruitment, exposure, follow-up, and data collection	7
Participants	6	(*a*) Give the eligibility criteria, and the sources and methods of selection of participants. Describe methods of follow-up	9-15
		(*b*) For matched studies, give matching criteria and number of exposed and unexposed	
Variables	7	Clearly define all outcomes, exposures, predictors, potential confounders, and effect modifiers. Give diagnostic criteria, if applicable	8-15
Data sources/ measurement	8*	For each variable of interest, give sources of data and details of methods of assessment (measurement). Describe comparability of assessment methods if there is more than one group	NA
Bias	9	Describe any efforts to address potential sources of bias	NA
Study size	10	Explain how the study size was arrived at	7
Quantitative variables	11	Explain how quantitative variables were handled in the analyses. If applicable, describe which groupings were chosen and why	4
Statistical methods	12	(*a*) Describe all statistical methods, including those used to control for confounding	4
		(*b*) Describe any methods used to examine subgroups and interactions	
		(*c*) Explain how missing data were addressed	
		(*d*) If applicable, explain how loss to follow-up was addressed	
		(*e*) Describe any sensitivity analyses	
**Results**			
Participants	13*	(a) Report numbers of individuals at each stage of study—eg numbers potentially eligible, examined for eligibility, confirmed eligible, included in the study, completing follow-up, and analysed	Fig 1
		(b) Give reasons for non-participation at each stage	
		(c) Consider use of a flow diagram	
Descriptive data	14*	(a) Give characteristics of study participants (eg demographic, clinical, social) and information on exposures and potential confounders	NA
		(b) Indicate number of participants with missing data for each variable of interest	
		(c) Summarise follow-up time (eg, average and total amount)	
Outcome data	15*	Report numbers of outcome events or summary measures over time	NA

For peer review only - http://bmjopen.bmj.com/site/about/guidelines.xhtml

Main results	16	(*a*) Give unadjusted estimates and, if applicable, confounder-adjusted estimates and their precision (eg, 95% confidence interval). Make clear which confounders were adjusted for and why they were included<br>(*b*) Report category boundaries when continuous variables were categorized<br>(*c*) If relevant, consider translating estimates of relative risk into absolute risk for a meaningful time period	NA
Other analyses	17	Report other analyses done—eg analyses of subgroups and interactions, and sensitivity analyses	NA
**Discussion**			
Key results	18	Summarise key results with reference to study objectives	NA
Limitations	19	Discuss limitations of the study, taking into account sources of potential bias or imprecision. Discuss both direction and magnitude of any potential bias	NA
Interpretation	20	Give a cautious overall interpretation of results considering objectives, limitations, multiplicity of analyses, results from similar studies, and other relevant evidence	NA
Generalisability	21	Discuss the generalisability (external validity) of the study results	NA
**Other information**			
Funding	22	Give the source of funding and the role of the funders for the present study and, if applicable, for the original study on which the present article is based	21

*Give information separately for exposed and unexposed groups.

Note: An Explanation and Elaboration article discusses each checklist item and gives methodological background and published examples of transparent reporting. The STROBE checklist is best used in conjunction with this article (freely available on the Web sites of PLoS Medicine at http://www.plosmedicine.org/, Annals of Internal Medicine at http://www.annals.org/, and Epidemiology at http://www.epidem.com/). Information on the STROBE Initiative is available at http://www.strobe-statement.org.

For peer review only

For peer review only - http://bmjopen.bmj.com/site/about/guidelines.xhtml
